# Pulsed electric field induces exocytosis and overexpression of MAGE antigens in melanoma

**DOI:** 10.1038/s41598-024-63181-x

**Published:** 2024-05-31

**Authors:** Wojciech Szlasa, Natalia Sauer, Dagmara Baczyńska, Marcin Ziętek, Katarzyna Haczkiewicz-Leśniak, Paweł Karpiński, Mariusz Fleszar, Paulina Fortuna, Michał J. Kulus, Aleksandra Piotrowska, Alicja Kmiecik, Agnieszka Barańska, Olga Michel, Vitalij Novickij, Mounir Tarek, Paulina Kasperkiewicz, Piotr Dzięgiel, Marzenna Podhorska-Okołów, Jolanta Saczko, Julita Kulbacka

**Affiliations:** 1 Medical University Hospital, Borowska 213, 50-556 Wrocław, Poland; 2 Faculty of Pharmacy, Wroclaw Medical University, Wroclaw, Poland; 3 Department of Molecular and Cellular Biology, Faculty of Pharmacy, Wroclaw Medical University, Wroclaw, Poland; 4 Department of Surgical Oncology, Wroclaw Comprehensive Cancer Center, Wroclaw, Poland; 5 Division of Ultrastructural Research, Faculty of Medicine, Wroclaw Medical University, 50-368 Wroclaw, Poland; 6 Department of Genetics, Wroclaw Medical University, Wroclaw, Poland; 7 Division of Histology and Embryology, Department of Human Morphology and Embryology, Wroclaw, Poland; 8 Department of Medical Biochemistry, Wroclaw Medical University, Wroclaw, Poland; 9 Faculty of Electronics, Vilnius Gediminas Technical University, 03227 Vilnius, Lithuania; 10 Université de Lorraine, CNRS, LPCT, 54000 Nancy, France; 11 Department of Chemical Biology and Bioimaging, Faculty of Chemistry, Wroclaw University of Science and Technology, Wroclaw, Poland; 12 Department of Immunology, State Research Institute Centre for Innovative Medicine, Santariškių 5, 08410 Vilnius, Lithuania; 13 Omics Research Center, Wroclaw Medical University, Wrocław, Poland

**Keywords:** Melanoma, Nanosecond pulsed electric field, Microvesicles, MAGE, PD-1, Focal treatment, Exocytosis, Molecular medicine, Cancer, Surgical oncology

## Abstract

Nanosecond pulsed electric field (nsPEF) has emerged as a promising approach for inducing cell death in melanoma, either as a standalone treatment or in combination with chemotherapeutics. However, to date, there has been a shortage of studies exploring the impact of nsPEF on the expression of cancer-specific molecules. In this investigation, we sought to assess the effects of nsPEF on melanoma-specific MAGE (Melanoma Antigen Gene Protein Family) expression. To achieve this, melanoma cells were exposed to nsPEF with parameters set at 8 kV/cm, 200 ns duration, 100 pulses, and a frequency of 10 kHz. We also aimed to comprehensively describe the consequences of this electric field on melanoma cells' invasion and proliferation potential. Our findings reveal that following exposure to nsPEF, melanoma cells release microvesicles containing MAGE antigens, leading to a simultaneous increase in the expression and mRNA content of membrane-associated antigens such as MAGE-A1. Notably, we observed an unexpected increase in the expression of PD-1 as well. While we did not observe significant differences in the cells' proliferation or invasion potential, a remarkable alteration in the cells' metabolomic and lipidomic profiles towards a less aggressive phenotype was evident. Furthermore, we validated these results using ex vivo tissue cultures and 3D melanoma culture models. Our study demonstrates that nsPEF can elevate the expression of membrane-associated proteins, including melanoma-specific antigens. The mechanism underlying the overexpression of MAGE antigens involves the initial release of microvesicles containing MAGE antigens, followed by a gradual increase in mRNA levels, ultimately resulting in elevated expression of MAGE antigens post-experiment. These findings shed light on a novel method for modulating cancer cells to overexpress cancer-specific molecules, thereby potentially enhancing their sensitivity to targeted anticancer therapy.

## Introduction

The pulsed electric field (PEF) is currently used in various fields of molecular biology, genetics, and medicine. Some of its applications include the induction of cell death in the form of necrotic or apoptotic process, drug delivery, and gene electrotransfer (GET)^1–3^. GET and drug delivery rely on electroporation (EP)—the formation of water pores in the cell membrane^3^. When electroporation is combined with a cytotoxic drug, the process is called electrochemotherapy (ECT). In clinics, it is used to induce apoptosis in the targeted cells^4^. When the pores reseal in time, the process is called reversible electroporation. However, some applications involve the formation of irreversible pores in the membrane. Unrestricted cell swelling after permeabilization induces necrosis in the targeted cells in a process called irreversible electroporation (IRE)^5,6^. Nanosecond-range pulses find their application in the stimulation of reactive oxygen species (ROS) formation in the cells^7^. In contrast, other studies prove that microsecond pulses are mostly used to induce cell death and transmembrane electrotransfer^8,9^.

Aside from tumor-size reduction, focal therapies are known for their immunomodulatory effects^10–12^. These mostly describe the altered expression of cancer-associated molecules or immune checkpoint inhibitors^13,14^. For instance, radiotherapy was shown to promote molecular alternations in cancer cells by inducing the overexpression of cancer-associated molecules like B7-H3 or immune checkpoint inhibitors^15,16^. Nowadays, researchers claim that the PEF may be used to induce specific changes in cell metabolism and antigens present on the cell membrane. Several papers report that PEF stimulates the cells via electro-endocytosis of the cell membrane with its components^17,18^. Moreover, some papers consider the induction of specific metabolic changes in the cells that underwent PEF exposure^19^. Our previous paper showed the potential of nanosecond pulsed electric field (nsPEF) in the reduction of the multi-drug resistance effect in pancreatic cancer cells via the release of P-GP (P-Glycoprotein / MDR1) and LRP (Low-density lipoprotein receptor-related protein) from the cells in the extracellular microvesicles^20^. Presumably, the immunomodulatory effects of PEF on cancer cells might be related to the PEF-induced overexpression of specific therapeutic targets.

High expression of cancer antigens is crucial in targeted immunotherapies. Enhancing the expression of cancer-associated molecules is key to overcoming one of the major challenges of novel therapies for solid tumors—the immune escape mechanism of the neoplasm. Cancer cells can downregulate targets of these therapies, becoming less visible to the immune system's cells^21^. Therefore, high abundance of cancer antigens increases the effectiveness of the therapy^22,23^. At the same time, enhanced expression of PD-1 results in the attenuation of the immune response to cancer^24^. Knowing that focal therapies induce the overexpression of immune checkpoint inhibitors^14,25^, in this work we examined the expression of PD-1 alongside the cancer molecules. There is a strong foundation for the investigation of methods to improve the presentation of the cancer-specific targets on the tumor’s surface^26–28^. Most solid tumors lack cancer-specific molecules on their cell membranes^29^, thus immunotherapy is mostly applied in hematology to treat leukemias and lymphomas^30,31^. Interestingly, embryonic cells are involved in the development of both lymphatic system cells as well as melanocytes^32,33^, thus due to the similar origin to lymphatic cells, melanoma is extensively searched for potential cancer-specific targets. Such examples may be recognized in the MAGE family of proteins. MAGE-C2 is expressed in the cytoplasm in well-differentiated hepatocellular carcinoma and the nucleus in moderately- and poorly differentiated cancer^34^. MAGE-A group is heterogeneous and comprises membrane and cytosol-grounded molecules^35–37^. The latter antigens may be recognized in melanoma by autologous cytolytic T-lymphocytes^38,39^. Aside from melanoma, the protein may be found in the placenta, and seminiferous tubules’ cells as well as in head and neck squamous cell carcinoma, lung carcinoma, and breast carcinoma^40,41^. MAGE family members are involved in embryonic development and tumor progression^42,43^. MAGE-A1 interacts with Ski Interacting Protein (SKIP), recruiting histone deacetylase (HDAC1)^44^. The protein may downregulate notch intracellular domain transactivation. On the other hand, MAGE-A3 increases the ubiquitin ligase activity of E3 ubiquitin-protein ligase and TRIM28^45,46^. Due to the high number of the members of MAGE-A antigens group and the various roles they exert, the detailed function of the antigens is not yet fully understood. High expression of MAGE antigens correlates with the positive response of patients to the therapy and may be probably used in the targeted therapy of cancer^47^. However, MAGE antigens overexpressed on other tumors than melanoma are the markers for poor prognosis for cancer patients^48,49^. Therefore, MAGE antigens might be considered as the specific target for various immunotherapy types^50,51^.

Combining all the data, we hypothesize that by subjecting melanoma cells to nsPEF we may induce positive changes in cells’ metabolism and immunophenotype. Knowing that focal therapies highly affect the expression of cancer-associated molecules and immune checkpoint inhibitors, we aim to investigate these effects and their mechanisms upon treatment of melanoma cells with nsPEF. In the study, initially, the viability and permeability of the cells were assessed following the application of electric pulses. Afterward, A375 melanoma cells underwent fluorescent staining to analyze the signal localization and intensity of MAGE-A1, MAGE-C2, and MAGE-3 antigens. Based on the screening studies, we proposed the electric field protocol that enhances MAGE membrane presentation. Western blot studies included the assessment of cross-reacting MAGE-A2, MAGE-A6, MAGE-A8, MAGE-A9, MAGE-A10, and MAGE-A12 on melanoma cells. Besides, we also evaluated the expression of PD-1, which plays a critical role in the inhibition of melanoma growth and cell-to-cell stimulatory interactions^24^. Via confocal laser microscopy, holotomographic imaging, and electron microscopy studies we revealed the detailed mechanism of the role of nsPEF on melanoma cells. To characterize the metabolic status of the cells following nsPEF exposure, we conducted metabolomic, lipidomic studies and functional cell tests. In the end, we evaluated the proposed therapeutic protocol on 3D cancer spheroids and ex-vivo tissue cultures from melanoma patients. The study is a foundation for the future application of nsPEF as the adjuvant technique for melanoma therapy.

## Materials and methods

### Cell culture

The melanotic melanoma A375 (ATCC® CRL-1619™) cell line was obtained from the skin of a 54-year-old female patient. The C32 (ATCC® CRL-1585™)—amelanotic melanoma cell line derived from the skin tissue of a 53-year-old male Caucasian patient. The MeWo (ATCC® HTB-65™)—malignant melanoma cell line derived from the metastatic lymph node site of a 78-year-old white male patient. The Colo 829 (ATCC® CRL-1974™) was derived from a 45-year-old white male patient from a biopsy specimen taken before chemotherapy for metastatic melanoma. The Me45 cell line was obtained from the Oncology Centre Gliwice, where the cells were derived from a 35-year-old woman’s lymph node cells. Cells were cultured as a monolayer in Dulbecco’s Modified Eagle’s Medium (DMEM, Sigma-Aldrich, St. Louis, MO, USA) in a monolayer on a plastic flask 25 and 75 cm^2^ (Nunc, Denmark). The medium was supplemented with 10% fetal bovine serum (FBS, Sigma-Aldrich), an antibiotic (Penicillin–Streptomycin 10,000 U/mL, 15140122 Gibco), 1 mM ultraglutamine (Sigma-Aldrich, Poznan, Poland) and 1% minimal essential vitamins (Mem-Vit, Sigma-Aldrich, Poznan, Poland). The cells were incubated at 37 °C in a humidified atmosphere containing 5% CO_2_. When needed, the cells were washed with PBS and removed by trypsinization (0.025% trypsin and 0.02% EDTA; Sigma-Aldrich).

### 3D cell culture

The generation of 3D melanoma spheroids was conducted using a RCCS (Rotary Cell Culture System, Synthecon, Huston, TX) centrifuge designed to facilitate the formation of small cancer tumors. The cells were initially cultured in a rotating vessel filled with 10 ml of fully supplemented DMEM (DMEM, Sigma-Aldrich, St. Louis, MO, USA) cell culture media, to promote spheroid formation. Subsequently, 10 ml of the cell suspension (1 million cells / ml) was carefully loaded into the RCCS, which provided a controlled environment for spheroid development. The chamber's rotation ensured the even distribution of cells and promoting the aggregation of melanoma cells into compact, spherical structures that mimic small cancer tumors. The spheroids were maintained for 12 h in the RCCS under standard cell culture conditions, including temperature, humidity, and CO_2_ levels, to support their growth and structural integrity. Next, the 3D cell tumors were gently moved to the 6-well plates (Thermo Fisher, USA) for PEF experiments and incubation.

### Electric field exposure

The square wave electroporator (100 ns–1 ms) developed in the Institute of High Magnetic Fields (VGTU, Vilnius, Lithuania) was used to deliver electric pulses^52^. Electroporation cuvettes (VWR) with an electrode gap of 1 mm (BTX, Syngen Biotech, Poland) were used in the procedure. The cells were detached from the culture flasks by trypsinization (0.025% trypsin and 0.02% EDTA; Sigma-Aldrich). Next, cells were suspended in a cuvette using a 10 mM phosphate buffer (pH = 7.4, 1 mM MgCl_2,_ and 250 mM sucrose). Afterward, the samples were subjected to the PEFs. Then, the electroporation buffer was replaced with a culture medium (DMEM, Sigma-Aldrich, St. Louis, MO, USA), and the cells (5 × 10^4^ cells per well on 96-well plate and 1 × 10^6^ cells per well on 6-well plate) were seeded on culture plates (96-well plates for viability tests and 6-well plates for spharoid and ex-vivo experiments, Thermo Fisher, USA). For the holotomographic microscopy studies, we used attached cells and treated them using the needle electrodes. For different experiments we used different incubation times after subjecting the cells to PEF. Viability tests were performed 24 h after PEF treatment, Boyden chamber migration was assessed 8 h following the PEF experiment, adhesive properties were assessed 2 h following PEF experiment and fluorescence microscopy experiments were preformed 24 h after exposure to PEF. 3D cell cultures were incubated in cell culture medium 24 h after the experiment.

### Yo-pro-1 uptake studies

The permeabilization of the melanoma cells in response to PEF exposures was analyzed by flow cytometry (Cube-6, Sysmex EUROPE GmbH, Warsaw, Poland). The cells were detached from the culture flasks with trypsin, centrifuged, and suspended in electroporation phosphate buffer (10 mM Na_2_HPO_4_/NaH_2_PO_4_, 1 mM MgCl_2_, 250 mM sucrose, pH 7.4). Cells were maintained in suspension and pulsed in a cuvette (VWR) with two aluminum plate electrodes (1 mm gap). 30 s before subjecting the cells to electric field, melanoma cells were suspended in Yo-Pro-1 solution in the phosphate buffer (final concentration 1 µL/1 mL). After PEF exposure, the cells were incubated for 3 min in room temperature (20 °C). In the next step, cells were resuspended in 0.3 mL of PBS. Flow cytometry analysis was performed using a Cube 6 flow cytometer (Sysmex, Warsaw, Poland). The fluorescence of Yo-Pro-1 was excited with a 488 nm laser and assessed with the FL-3 detector (700/50). Data was collected and analyzed by CyView software (Sysmex, Warsaw, Poland).

### MTT viability assay

The viability of the cells 24 h after the experiment was analyzed by mitochondrial activity assay. The culture medium was removed from each of the wells, and 100 μL of 0.5 mg/mL MTT (3-(4,5-dimethylthiazol-2-yl)-2,5-diphenyltetrazolium bromide, Sigma-Aldrich) solution in PBS buffer was added. After 2 h of incubation at 37 °C, acidified isopropanol (100 μL, 0.04 M HCl in 99.9% isopropanol) was added to dissolve the formazan crystals. The samples were fully dissolved by the pipet mixing technique. The absorbance of each well was measured at 570 nm using a multiplate reader (GloMax, Promega, Walldorf, Germany). The results were expressed as the percentage of viable cells relative to control cells.

### SRB assay

24 h after the experiment, the medium was removed from the cells and 50 µL of 50% cold trichloroacetic acid (TCA, Sigma-Aldrich) was added to each well and incubated for 1 h at 4 °C. Afterwards, the plate was washed with water and then dried. 50 µL of 0.4% sulforhodamine B (SRB, Sigma-Aldrich) solution in 1% acetic acid (Sigma-Aldrich) was added to each well. 30 min of incubation was performed at room Temperature. Further, the plate was washed 5 times with 1% acetic acid (Sigma-Aldrich) and dried. Finally, 150 µL of 10 mM TRIS solution (Sigma-Aldrich) was added to each well. The absorbance reading at 492 nm wavelength was performed with a Promega microplate reader (GloMax® Discover, Promega, GmbH, Germany).

### Caspase 3/7 activity assay

The Caspase-Glo 3/7 Assay System (Promega, Madison, WI, USA) was utilized to assess the activity of caspase 3/7 in melanoma cells. These cells were planted in 96-well plates and incubated for 24 h following the PEF treatment. Following the treatment, each well received 100 µL of Caspase-Glo 3/7 reagent and was incubated at room temperature for 1 h. The luminescence generated was then measured using a GloMax Reader (Promega, Madison, WI, USA). The results were presented as a percentage, comparing the caspase 3/7 activity in treated cells to that in untreated control cells.

### Fluorescent studies

Anti-MAGE-A1 antibody (1:500, EPR4276, ab247762, Abcam, USA), anti-MAGE-3 antibody (1:1000, EPR19065, ab223162, Abcam, USA), and anti-MAGE-C2 antibody (1:1000, EPR19064, ab209667, Abcam, USA) were applied to assess the level of MAGE antigens 24 h following nsPEF exposure. After the experiment, the cells were washed with PBS and fixed with 4% formalin. As soon as three replicates were performed, the single staining procedure was performed to obtain similar fluorescence between the same samples from different replicates. The samples were initially treated with Triton-X100 (Sigma, Poland) for 5 min to permeabilize the membranes. Following, the incubation with FBS was performed for 1 h in 37 °C. Next, the cells were washed with Triton-X100, and the primary antibody was added for 1 h incubation in 37 °C. Following, the cells were washed with PBS and the secondary antibody (AlexaFluor 488™, Invitrogen, USA) was added for 1 h incubation in 37 °C. In the end, the samples were washed with PBS. The samples were observed on the Olympus BX53F2 microscope (40x, Olympus, Tokyo, Japan) after blue laser excitation (488 nm, Olympus, Tokyo, Japan). The mean fluorescence of the cells on each sample was analyzed in the ImageJ software. From each photograph, cells were analyzed. Each sample consisted of at least 3 photographs. From each analyzed cell, the background fluorescence was subtracted. Data was plotted and analyzed.

### Adhesion assay

Cell adhesion assay was conducted using Vybrant™ Cell Adhesion Assay Kit (ThermoFisher, V13181) according to the manufacturer's protocol. Calcein AM localizes within cells, facilitating the quantification of cell adhesion. Our protocol involved the incubation period to 1 h to enhance cell-plate interactions. Post-incubation, non-adherent cells were removed, and the degree of adhesion was quantified by measuring the fluorescence at 517 nm, providing a reliable correlation with the number of adherent cells.

### Migration assay

Cell migration was assessed using the Boyden Chamber assay (Sigma-Aldrich). This technique utilizes a small, hollow plastic chamber sealed at one end with a porous membrane, suspended above a larger well. Cells were initially cultured under standard conditions and harvested at confluence. For the assay, cells were resuspended in a serum-free medium (fully supplemented as described before but without FBS) and seeded into the upper chamber of the Boyden apparatus. The lower well was filled with medium supplemented with fully supplemented 10% FBS DMEM to induce cell migration. The setup was incubated for 8 h, allowing cells to migrate through the membrane pores. Post-incubation, migratory cells on the lower surface of the membrane were fixed, stained with Hoechst (Thermo Fisher Scientific, Waltham, MA), and quantified. Non-migratory cells on the upper surface were removed before staining. The extent of cell migration was determined by counting the stained cells, and data were analyzed.

### Molecular dynamics simulation

The molecular dynamics simulations were performed with GROMACS 2018.3 software^53^ on the calculational cluster in the Department of Theoretical Chemistry and Physics at Lorraine University. The models for simulations were built with CHARMM-GUI web software and visually inspected with VMD software^54,55^. The simulated systems were composed of a membrane in the ionic water solution. The membrane was composed of ~ 256 lipids per membrane layer. All the systems were built considering a mixture of cholesterol and 1-palmitoyl-2-oleoyl-sn-glycero-3-phosphocholine (POPC). A system representing 10% cholesterol and POPC was modeled. Before the simulation, the system was solvated in physiological conditions of NaCl water (TIP3) solution. Six calcium ions were present in each system. Both water compartments were separated by the introduction of the vacuum above and below the system. The whole simulation was performed in periodic boundary conditions. The simulation proceeded with the CHARMM36 force field^56^. The systems were minimized, equilibrated (100 ns, NPT conditions: Nose–Hoover thermostat and Berendsen barostat). Afterwards, the system was simulated for 100 ns under various electric field intensity conditions. The electric field simulation was carried out under NPγT conditions (constant surface tension, number of molecules, pressure, and temperature). After the simulation, the system was evaluated if the pore was formed during the time of the simulation and the visual representations of the membrane were presented on the layouts.

### Western blotting

Western blot technique (WB) was used to determine MAGE-A1, MAGE-A2, MAGE-A3, MAGE-A6, MAGE-A8, MAGE-A12 and MAGE-C2 expression in A375, C32, MeWo, Colo-829, Me45 cell cultures 24 h after PEF experiment. Whole protein lysates from the cell culture samples were obtained using the T-PER Tissue Protein Extraction Reagent (Thermo Fisher Scientific, Inc.) with the addition of the Halt™ Protease Inhibitor Cocktail (Thermo Fisher Scientific, Inc.) and 0.2 mM PMSF (Sigma-Aldrich; Merck KGaA). Protein concentrations were quantified using the BCA Protein Assay. Equal amounts of total protein (50 µg) were mixed with Laemmli sample buffer and resolved on 10% acrylamide gel by SDS-PAGE. After electrophoresis, the samples were transferred to nitrocellulose membranes (Merck KGaA) in the Transblot Biorad System. Next, the membranes were blocked in 4% bovine serum albumin solution (Merck KGaA) in TBST buffer (0.2 M Tris; 1.5 M NaCl; 0.1% Tween-20). After blocking, the membranes were incubated overnight at 4 °C with the primary rabbit anti-human anti-MAGE antibodies, diluted at 1:500 (MAGE-3, MAGE-C2) and 1:250 (MAGE-A1). Further, membranes were incubated with secondary HRP-conjugated donkey anti-rabbit antibody (715-035-152; Jackson ImmunoResearch), diluted at 1:2000 for 1 h at room temperature. Finally, the membranes were rinsed and treated with the Luminata Classico (Merck KGaA) chemiluminescent substrate. The reactions were visualized using the ChemiDoc Imaging System (Bio-Rad Laboratories). β-actin detected with primary rabbit anti-human β-actin antibody (4970; Cell Signaling Technology) diluted at 1:1000 and secondary HRP-conjugated donkey anti-rabbit antibody (711-035-152; Jackson ImmunoResearch) diluted at 1:2000 was used as an internal control. Commercially available antibodies which can recognize multiple members of the MAGE family due to shared epitopes. A densitometric analysis of the results obtained was performed with the use of the Image Lab software (Bio-Rad Laboratories). For the first blot replicates, we tested various exposure times which can be seen in the Supplementary materials. Blots were cut prior to hybridization with the indicated antibodies; hence, the original images of full-length membranes cannot be provided. We normalized the expression levels of MAGE antigens in the melanoma cell lines to β-actin, which served as an internal control. This normalization is essential for accurate comparison between PEF-treated cells and controls. We employed Western blotting to quantify protein levels, ensuring equal protein loading (50 µg per sample) and using β-actin as a consistent reference across all samples. Blotting results were analyzed with ImageJ Software. Graphical representations of the blots were prepared using Sciugo Online Software.

### Real-time PCR studies

Melanoma cell lines underwent the nsPEF experiment and were cultured for 24 h in DMEM. Next, cells were harvested from culture flasks, centrifuged for 5 min at 500×*g*, and stored for up to one week at − 20 °C for further analysis. RNA extractions from cells were performed using a NucleoSpin RNA II kit (Macherey–Nagel & Co., Düren, Germany) according to the manufacturer’s protocol with a DNAse I digestion option. 500 ng of total RNA was used for cDNA synthesis by reverse transcription reaction using a High-Capacity cDNA Reverse Transcription Kit (Thermo Fisher Scientific, Foster City, USA). Then, 3 µL of three-fold diluted RT products were added into a single real-time PCR analysis complemented with AceQ qPCR Probe Master Mix (Vazyme Biotech, China) and specific TaqMan Assays: Hs_00607097_m1 for MAGEA1, Hs_00606323_m1 for MAGEA2, Hs_00366532_m1 for both MAGEA3 and MAGEA6, Hs_00377810_m1 for MAGEA8, Hs_04176236_m1 for MAGEA12 and Hs00212255_m1 for MAGEC2 (Thermo Fisher Scientific). All reactions were performed in triplicate in 96-well plates under the following thermal cycling conditions: 3 min. at 95 °C followed by 40 cycles of 10 s at 95 °C and 30 s at 60 °C. The reactions were run in the TOptical Real-Time PCR Thermocycler (Biometra GmbH, Göttingen, Germany) and the threshold cycle data (Ct) were collected using qPCRsoft (Biometra GmbH, Göttingen, Germany). For relative quantification (RQ) the samples were normalized against the expression of GAPDH mRNA, using the comparative Ct method (2^−ΔΔCt^).

### Metabolomic and lipidomic profiling of melanoma cell lines

Chemicals including methanol, acetonitrile (ACN), water, chloroform, 2-propanol, and formic acid (FA) were procured from Merck Millipore (Warsaw, Poland), while leucine-enkephalin was sourced from Waters (Warsaw, Poland). For sample preparation, cells were first rinsed with PBS and subsequently exposed to a cold methanol:water:chloroform mixture (3:1:2). The resulting cell mixture was transferred to Eppendorf tubes, agitated at 4 °C for 10 min, and then frozen at -80 °C for 1 h. Subsequently, the tubes underwent centrifugation at 12,000 RCF at 4 °C for 7 min. The upper layers were collected for metabolomic analysis, while the lower layers were reserved for lipidomic analysis, both of which were dried using a centrifugal vacuum concentrator (HETOVAC) at 45 °C. The resulting precipitates were reconstituted in 50 µL of 3% methanol in water and 2-propanol:ACN:water (2:1:1) for metabolomic and lipidomic analyses, respectively. The prepared solutions were then transferred to autosampler glass vials for LC–MS analysis.

Metabolomic analysis involved the use of an Acquity ultra-high performance liquid chromatography (UHPLC) system from Waters with a reversed-phase (C18) column. A gradient elution was performed using solvent A (water containing 0.1% formic acid) and solvent B (acetonitrile containing 0.1% formic acid). Analyte separation was achieved using an ACQUITY UPLC BEH Shield RP18 1.7 µm column (2.1 × 100 mm, 1.75 µm) with a linear gradient from 3 to 97% of mobile phase B over 25.5 min at a total flow rate of 300 µL/min.

For lipidomic profiling, analytes were separated using the same column and a linear gradient from 30 to 95% of mobile phase B over 25.5 min at a total flow rate of 300 µL/min. The mobile phases consisted of 10 mM ammonium formate in ACN/methanol (60:40; v:v) for phase A and 10 mM ammonium formate in 2-propanol for phase B.

Mass spectrometry analysis was carried out with a Xevo G2 quadrupole-time of flight mass spectrometer equipped with an ESI source (Waters), operating in both positive and negative polarity modes. Data were acquired in data-independent acquisition mode (DIA) using the MSE data acquisition function. The source parameters included nebulizing and drying gas (nitrogen) at flow rates of 800 L/h and 65 L/h, respectively, a spray voltage of 2.8 kV (metabolomic analysis) or 2.5 kV (lipidomic analysis), source temperature of 120 °C, and desolvation temperature of 450 °C.

The acquired data from all four measurements were processed using MS-DIAL (ver. 4.9) software from the RIKEN Center for Sustainable Resource Science: Metabolome Informatics Research Team, which involved spectral deconvolution, peak identification, alignment, normalization, and compound identification. Subsequently, the obtained data were subjected to statistical analysis utilizing the web-based tool MetaboAnalyst 5.0 (https://www.metaboanalyst.ca).

### Transmission electron microscope (TEM) method

#### Melanoma cell line embedding in LR White resin

The pellet of the human melanoma A375 cells line was fixed in the solution of 4% formaldehyde (w/v) (Thermo Fisher Scientific, Waltham, MA, USA), and phosphate buffer saline (PBS, pH 7.4). After 30 min fixative was washed 3 times for 5 min with PBS. Next, the cells were entrapped within the fibrin clot, which was created from the droplets of bovine thrombin (lyophilizate dissolved in 5 mL of PBS, Biomed, Lublin, Poland) and fibrinogen (1 mg/mL; Merck KGaA, Darmstadt, Germany) putting to the Falcon tubes. Subsequently, to enhance the fixation process and preserve the structure of the cell membranes, the cells clots were post-fixed for 7 min in 0.25% (w/v) osmium tetroxide (SERVA Electrophoresis GmbH, Heidelberg, Germany) prepared in PBS. After the washing step, the samples were dehydrated in an ascending ethanol series (50%, 70%, 96%, 99.8% Stanlab, Lublin, Poland). The specimens thereafter were incubated using a combination of 99.8% ethanol and LR White resin (Embedding Media, Medium catalyzed, Polysciences, Inc., Warrington, PA, USA) at the following ratios: 2:1 (20 min), 1:1 (1 h) and 1:2 (1 h), respectively. The following day, after 2 changes of pure resin, the material was flat embedded in molds (Pelco, Ted Pella, Redding, Ca, USA) filled with a pure LR White resin, which polymerization took place at 55 °C. After a few days, LR White blocks were cut into 600 nm semithin sections, floated on the droplet of the water, dried on the heating plate to straighten out, stained with the toluidine blue (Serva Electrophoresis, Heidelberg, Germany), and protected from fading by locking with a mounting medium (Carl Roth, Mannheim, Germany).

#### Melanoma cell line embedding in agarose

The A375 cells were fixed in 2.5% glutaraldehyde in 0.1 M cacodylate buffer (Serva Electrophoresis, Heidelberg, Germany) for 30 min. at 37 °C. The fixed cell suspension was then embedded in 30 μL of 2% agarose at 37 °C. The solidified agarose blocks with entrapped cells were transferred to 2.5% glutaraldehyde and after 30 min washed four times at 4 °C in 0.1 M cacodylate buffer (Chempur, Piekary Śląskie, Poland), followed by fixation with 1% osmium tetroxide in 0.1 M cacodylate buffer (OsO4; 1 h, 4 °C; Serva Electrophoresis). Next, samples were washed in cacodylate buffer (4 × 5 min, 4 °C) and dehydrated in increasing ethanol solutions: 30%, 50% (2 × 5 min each, 4 °C), followed by 70%, 90%, 96% (2 × 5 min., RT), 100% (4 × 10 min, RT) and acetone (4 × 10 min., RT). Finally, the cells were incubated in an epoxy resin:acetone solution (1:1) overnight, RT, and the next day were deposited in epoxy resin blocks (Serva Electrophoresis, Heidelberg, Germany) and solidified at 60 °C for a week.

#### Post-embedding immunolocalization of MAGE-A1 and MAGE-C2 malignant melanoma-related proteins in ultrathin sections

The 70-nm-thick ultrathin sections were prepared with the usage of an ultra 45° diamond knife (Diatome, Nidau, Switzerland) installed on an ultramicrotome Power Tome XL (RMC, Tucson, USA); only straight and unbroken sections were mounted precisely on the matt surface of the 200 mesh nickel grids (Ted Pella, Redding, Ca, USA). Next, the grids with ultrathin sections face down were gently picked up with clean forceps and floated for 10 min onto the 0.02 M glycine (Biotechnology grade, BioShop Canada Inc., Burlington, Canada), made in PBS to neutralize free aldehyde groups. All steps were performed on a piece of clean Parafilm. Subsequently, the sections were treated 2 times for 5 min with the solution of PBS and triton (0.1%, reagent grade, Bioshop) to increase the permeability of the cell membranes. Triton was thereafter eluted 3 times with PBS. To prevent non-specific antigen-binding sites, the grids were placed for 1 h in a blocking solution (1% BSA in PBS, albumin fraction V, Carl Roth), which was rinsed with PBS 1 time (5 min). Next, for performing immunolocalization of the studied proteins, two types of primary antibodies diluted in 0.1% BSA in PBS: monoclonal rabbit anti-MAGE-A1 and monoclonal rabbit anti-MAGE-C2 (dilution 1:10) were applied onto the grids (1 h at RT). The following step was placing the grids 6 times at 5-min intervals on the droplets of PBS and distilled water, and gently whirling to remove the unbound antibodies. Afterward, the samples have been marked with the secondary goat anti-rabbit gold nanoparticle conjugated antibody (Abcam, Cambridge, UK, cat. no. ab27237, IgG H&L, 20 nm Gold, preabsorbed, Lot: GR3200298-7), prepared in 0.1% BSA in PBS (dilution 1:10) for 1 h at RT (dark chamber). The following step was washing the grids in PBS and distilled water 6 times for 5 min to mitigate background and non-specific binding of secondary antibodies to different cell sites.

To stabilize the ultrastructure of the cell membranes the sections were post-fixed in 1% glutaraldehyde (SERVA Electrophoresis) in PBS for 5 min; the fixative was then flushed with distilled water. To better assess the localization of the proteins at the ultrastructural level, the sections were post-stained with the uranyLess solution and Reynold's lead citrate 3% (Electron Microscopy Sciences, Hatfield, PA, USA) on top of the droplets for a few minutes, to augment the contrast of organelles membranes. During the next stage, surplus salts and any residues were washed off 3 times in distilled water in a low-form beaker. The last step was the evaluation of the grids in the TEM JEM-1011 microscope (Jeol, Tokyo, Japan). The acquisition of digital micrographs was performed using a TEM imaging platform iTEM1233 equipped with a Morada Camera (Olympus, Münster, Germany) at various magnifications. The documentation relies on the search for colloidal gold nanoparticles whose size ranges between 14 and 20 nm according to the manufacturer’s protocol.

### Holotomographic microscopy studies

Live holotomography was performed using a 3D Cell Explorer microscope (Nanolive SA, Ecublens, Switzerland). The cells were incubated and imaged using 35 mm Ibidi glass-bottom μ-Dish dishes (Ibidi GmbH, Germany). During the nsPEF procedure on the adherent cells, A375 cells’ photographs were captured. The temperature was set to 37 °C and controlled using an Ibidi Heating System (Ibidi GmbH, Germany), while a sufficient amount of CO_2_ was maintained by using a CO_2_-independent culture medium (Sigma-Aldrich).

### Electric field simulations

The spatial electric field distribution has been simulated in COMSOL Multiphysics (COMSOL, Stockholm, Sweden) software environment. A three-dimensional model of the cuvette electrodes (aluminum, 2 mm gap) and the tissue sample (L3 × H5 × W2/1.5 mm^3^) has been designed in the Electric currents (EC) physics module. The conductivity of the surrounding medium (phosphate-buffered saline (PBS)) of 1.5 S/m has been selected. The spatial electric field was simulated for tissue conductivity of 0.2 and 0.6 S/m. Also, two conditions were analyzed: (1) the sample is touching the electrodes and (2) freely floating in the suspension without direct contact with the electrodes. An extremely fine physics-controlled mesh, consisting of 936,109 finite elements and 44,368 boundary elements was generated. The 1.6 kV voltage has been used as an input.

### Ex-vivo tumor culture

In the study the total of 48 histopathology samples were prepared from the surgical excision of 14 primary and 34 metastatic melanoma tumors from Skin Cancer Unit Patients in the Lower Silesian Oncology Center. After surgical excision of the tumor, the tissue was placed in a DMEM-filled probe and transported to the laboratory. The tumor was cut into four, equal-size pieces and incubated for 1 h in the incubator to reach the cell culture conditions. Three tumor samples were carefully inserted into the electroporation cuvettes filled with electroporation buffer. The first sample was used to optimize pulse parameters and the second underwent electric field exposure. The third sample was a control. Finally, the samples were transferred gently to Petri dishes filled with DMEM and cultured for 24 h. First tumor samples were used to optimize the conditions of ex vivo culture to avoid a high level of cell death. At each stage of the study, the tumor was stained with Yo-Pro-1 to indicate dead permeable cells. Further, the samples were fixed with 4% formalin for 24 h and flooded in paraffine.

### Immunohistochemical reaction

Before the immunohistochemical reaction, the histological slides stained with hematoxylin and eosin were obtained from whole melanoma samples archived in the form of paraffin blocks. The paraffin blocks with melanoma samples were cut into 4 µm sections. The IHC reactions were performed using a recombinant Anti-MAGEA1 antibody (Abcam; cat. no. EPR4276) in a 1:250 dilution, recombinant Anti-MAGE3 antibody (Abcam, cat. no. EPR19065) in a 1:500 dilution, recombinant Anti-MAGEC2 antibody (Abcam, cat. no. EPR19064) in a 1:500 dilution. IHC reactions were performed using the DAKO Autostainer Link 48 (Dako). The visualization of the reactions was performed using EnVision™ FLEX High pH (Link) reagents (Dako). IHC reaction for MAGE-A1 and MAGE-3 antigens was assessed using the immunoreactive score (IRS) scale by Remmele and Stegner. This scale evaluates the percentage of positive cancer cells (A) and the staining intensity of the reaction (B). The result is the product of these two values (A x B). Following the antibody manufacturer's instructions, before carrying out the IHC experiments, we performed reactions to the positive and negative controls.

Nuclear expression of MAGE-C2 protein was determined using a scale that analyses the percentage of the number of cancer cells with positive nuclear expression of the antigens studied, according to the following scale: 0%, 0 p.; 1–10%, 1 p.; 11–25%, 2 p.; 26–50%, 3 p.; 51–100%, 4 p. All specimens were assessed using an OLYMPUS BX-41 light microscope (Olympus) by two independent pathologists. Controversial samples were consulted with the third independent pathologist.

### RNA sequencing analyses

Preprocessed single cell RNA-seq profiling (raw counts) of 31 patient-derived melanoma samples (7,186 cells in total) was downloaded from Gene Expression Omnibus (GEO) database, accession number: GSE115978^57^. Single cell RNA-seq profiling (raw counts) of 22 healthy human skin samples (~ 11,000 cells in total) that have been enriched for melanocytes was downloaded from (GEO) database, accession number: GSE151091^58^.

Preprocessed The Cancer Genome Atlas (TCGA) RNA-seq data (raw counts) of 89 primary Skin Cutaneous Melanoma (SKCM) tumors and 183 lymph node metastases were downloaded together with clinical data from GDC (Genomic Data Commons) database^59^.

Single cell RNA-seq data was transformed to Seurat object followed by filtering for cells containing a minimum of 200 and no more than 8000 unique genes and containing less than 10% mitochondrial genome. Filtered data was normalized using global-scaling normalization method “LogNormalize” (scale.factor = 10,000) in Seurat Bioconductor package (version 4.4.0)^60^.To visualize single cell RNA-seq data we selected the top 2,000 variable features identified using the vst method. Next data was scaled, and principal component analysis (PCA) was performed. Thereafter, the used elbow plot method to select number of principal components for Unifold Manifold Approximation and Projection (UMAP) non-linear dimensionality reduction followed by calculating the k-nearest neighbor graph and Louvain clustering . DimPlot(), FeaturePlot() and DotPlot() functions were used for visualizing gene(s) expression in low-dimensional space.

For bulk SCKM data lowly expressed genes were discarded and then were transformed by log(tpm) (transcript per million) values using in-house R script.

### Statistical analysis

**Figure 1:**
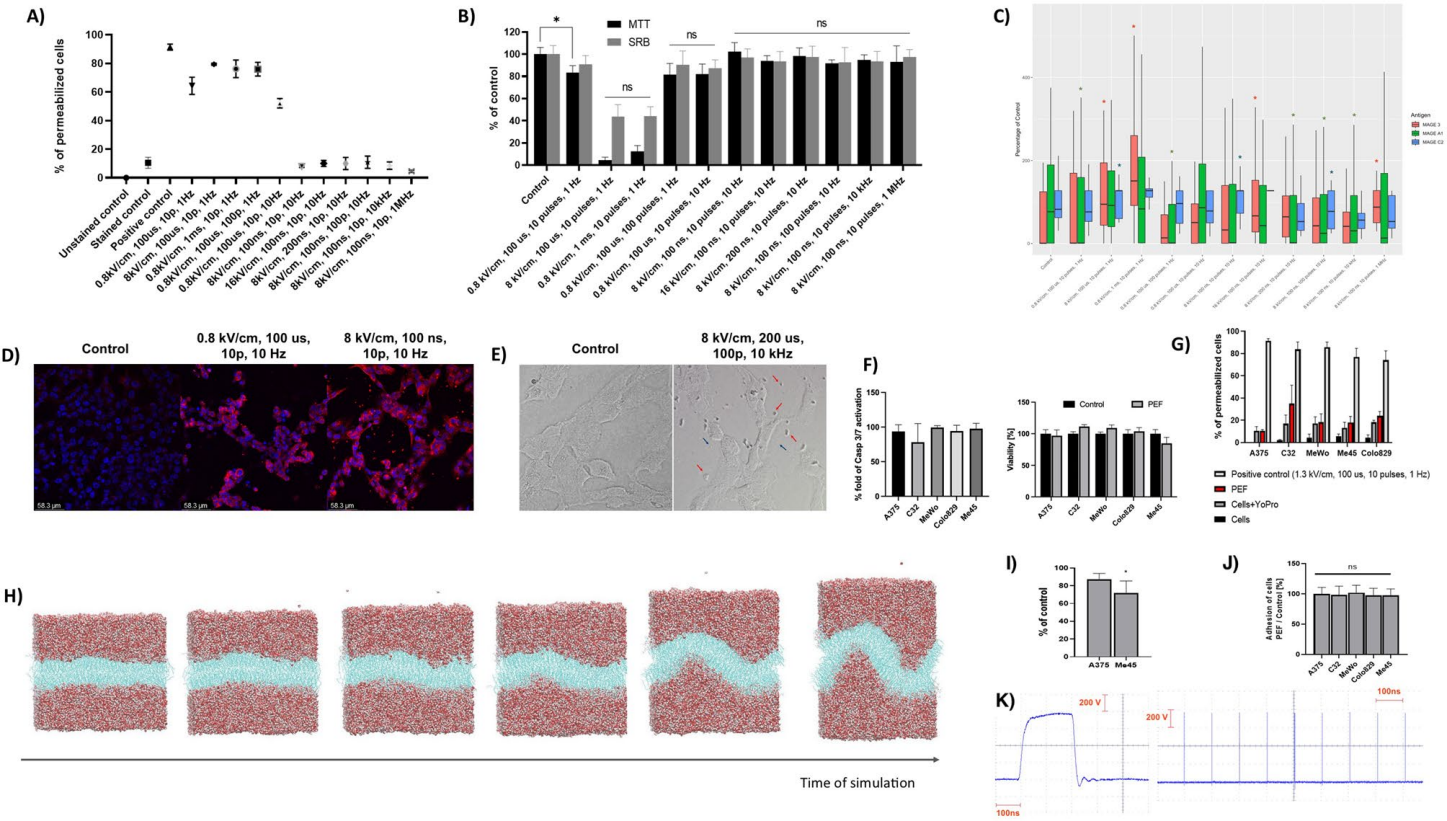
(**A**) Permeabilization of A375 cells to Yo-Pro-1 subjected to various parameters of PEFs. We increased each of four parameters (frequency, pulse duration, number of pulses, and voltage) and observed the effects on membrane permeabilization, positive control—ESOPE; (**B**) Viability of A375 cells 24 h after PEF exposure. The study involved the manipulation of pulse parameters, like frequency, pulse duration, number of pulses, and voltage. Both SRB and MTT assays were used to assess the viability of the cells. *p < 0.05, ns p > 0.05 in ANOVA test; (**C**) Fluorescence analysis of MAGE-A1, MAGE-3, and MAGE-C2 antigens on A375 cells exposed to various parameters of PEF and incubated 24 h afterward. Box plot shows the median value with 1st and 3rd quartiles, asterix above the boxes mean p < 0.05 in Kruskal–Wallis test; (**D**) Fluorescence microscopy photographs of A375 cells 24 h after exposure to PEFs of 0.8 kV/cm, 100 us, 10 pulses, 10 Hz and 8 kV/cm, 100 ns, 10 pulses, 10 Hz; (**E**) Super-resolution microscopy photograph of A375 melanoma cells subjected to the most optimal protocol of nsPEF exposure (8 kV/cm, 200 ns, 100 pulses, and 10 kHz). Red arrows indicate big in size vesicles and blue arrows indicate the small size vesicles; (**F**) MTT viability test of A375, C32, MeWo, Colo-829, and Me45 cells 24 h following nsPEF exposure (8 kV/cm, 200 ns, 100 pulses, and 10 kHz). (**G**) Flow cytometry study of permeability of the cell membranes of A375, C32, MeWo, Colo-829, and Me45 to YoPro-1 dye; H) Molecular dynamics simulation showing the membrane protrusions during nsPEF application; (**I**) Boyden’s chamber migration assay showing the reduced migration of Me45 cells through the collagen membrane when the cells were subjected to nsPEF; (**J**) Adhesive properties of cells subjected to nsPEF and life-stained using Vybrant assay; (**K**) Pulse parameters shown on graphs.

The laboratory experiments were performed in replicates. Data were expressed as mean ± SD (standard deviation) and analyzed by one-way and two-way ANOVA (in GraphPad Prism 8), with p < 0.05 being considered statistically significant (Fig. 1I,J). Kruskal–Wallis test was used to compare the control group with each of the electric field parameter groups. The non-parametric Wilcoxon signed-rank test for paired observations was used to compare groups of data from histopathological samples. Samples were assessed with IRS classification^61^.

Biostatistical analyses of the patients survival were performed in R 4.3.1 and Bioconductor 2.60. Continuous variables were assessed for distribution (normal, non-normal) using the Shapiro–Wilks test. Next, we assessed differences between groups using t-test (for normal distribution), nonparametric Kruskall–Wallis test (for non-normal distribution) and Chisquared test (for categorical variables). All reported p-values were corrected for multiple testing using the Benjamini and Hochberg method. Determination optimal cutpoint for MAGE genes expression values with respect to which cohort patients was stratified into two groups („high” expression and „low” expression) was done by using the maximally selected rank statistics^62^. To assess association of MAGE expression with overall survival (OS) we used multivariate analysis, using the Cox regression to adjust for potentially interacting covariates (age, gender and stage) in ‘survival’ and ‘survminer’ R packages^63^. Validity of the Cox model assumptions were assessed with cox.zph() function.

### Institutional review board

The study was conducted in accordance with the Declaration of Helsinki and approved by the Ethics Committee of Wroclaw Medical University (528/2021).

## Results

### Biophysical characterization of A375 cells subjected to nsPEF

In this section, we aim to optimize the nano-second Pulsed Electric Field (nsPEF) parameters to increase the expression of melanoma-associated antigens (MAGE-A1, MAGE-3, and MAGE-C2) in A375 melanoma cells. This involves assessing the cell membrane's permeability to Yo-Pro-1 dye and evaluating cell viability through MTT and SRB assays. Microsecond pulses of 0.8 kV/cm induce membrane permeability for Yo-Pro-1 (Fig. 1A). In high electric field (8 kV/cm) and long pulse duration (1 ms) there can be observed a high level of cell death (Fig. 1B). Based on our previous study—electro-exocytosis occurs when the cells are subjected to an electric field, which does not exceed the electroporation threshold^20^. Also, exocytosis after PEF exposure occurred in non-cytotoxic conditions. The release of membrane vesicles is also related to the modifications in the expression of membrane antigens. Based on the three requirements, we observed a high potential of “0.8 kV/cm, 10 Hz, 100 us, 10 pulses” and “8 kV/cm, 10 Hz, 100 ns, 10 pulses” protocols in enhancing the fluorescence of MAGE-A1 (Fig. 1C). Both protocols are not cytotoxic and do not induce full permeabilization to YoPro-1. Interestingly, small patch-like fluorescent structures were observed outside the cell only in the latter protocol (Fig. 1D). In the initial observation noted in Fig. 1C, we saw an increase in the fluorescence of MAGE-3 antigen correlating with a rise in the number of pulses from 10 to 100, and a similar trend when increasing the frequency from 10 Hz to 10 kHz. However, it is important to acknowledge that these findings do not demonstrate statistical significance and are based on a limited range of PEF parameters. Additionally, the response of MAGE-3 may not be representative of all antigens, as variable patterns of expression were noted. Therefore, while these preliminary observations suggest a potential trend, they should be interpreted with caution and verified through further, more comprehensive studies. A similar effect was observed when we increased the frequency of the pulses (from 10 Hz to 10 kHz). Based on the observed tendencies, we concluded that a (1) high number of pulses with (2) long duration and (3) frequency of their delivery may induce electro-exocytosis and molecular alternations in MAGE antigens’ content. Therefore, for further studies, we chose the “8 kV/cm, 10 kHz, 200 ns, and 100 pulses” protocol (pulses presented on Fig. 1K).

Next, we proved that the selected protocol induces the release of membrane vesicles from A375 cells under the super-resolution microscope (Fig. 1E). We extended our study to confirm the generalizability of the selected nsPEF protocol across various melanoma cell lines (C32, MeWo, Me45, and Colo-829). We confirmed that the chosen nsPEF exposure protocol induces similar changes among C32, MeWo, Me45, and Colo-829 melanoma cell lines. Namely, the protocol did not induce cytotoxicity in five melanoma cell lines (Fig. 1F) and does not induce permeabilization of their cell membrane (Fig. 1G). To visualize the effects of near to electroporation threshold nsPEF on the cell membrane morphology, we conducted a molecular dynamics study (Fig. 1H). A membrane composed of 10% cholesterol was simulated under non-electroporating conditions. The morphology of the cell membrane remained stable at the first picoseconds of the simulation, however, it remained enlarged at first, then the membrane started to curve. At this point of the study, we wanted to make sure that our nsPEF protocol won’t enhance cell motility or adhesion properties. We estimated the migration properties of A375 and Me45 cells migrating through the collagen chamber and observed no effect on A375 cells and a slight loss of migration properties in Me45 cells (Fig. 1I). On the other hand, we tested the adhesive properties of the cells after nsPEF exposure and observed no effects of the electric field on the adhesion of the cells (Fig. 1J).

### Electron microscopy studies

**Figure 2:**
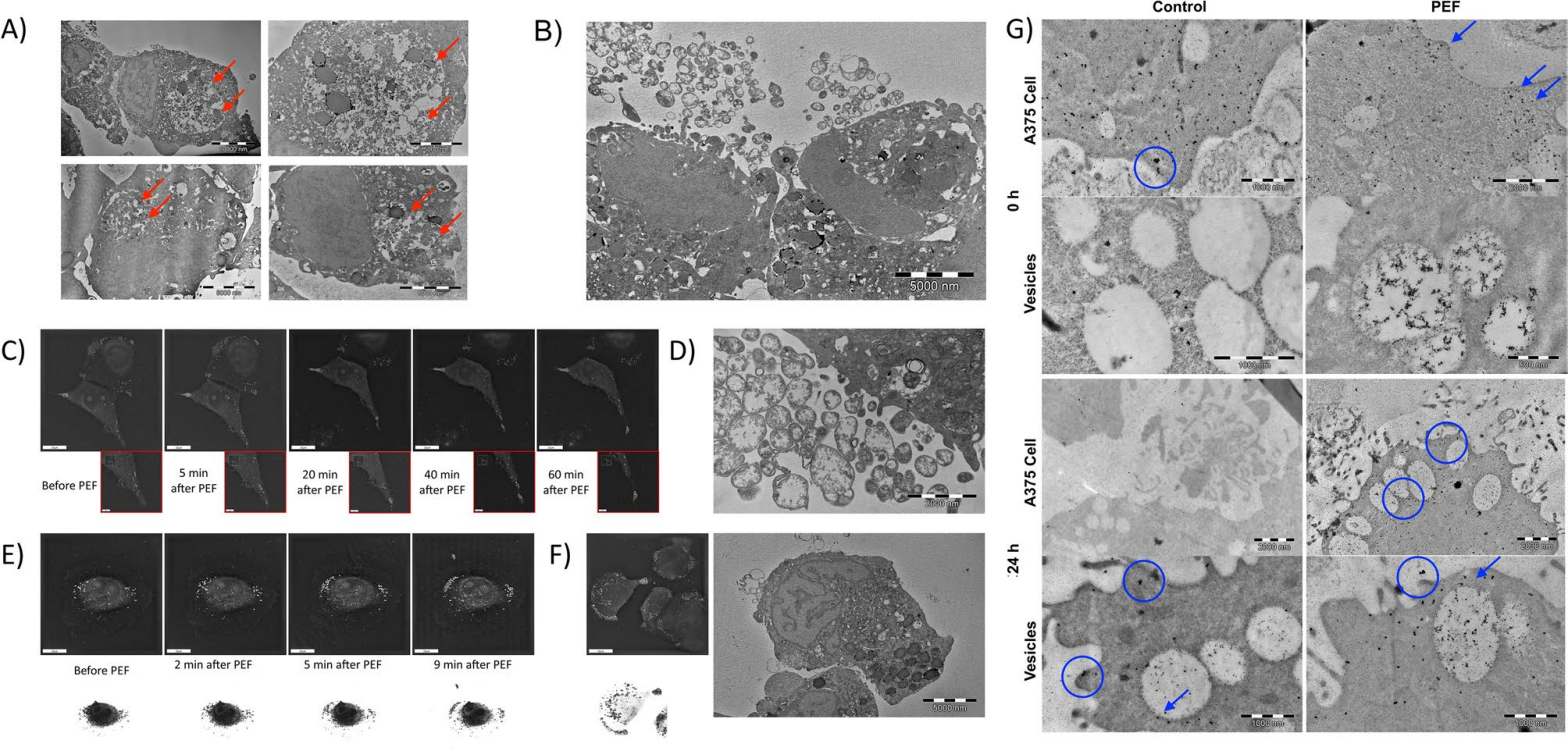
(**A**) Morphology of A375 cells observed with an electron microscope shows the high content of late autophagosomes and irregularly aligned early melanosomes (red arrows); (**B**) A375 cells secreting microvesicles after exposure of the cells with nsPEF. Membrane protrusions may be observed simultaneously with the loss of vesicles from the cytoplasm of the cells; (**C**) Holotomographic study analysis of the vesicles release from the cells exposed to nsPEF. After the nsPEF procedure, the A375 melanoma cell shifts the membranous intracellular structures (white ovals in the cytoplasm) to the periphery of the cell, and from then, cytoskeleton rearrangement moves the vesicles to the distal protrusions of the cell membrane. After several minutes, the membrane fragment is being released with the portion of vesicles; (**D**) Cell membrane of A375 cells subjected to nsPEF shows the extensive vesicles release; (**E**) Holotomographic microscopy study of the A375 cells’ response to the electric field exposure. After the procedure, the cell rearranges the vesicles to the periphery of the cell (white cytoplasmic vesicles); (**F**) A study using holotomographic microscopy and electron microscopy to observe A375 cells and the arrangement of membrane vesicles after nsPEF exposure. The results show the vesicles moving towards the outer edges of the cells; (**G**) A study using immunogold labeling to examine the MAGE-A1 antigen. The image captures the antigen's position before and 24 h following nsPEF treatment. Post-exposure, there is an accumulation of nucleic acids and proteins within the intracellular vesicles, which continue to be marked by osmium tetroxide, glutaraldehyde, uranium, and lead salts. Blue circles highlight the rearrangement of MAGE-A1 antigens close to the melanoma cells' membrane.

In this section, we investigate the impact of nano-second Pulsed Electric Fields (nsPEF) on the ultrastructure of A375 melanoma cells using electron and holotomographic microscopy. Our focus is on the morphological changes, vesicle secretion dynamics, and the relocation of MAGE-A1 antigens following nsPEF treatment. Electron microscopy was applied to examine the effects of nsPEF on the ultrastructure of A375 melanoma cells. The morphology of A375 cells observed with an electron microscope shows the high content of vesicles of different origin, phagosomes, late autophagolysosomes, and irregularly aligned early melanosomes (Fig. 2A). The cell presumably undergoes an extensive autophagy process. Conversely, Fig. 2B shows A375 melanoma cells secreting microvesicles 24 h after cells’ exposure to nsPEF. Membrane protrusions may be observed simultaneously with the loss of vesicles from the cytoplasm of the cells. Interestingly, the cytoplasm appears to be filled with vesicles at the same level of intensity in both control and experimental group cells. To analyze the secretion of the vesicles from melanoma cells, we performed holotomographic studies. After the nsPEF procedure, the A375 melanoma cell shifts the membranous intracellular structures (white ovals in the cytoplasm) to the periphery of the cell, and from then, cytoskeleton rearrangement moves the vesicles to the distal protrusions of the cell membrane (Fig. 2C). We can see the evolution of membrane protrusions, which eventually leads to the release of cytoplasmic content (i.e. phagosomes) to the extracellular space. During the 60-min observation, melanoma cells protrude cell membrane with the lipid-containing droplets far to the periphery of the cells. Enlarged photographs of the protrusions show the cytoskeleton fibers which transport the vesicles to the membrane extensions. After several minutes, the membrane fragment is released with the portion of vesicles. The observation is supported by Fig. 2D in which the cell membrane of A375 cells subjected to nsPEF shows extensive vesicle release. Holotomographic microscopy study of the A375 cells’ response to the electric field exposure. After the procedure, the cell rearranges the vesicles to the periphery of the cell—cytoplasmic vesicles in Fig. 2E. Besides, we observed the sustained relocation of membrane vesicles following nsPEF exposure using the holotomographic microscopy and electron microscope (Fig. 2F). The shift of the vesicles to the periphery of the cells was observed.

Immunogold labeling study of MAGE-A1 antigen was applied to examine the changes in MAGE-A1 expression following the application of nsPEF. The black dots pattern (encircled) in Fig. 2G shows the gold-labeled MAGE-A1. The photograph shows the localization of the antigen before nsPEF exposure and 24 h after the experiment. We may observe the overload of the intracellular vesicles with nucleic acid and proteins (remaining stained with osmium tetroxide, glutaraldehyde, uranium, and lead salts) following nsPEF exposure (Fig. 2G). Blue circles indicate the reorganization of MAGE-A1 antigens nearby the cell membrane of melanoma cells.

### MAGE antigens overview

**Figure 3:**
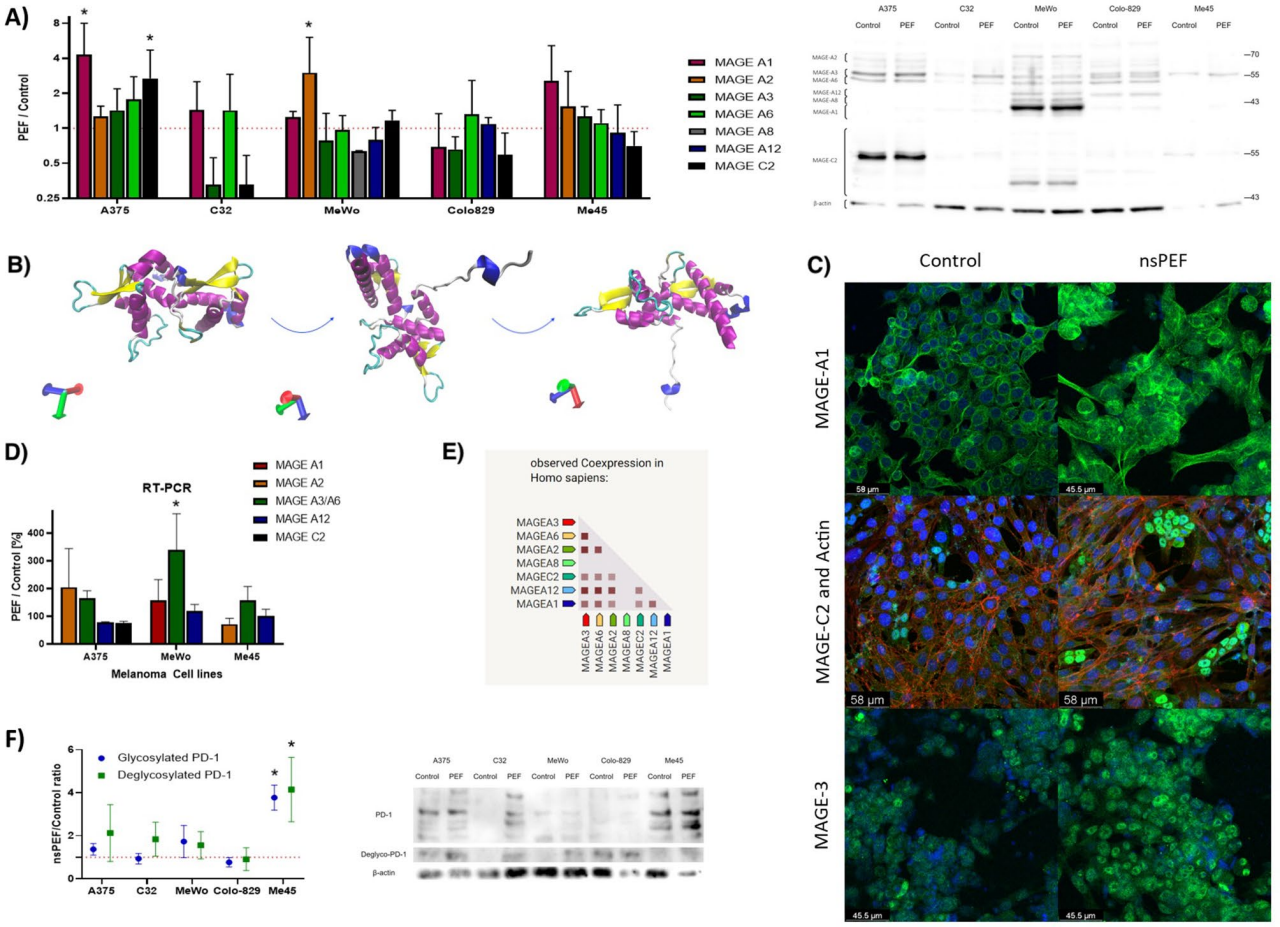
(**A**) Western Blot study of the MAGE antigens expression following nsPEF exposure, > 3 replicates, *p < 0.05 in ANOVA test; (**B**) Molecular structure of MAGE-A3 MHD (MAGE homology domain) (PDB: 4V0P); (**C**) Immunofluorescent photographs of A375 cells subjected to nsPEF and stained for MAGE-A1, MAGE-C2, and MAGE-3 antigens. MAGE-C2 stained cells were additionally incubated with phalloidin to visualize actin cytoskeleton; (**D**) RT-PCR studies of MAGE mRNA expression in cells subjected to nsPEF; (**E**) Coexpression of MAGE antigens show a clear link between MAGE-A2/3/6, *p < 0.05 in ANOVA test, (**F**) PD-1 in both glycosylated and deglycosylated forms following exposure to nsPEF, *p < 0.05 in ANOVA test.

In this section, we examine the effects of nano-second Pulsed Electric Fields (nsPEF) on the expression of MAGE A family proteins and PD-1 in melanoma cells. Our analysis includes Western blot, RT-PCR studies, and correlation assessments to understand the impact of nsPEF on these critical melanoma-specific antigens and immune checkpoint inhibitors. MAGE A family of proteins is melanoma-specific antigens’ group, which share structural similarities and the members of that group are expressed alongside each other and could be detected by the single antibody. Western blot analysis of the expression of the MAGE proteins is presented in Fig. 3A. The main feature observed during the study is the increased expression of MAGE A1 and MAGE A2. Importantly, cancer cells with initially high expression of certain MAGE antigen overexpressed it after treatment with nsPEF. Moreover, we observed the uneven expression pattern of different antigens among the analyzed melanoma cell lines. Noteworthy, low chemiluminescence signal contributes to the low reliability of the results. MAGE family members share structural similarities and structural motifs—the structure of MAGE protein could be observed in Fig. 3B. Figure 3C presents the expression and localization of MAGE antigens 24 h after exposure to nsPEF. MAGE A1 is highly overexpressed, particularly in the periphery of the cells. MAGE C2 on the other hand was expressed in only a group of small cells which gather in nest like structures. These kind of cells gatherings were not observed in the control sample. On the other hand, MAGE A3 expression pattern in PEF-treated cells shows the increased fluorescence from the periphery in comparison to more centrally distributed control sample. RT-PCR studies of melanoma cells subjected to PEF prove the increase in MAGE-A3/A6 mRNA in MeWo cells and to lower extent MAGE A1/A2 in A375 and Me45 cells (Fig. 3D). Combining both the increase in protein content and in its mRNA content we conclude that nsPEF induces the transcriptional and translational effect on MAGE family of proteins. Correlations between the expression of various members of MAGE-A group are presented in Fig. 3E. The graph shows the highest correlation between the antigens’ expression remains between MAGE A2, MAGE A3, MAGE A6, and MAGE A12. Across the whole analyzed material, MAGE A8 had the lowest correlation coefficient with other MAGE antigens. Interestingly, the second lowest co-expression occurs between MAGE A1 and MAGE A12 both in cell lines (correlation coefficient 0.39 for metastatic lines and 0.57 for primary tumor-derived cells) and tissue samples (0.55 in primary and 0.49 in metastatic tumors) analyzed with NGS.

Aside from melanoma-specific antigens, we assessed the expression of immune checkpoint protein PD-1 after subjecting the cells to nsPEF. PD-1 occurs in both glyco and deglycosylated forms, which differ in biological activity^64^. The increase in PD-1 expression both in glycosylated and deglycosylated forms was observed among cell lines, with high initial expression of the antigen (Fig. 3F).

### 3D cell culture model studies

This section of the manuscript explores the effects of 8 kV/cm nanosecond Pulsed Electric Fields (nsPEF) on melanoma cells cultured in 3D cell models. It focuses on assessing the structural changes and the localization of MAGE antigens post-exposure to nsPEF, using confocal microscopy techniques. The findings provide insights into the cellular mechanisms influenced by nsPEF in a 3D environment, which closely mimics in vivo conditions. The study model includes the cell-to-cell interactions and helps to understand the role and localization of MAGE antigens after exposure to PEF. For the study, we generated 3D cell culture models from A375, C32, MeWo, Colo-829, and Me45 cells using the rotor-based method. After the formation of 3D cell spheroids, we exposed one of them with nsPEF and compared it with the control.

**Figure 4:**
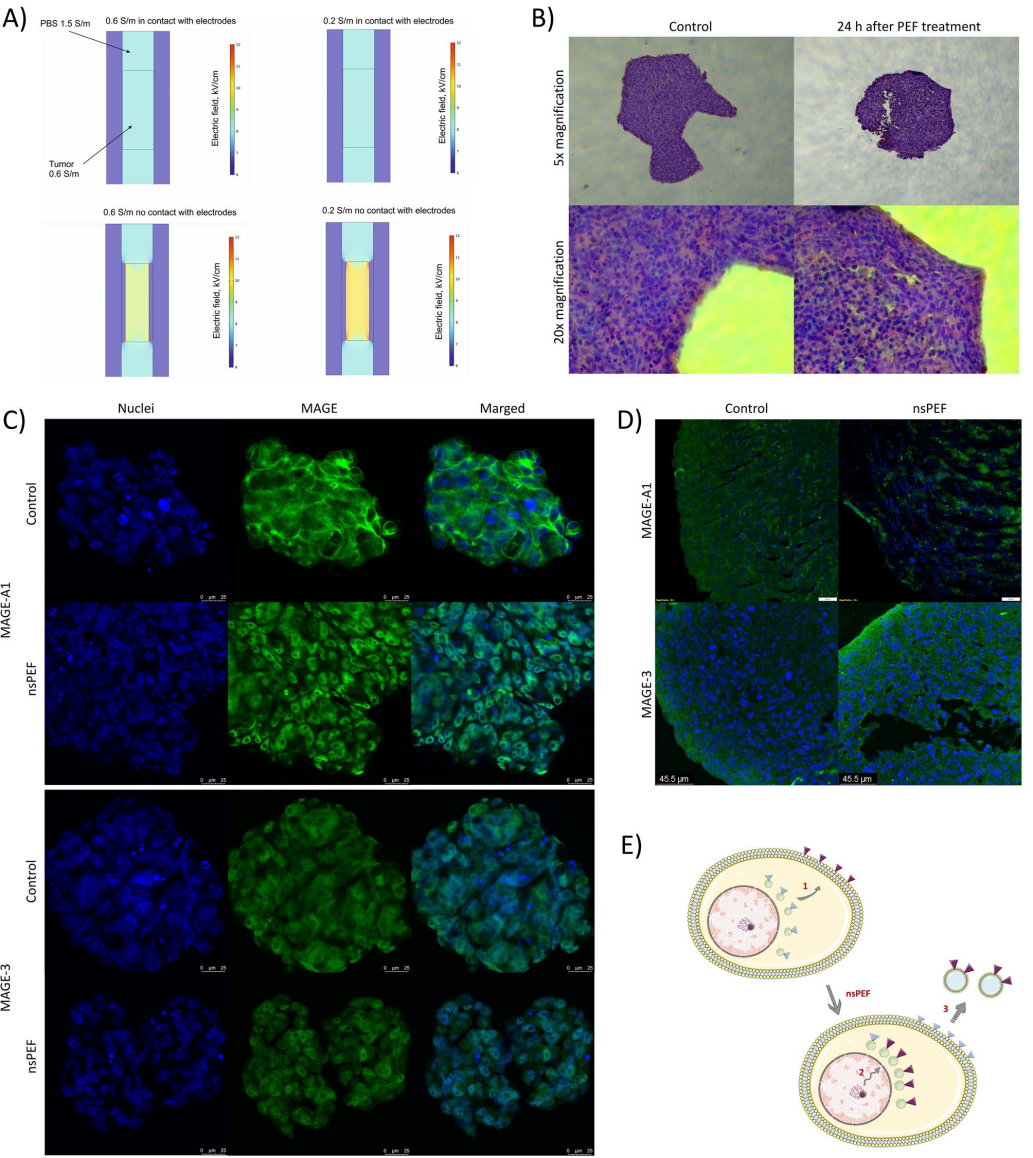
(**A**) COMSOL simulations of electric field distribution in both 0.2 and 0.6 S/m conductivity buffers. The bottom part of the figure shows the 0.6 S/m 3D spheroids in contact with the electrodes; (**B**) Morphology studies of MeWo cell line-derived spheroids stained with hematoxylin and eosin were exposed to 8 kV/cm, 200 ns, 10 kHz, 100 electric pulses under 5 and 20 magnification; (**C**) MAGE-A1 and MAGE-3 in A375 derived 3D spheroids stained with AlexaFluor-488™ 24 h after exposure to nsPEF. Photographs were taken in the Z-stack method; (**D**) MAGE-A1 and MAGE-3 antigen in a dissected slice of A375 derived 3D cell culture stained with AlexaFluor-488™ 24 h after exposure to nsPEF; (**E**) Scheme showing the mechanism of the influence of MAGE antigens localization after exposure to nsPEF: 1—a shift of the intracellular vesicles to the periphery of the cell, 2—the expression of MAGE antigens after their rearrangement, 3—release of membrane-localized MAGE antigens in microvesicles;

First, we calculated the most optimal technique to electroporate 3D tumors and tissues (Fig. 4A). Simulation of the electric field distribution in the electroporation cuvette shows that to achieve the constant electric field distribution in the 3D cell culture, the cell mass should be in touch with the metal plates of the cuvette. To assess the effects of electric field on the integrity and the morphology of the 3D melanoma (MeWo) cultures, we stained them with hematoxylin and eosin (Fig. 4B). The sphere subjected to nsPEF presents an increased rupture of the tissue in comparison to the control sample. Moreover, the edge of the sphere presents more densely packed cells in comparison to the control.

In next steps, the melanoma (A375) spheroids were stained for MAGE antigens and observed under the confocal microscope using the Z-stack method. Interestingly, in the spheroids, we observed the shift of the MAGE-A1 signal from the cell membrane to the cytoplasm and nuclei (A375 cells on Fig. 4C). Similar tendency was observed with MAGE-3 staining. Namely, the antigen’s signal shifted from the cytoplasm to the nuclei of the cells. The effect is better visible on the single green channel of the confocal microscopy images. Interestingly when the 3D tumor models were sliced using standard histology procedure, we observed the accumulation of the MAGE-A1 signal between the cells and next to the edge of the spheroid (Fig. 4D).

Based on in vitro single-cell studies and 3D cell culture models, we propose a mechanism in which melanoma cells initially release microvesicles containing MAGE-A1 localized in the cell membrane (step 2 on Fig. 4E). Simultaneously, intracellular vesicles containing MAGE-A3 shifts towards the periphery of the cell (step 1 on Fig. 4E). The initial loss of the antigens leads to the overexpression of membrane-localized MAGE antigens (step 3 on Fig. 4E).

### Metabolomics studies of cells after nsPEF exposure

**Figure 5:**
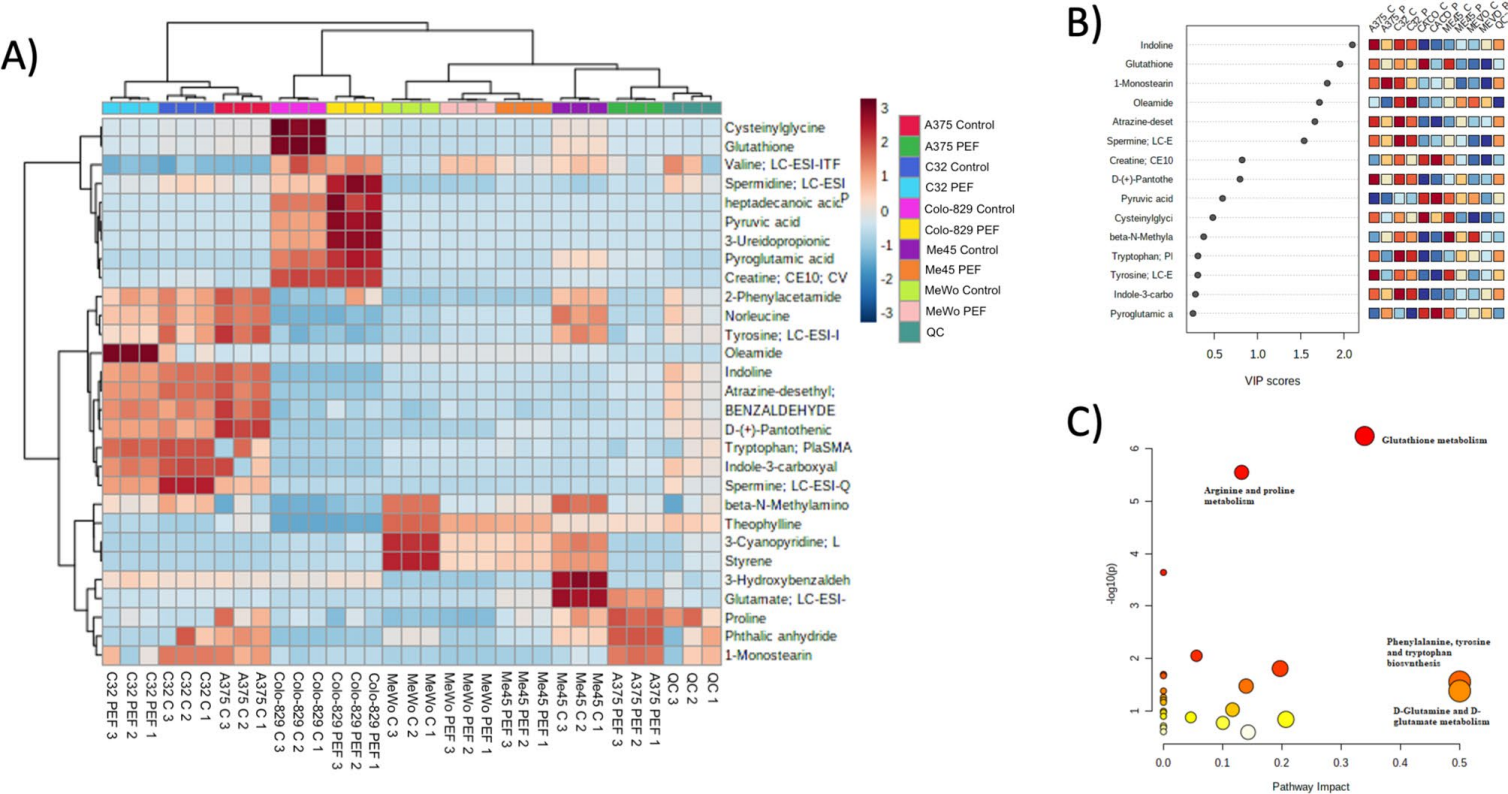
Clustering result shown as heatmap. (**A**) A375, C32, ME45, MeWo and Colo-829 cell lines. Distance measure using euclidean, and clustering algorithm using ward.D; (**B**) PLS-DA variable importance in projection (VIP) score derived from the hydrophilic/polar metabolite profiling data of A375, C32, ME45, MeWo, and Colo-829. The colored boxes on the right indicate the relative concentrations of the corresponding metabolite in each group under study; (**C**) Metabolic pathway analysis of selected metabolites. The metabolome view shows matched pathways arranged by p-values from pathway enrichment analysis (Y-axis) and pathway impact values from pathway topology analysis (X-axis). Node color and radius are based on the p-value and pathway impact value, respectively.

In this section, we utilize high-throughput untargeted metabolomics to analyze the effects of nanosecond electrical pulses on various melanoma cell lines. The focus is on identifying significant metabolic changes and clustering patterns among the cell lines, using advanced statistical methods like ANOVA, PCA, and PLS-DA. The results provide a detailed understanding of the metabolic pathways affected by nanosecond electrical pulses in these melanoma cells. Using high-throughput untargeted metabolomics, we compared A375, C32, Me-45, MeWo, and Colo-829 cell lines subjected to nanosecond electrical pulses and controls against each other. The analysis revealed 35 hydrophilic/polar identified metabolites. Univariate one-way ANOVA revealed 29 significant hits, with at least two of the ten groups being significantly different (false discovery rate (FDR)-corrected; p < 0.05). The hierarchical clustering analysis represented in the heatmap (Fig. 5A) illustrates clear sample clustering within each of the cell groups. Principal component analysis (PCA) revealed significant separation between the melanomas and Colo-829 cell lines as was to be thought, furthermore in the melanomas group A375 and C32 showed significantly different metabolic profiles, while Me45 and MeWo showed a similar metabolic profile overlapped with each other and partly with the A375 and C32 melanomas. Partial least squares-discriminant analysis (PLS-DA) intensified the clustering (Fig. 5B). Removal of Colo-829 from the analysis did not affect the separation of melanoma. The PLS-DA variable importance in projection (VIP) score, which ranks metabolites according to their importance for group separation, revealed 7 metabolites with a VIP-score > 1. However, considering the limitations of the research, the compounds with VIP scores below 1 were also loaded for the study of metabolic pathways. The following compounds were considered for the following analysis: Glutathione, 1-Monostearin, Oleamide, Spermine, Creatine, Pyruvic acid, Cysteinylglycine, Tryptophan, Tyrosine, Valine, Glutamate, Spermidine. Pathway analysis was conducted using Metaboanalyst 5.0 module. Three metabolomic pathways with impact score above 0.3 were revealed, namely: Glutathione metabolism (impact score = 0.34), Phenylalanine, tyrosine, and tryptophan biosynthesis (impact score = 0.5), D-Glutamine and D-glutamate metabolism (impact score = 0.5) (Fig. 5C).

### Lipidomic studies of cells after nsPEF exposure

**Figure 6:**
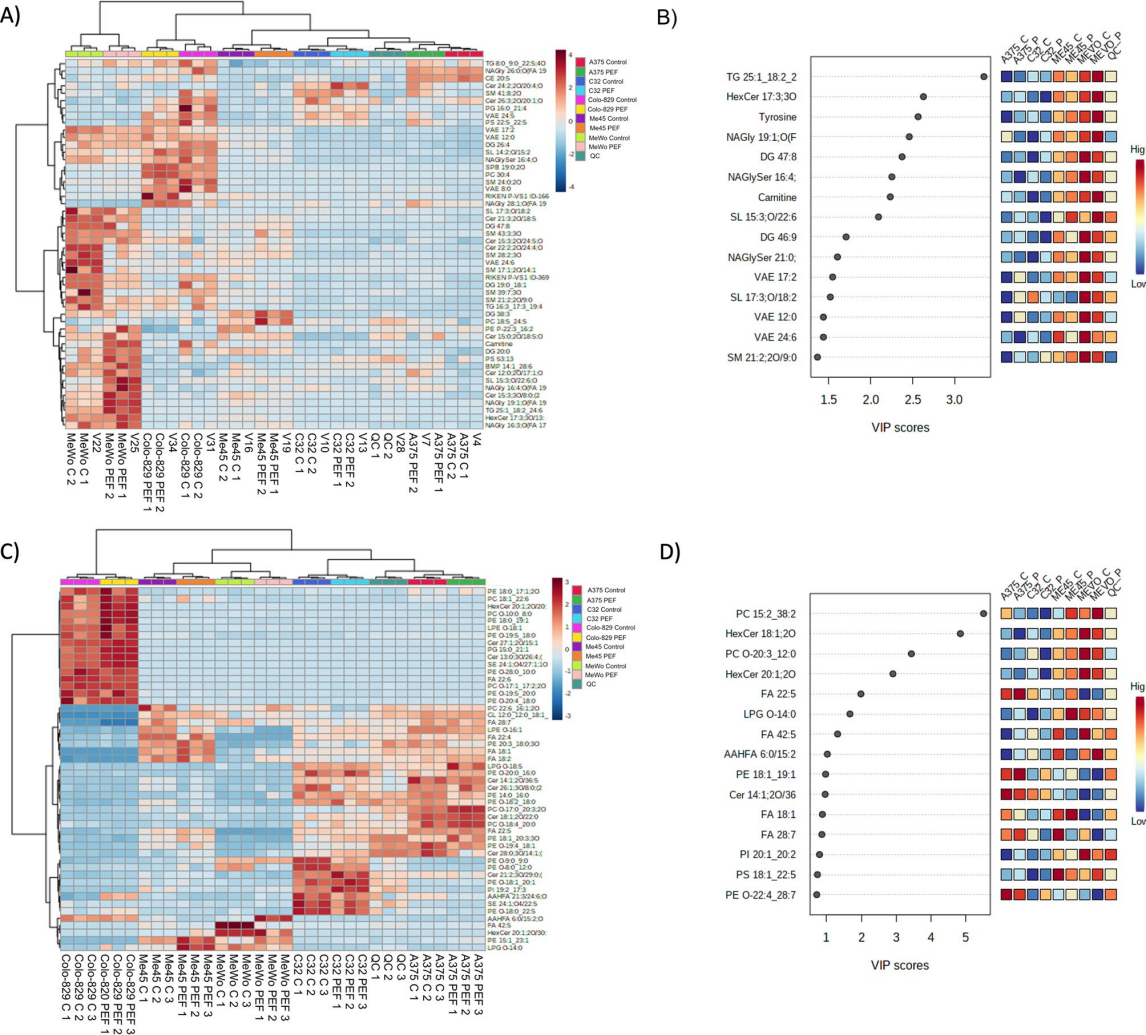
(**A**) Clustering result shown as a heatmap of lipids with a positive charge A375, C32, ME45, MeWo and Colo-829 cell lines; (**B**) Partial least squares-discriminant analysis (PLS-DA) scores of lipids with a positive charge A375, C32, ME45, MeWo, and Colo-829 cell lines; (**C**) Clustering result shown as a heatmap of lipids with a negative charge in A375, C32, ME45, MeWo cell lines. Distance measure using euclidean, and clustering algorithm using ward.D); (**D**) A375, C32, ME45, MeWo cell lines, and of lipids with a negative charge. Scores plot between the selected PCs. The explained variances are shown in brackets. PLS-DA variable importance in projection (VIP) score derived from the lipids profiling data of lipids with a positive charge and negative. The colored boxes on the right indicate the relative concentrations of the corresponding metabolite in each group under study.

This section presents a comprehensive lipidomics analysis conducted to profile the lipid species in various melanoma cell lines following treatment with nano-second Pulsed Electric Fields (nsPEF). The study aims to identify alterations in lipid composition and metabolism, thereby providing insights into the molecular mechanisms affected by nsPEF. The analysis encompasses both positive and negative ionization modes and employs advanced statistical methods to elucidate the impact of nsPEF on the lipidomic profiles of melanoma cells. Lipidomics analysis was conducted to comprehensively profile the lipid species in melanoma cell lines post-nsPEF treatment. This approach was aimed at identifying changes in lipid composition and metabolism, providing insights into the molecular mechanisms influenced by nsPEF and uncovering potential lipid-based targets for enhancing treatment strategies. Lipidomic untargeted analyses were conducted in positive and negative ionization modes, A375, C32, ME45, MeWo, and Colo-829 cell lines subjected to nanosecond electrical pulses, and controls were compared against each other. The analysis identified 2106 and 320 lipids and lipid-like metabolites in positive and negative ionization modes, respectively. For further analysis 100 lipid species with the highest intensity from each MS lipid analysis (positive/negative) were chosen. Positive charge lipids consisted of 14% SM (Sphingomyelin) species, 13% Cer (Ceramide) species, 12% NAG (N-Acylglycosphingolipid) species, 9% FA (Fatty Acid), 8% DG (Diacylglycerol) species, 7% VAE (Vinyl Acetate Ethylene), 7% TG (Triacylglycerol), 6% NAE (N-Acylethanolamine), 6% SL (Sphingolipid), 4% PC (Phosphatidylcholine), 3% PS (Phosphatidylserine), 2% PI (Phosphatidylinositol), 2% SPB (Sphingosine-1-phosphate), 1% each of PG (Phosphatidylglycerol), PE (Phosphatidylethanolamine), ST (Sterol), and BMP (Bis(monoacylglycero)phosphate). Negative charge lipids consisted of 36% PE (Phosphatidylethanolamine) species, 14% Cer (Ceramide) species, 14% FA (Fatty Acid) species, 11% PC (Phosphatidylcholine) species, 7% PG (Phosphatidylglycerol) species, 5% PI (Phosphatidylinositol), 4% PS (Phosphatidylserine), 2% SM (Sphingomyelin), 2% CL (Cardiolipin), 2% LPE (Lysophosphatidylethanolamine), and 2% LPG (Lysophosphatidylglycerol). One-way ANOVA resulted in 200 (all selected lipids) significant hits, with at least two of the four groups being significantly different (FDR-corrected; p-value < 0.05). The results of the hierarchical clustering analysis (heatmap) illustrate a good group separation between the four melanomas in both positive and negative groups and joined the group (positive Fig. 6A plus negative Fig. 6C). Principal component analysis (PCA) showed that Colo-829 is significantly separated from the melanoma cell lines groups (for both cases with a positive and negative charge). In contrast, PCA, as well as PLS-DA, showed also good group separation between the melanomas (Fig. 6B,D). In positive charge lipids species groups, A375, C32, Me45, and MeWo form clusters. In addition, the A375 and C32 melanoma groups overlap with each other and partially with Me45. Partial least squares-discriminant analysis (PLS-DA) intensified cluster formation. The PLS-DA variable importance in projection (VIP) score which ranks metabolites according to their importance for group separation revealed 15 metabolites with VIP > 1 score. The following compounds have VIP score above 1: TG (Triacylglycerol) 25:1_18:2_24:6, HexCer (Hexosylceramide) 17:3;3O/13:1;(2OH), DG (Diacylglycerol) 47:8, NAGly (N-Acylglycine) 19:1;O (FA 19:4), SL (Sphingolipid) 15:3;O/22:6;O, NAGlySer (N-Acylglycine serine) 16:4;O, Carnitine, Tyrosine, VAE (Vinyl Acetate Ethylene) 17:2, VAE 24:6, SL 17:3;O/18:2, Cer (Ceramide) 22:2;2O/24:4;O, NAGly 16:3;O (FA 17:4), SM (Sphingomyelin) 21:2;2O/9:0, SM 17:1;2O/14:1, VAE 12:0, Cer 21:3;2O/18:5, SM 28:2;3O, NAGlySer 21:0;O, DG 19:0_18:1. Namely, TG 25:1_18:2_24:6, SL 15:3;O/22:6;O, NAGly 19:1;O (FA 19:4), HexCer 17:3;3O/13:1;(2OH), Carnitine, Tyrosine, NAGly 16:3;O (FA 17:4), increased in MeWo cells stimulated with nsPEF. Conversely, DG 47:8, NAGlySer 16:4;O, VAE 17:2, VAE 24:6, SL 17:3;O/18:2, Cer 22:2;2O/24:4;O, SM 21:2;2O/9:0, SM 17:1;2O/14:1, VAE 12:0 (in Colo-829 as well), Cer 21:3;2O/18:5, SM 28:2;3O (both opposedly to Me45), DG 19:0_18:1 decreases after stimulation with nsPEF.

In negative charge lipids species groups, in PCA analysis Me45 and MeWo are significantly separated from the other melanomas. In addition, the A375 and C32 melanoma groups overlap with each other. Partial least squares-discriminant analysis (PLS-DA) intensified cluster formation for all melanoma groups. The PLS-DA variable importance in projection (VIP) score revealed the following 10 metabolites with VIP > 1 score: PC (Phosphatidylcholine) 15:2_38:2, HexCer (Hexosylceramide) 18:1;2O/34:3, PC O-20:3_12:0;2O, HexCer 20:1;2O/30:1;O, FA (Fatty Acid) 22:5, LPG (Lysophosphatidylglycerol) O-14:0, FA 42:5, AAHFA (Acyl-alkyl-hydroxy fatty acid) 6:0/15:2;O, PE (Phosphatidylethanolamine) 18:1_19:1, Cer (Ceramide) 14:1;2O/36:5. Using high-throughput untargeted metabolomics, we compared A375, C32, Me45, MeWo, and Colo-829 cell lines subjected to nanosecond electrical pulses and controls. The largest differences in lipidome between the group subjected to nanosecond electrical pulses and controls were found for the Me45 and MeWo cell lines, however, it should be mentioned that the differences were caused mainly by negatively charged lipids species. PC 15:2_38:2, FA 22:5, increased in both Me45 and MeWo cell lines. Conversely, FA 42:5 decreased in Me45 and MeWo cell lines.

### MAGE antigens expression in healthy skin and melanoma tumor

This section details the ex vivo studies conducted on publicly available single-cell sequencing data and bulk tumor RNA-seq. In addition, we assessed MAGE expression in patient-derived melanoma tumors subjected to nano-second Pulsed Electric Fields (nsPEF).

**Figure 7:**
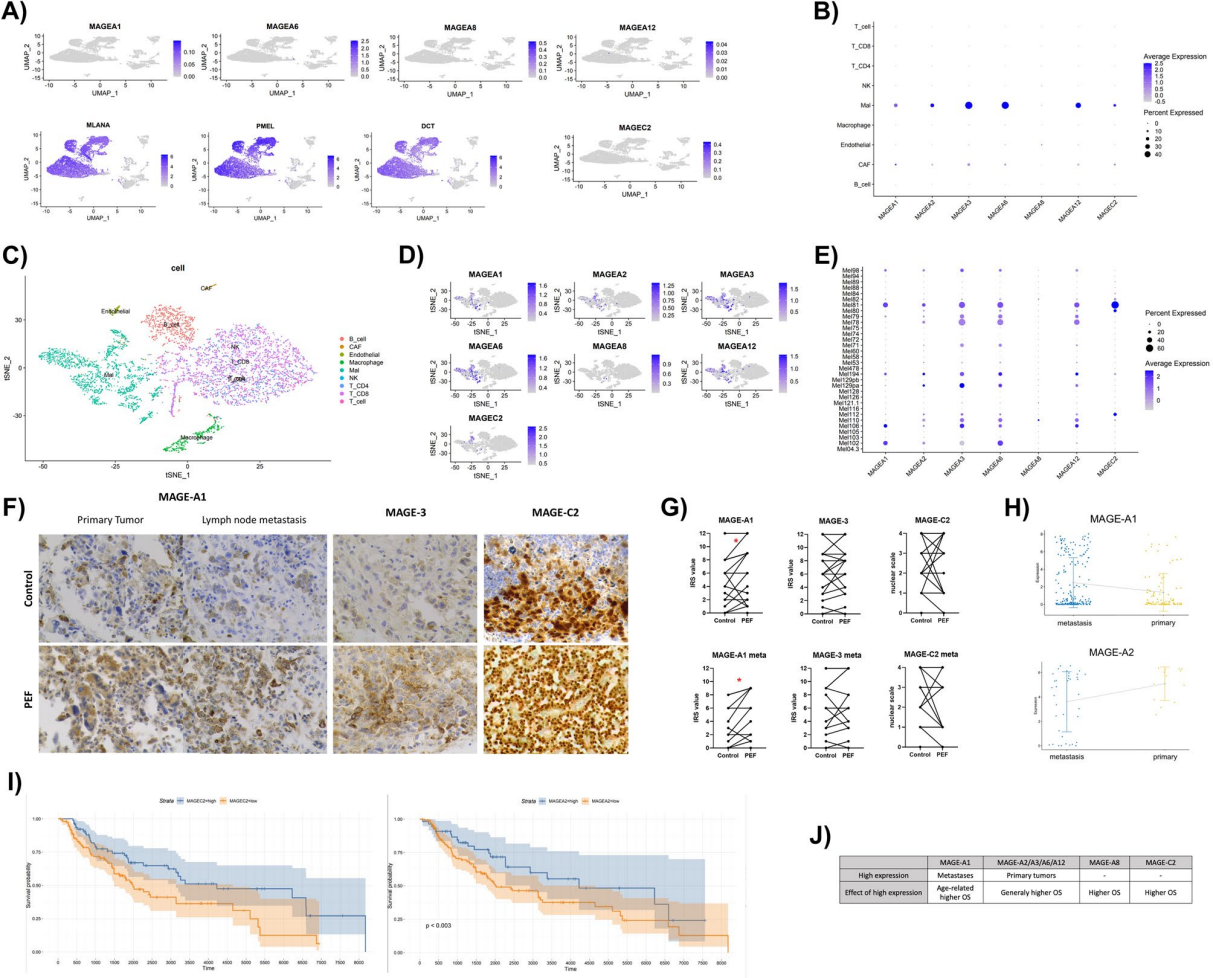
MAGE antigens expression in healthy skin and melanoma tumors: (**A**) UMAP plot showing MAGE antigens expression in melanocyte enriched skin samples from healthy individuals (GSE151091). Melanocytes are indicated by expression of MLANA, PMEL, and DCT; (**B**) DotPlot showing expression of MAGE-A1, MAGE-A3, MAGE-A6, MAGE-A8, MAGE-A12, MAGE-C2 in melanoma in various tumor cell populations. Dot radius represents percent of tumors expression given gene. Color intensity of dots reflects level of expression of given gene; (**C**) UMAP plot showing the result of clustering of expression profiles of 7,186 cells (GSE115978); (**D**) UMAP plots that directly corresponds to UMAP plot shown in (**B**) with overlayed expression of MAGE antigens. One can observe specificity of MAGE expression to melanoma tumor cells; (**E**) DotPlot showing variability of MAGE expression with respect to individuals. Dot radius represents percent of tumors expression given gene. Color intensity of dots reflects level of expression of given gene.; (**F**) Immunohistochemical reaction for MAGE-A1, MAGE-3 and MAGE-C2 antigens on primary and metastatic melanoma tumors derived from melanoma patients further exposed ex vivo to nsPEF; (**G**) IRS scoring system for the IHC assessment of MAGE-A1, MAGE-A3 and MAGE-C2 expression in 24 patient-derived primary and metastatic tumor cultures subjected to nsPEF, * p < 0.05 in non-parametric the Wilcoxon signed-rank test for paired observations; (**H**) Plots showing expression differences in MAGE-A1 and MAGE-A2 between primary tumors and lymph nodes metastases; (**I**) Kaplan–Meier estimates of survival probability for lymph nodes metastases depending on days from cancer diagnostics grouped by optimal cutpoint of MAGE-A2 and MAGE-C2 expression levels.; (**J**) Summary of the relationship between each analyzed MAGE antigen member and the clinical characteristics.

First, we examined whether the expression of MAGE can be observed in melanocytes from healthy individuals by re-analysing of single cell RNA-seq study (GSE151091) that included 22 normal skin samples (~ 11,000 cells in total) that have been enriched for melanocytes. By using common melanocyte markers (MLANA, PMEL, and DCT) we indentified localization of malanocytes on UMAP (Fig. 7A). The expression of selected MAGE antigens could not be detected in melanocytes as well as other cell types from healthy skin (Fig. 7A). Next, we examined expression patterns of MAGE antigens in various cell types (immune cells, cancer-associated fibroblasts (CAFs) and melanoma cells) within the tumor by re-analysing of single cell RNA-seq study (GSE115978). Furthermore, the single cell RNA-seq study allowed us to explore the heterogeneity in MAGE antigen expression among patients. Figure 7B–D presents the expression of MAGE A1, MAGE A3, MAGE A6, MAGE A8, MAGE A12, and MAGE C2 antigens in melanoma tumors. All MAGE expression was specific to melanoma cells. A faint signals of MAGE expression was also observed on cancer-associated fibroblasts (CAF). MAGE A8 expression could not detected in melanoma cells. We observed high variability of different melanoma tumors with respect to level of expression of MAGE (Fig. 7E). Most of the patients expressed MAGE-A3 and MAGE-A6 antigens, with co-expression of some other MAGE group members. Notably, some patients did not displayed expression of any of the mRNA for the MAGE antigens in their tumors.

After analyzing the tumor microenvironment and the expression pattern of MAGE antigens, we moved to immunohistochemical studies of MAGE-A1, MAGE-3, and MAGE-C2 expression on melanoma tumors subjected to nsPEF. Figure 7F shows the representative microscopy images of the tissues with melanoma cells that express MAGE antigens in neoplastic cells. The tendencies observed in cell line studies were also observed in ex vivo tissues. Wilcoxon signed-rank test for paired observations showed a statistically significant increase in MAGE-A1 expression (p = 0.0449) in all melanoma samples (Fig. 7G). Curiously, there was no statistically significant difference in primary melanoma tumors (p = 0.7813), but there was a high difference in MAGE-A1 expression in metastatic melanoma tumors (p = 0.0254, Fig. 7H).

To assess if the expression of specific MAGEs may depend on the metastatic status of melanoma tumors, we performed re-analysis on TCGA-derived 89 primary SKCM tumors and 183 lymph node metastases. We detected statistically significantly higher expression (p-value < 0.003) of MAGE-A2 in metastatic tumors in comparison to primary tumors (Fig. 7I). No other MAGE antigens have shown significant difference in expression between primary and metastatic tumors. Notably, we confirm observation from single cell RNA-seq analysis: MAGE-A8 was lowly expressed in melanomas (primary and metastatic) (Fig. 7I).

Moreover, aside from the lack of statistical significance, there might be observed a stable decrease in the expression of highly co-expressed MAGE A3, A6, and A12 in metastatic tumors. In this case, expression of MAGE A1, A8, and C2 was stable and there were observed no changes between primary and metastatic cells. Curiously, the situation changes when tissue samples were analyzed (Fig. 7I). Namely, the expression of associated MAGE A3, A6, and A12 antigens was not increased in primary tumor samples. Moreover, MAGE A1 was the only antigen to decrease Its expression in primary tumor samples. Here we observed no changes across MAGE C2 and MAGE A8. All the data is summarized in Fig. 7K.

Next, we aimed to assess the association of the level of each of the MAGE antigens’ expression with the survival of the melanoma patients. In primary tumors we found no significant association between MAGE expression and OS of patients (data not shown). In lymph node metastatic tumors we identified age variable as strong confounder therefore all reported results were adjusted to age. We found high expression of MAGE-A2 (p-value = 0.02) and MAGE-C2 (p-value = 0.019) to be independently (of age) associated with longer OS (Fig. 7J).

## Discussion

Electric field exposure may modulate the expression of various antigens^65^, thus we examined the expression of melanoma-specific antigens from the MAGE group and PD-1 molecule. After a systematic exploration of different voltage levels, pulse durations, pulse counts, and pulse delivery frequencies, we have identified 8 kV/cm, 200 ns, and 100 pulses at 10 kHz frequency as preliminary parameters that appear to influence the modulation of cells’ immunophenotype. Interestingly, these parameters of electric field induced the release of MVs and those were the same parameters as the ones used for obtaining MVs from EPP85-181RDB cells in our previous study^20^. The release of MVs is supported by the molecular dynamics study in which we can observe the increased curvature of the membrane of low-surface tension when subjected to EF. The simulation shows that the electric field is not susceptible to forming a pore under electric field conditions, but rather the membrane shows resistance and becomes curved to avoid portion. Similar tendencies were derived by us in the previous study, which showed the relation between the electroporation threshold and the surface tension of the membrane^66^. Namely, the lower the surface tension, the greater the electric field required to form a pore in the membrane. Here rises a question about the extracellular and not intracellular direction of the MVs release. Based on the physics of the process characterized in our previous study of the relation between surface tension of the membrane and the resistance to EF^66^, we conclude that the actin fibers allow for the greater resistance of the inside of the cell to accommodate any sheer stress, thus the vesicle is released to the extracellular space.

In compliance with the MISEV2018 guidelines established by the International Society for Extracellular Vesicles (ISEV), our study has classified the structures being released from the cells as microvesicles (MVs). Of particular note, our study utilized Nanolive imaging technology to closely observe these MVs. This technique has enabled us to directly witness the release of microvesicles from the cells, providing valuable live insights into their behavior and characteristics. In a significant advancement, we have further validated the origin of these microvesicles as deriving from cancer cells. This was achieved through the innovative application of the immunogold staining method, specifically targeting MAGE antigens found on the membrane of melanoma cells. This methodological breakthrough not only confirms the cancerous origin of the microvesicles but also enhances our understanding of their pathological significance.

Our previous study concerning the effects of nsPEF on pancreatic cancer cells confirmed that the application of an electric field induced the release of microvesicles from the cell membrane^20^. When we treat low-in-cholesterol membrane patches of low-surface tension with a sub-electroporation threshold electric field, the membrane protrudes. Our other study showed the role of actin fibers in the regulation of membrane surface tension and its role in resistance to the electric field^66^. Combining all together, we can see the tendency to release membrane vesicles from cells subjected to nsPEF. To optimize the protocol of PEF treatment, we evaluated (1) viability, (2) permeability and (3) fluorescence of MAGE antigens upon treatment with various electric fields. We clearly see that the protocol (200 ns, 100 pulses, 8 kV/cm, 10 kHz), which previously led to the release of MVs does not induce A375 cells permeabilization nor their direct death. While the protocol generally leads to an increase in MAGE signal, significant variability is observed among individual cells. Despite this heterogeneity, there appears to be a trend of similar cellular responses across different cell lines. However, considering the modulatory effects documented in our related studies^14,20^, and acknowledging the variability in antigen display, we propose that our findings might contribute to a broader strategy for modulating the immunophenotype of cancer cells, rather than asserting the discovery of a universally applicable protocol^14,20^. Adding to that information from the lipidomic studies we can obtain greater insight into aggressiveness of melanoma cells which is closely related to the lipid composition of cancer cell membrane^67^. Our lipidomic data shows the increase in triacyloglycerol (TG), N-acetylglycerol (NAGly) and hexyloceramide (HexCer) content in melanoma cells after subjecting them to nsPEF. High ceramides content mediates apoptotic process. Due to the opposed regulation of sphingomyelin and ceramide levels via sphingomyelinase, we may expect the reduced tumor promotion and reduced negative regulation of autophagy^68,69^. Besides, low triacylglycerols content is characteristic for cancer tissues and our PEF method allows for its increased level and thus presumably the reduced lipid metabolism in the cancer cells^70^. On the other hand, cancer cells reduced the level of diacylglycerol (DG), N-acetylglycineserine (NAGlySer), Vitamin A ester (VAE), ceramide (CE) and sphingomyelin (SM) in their cell membranes. The result support the idea of the increased vulnerability of cells subjected to nsPEF by the elevated level of HexCer and the reduced SM content^67^. Vitamin A esterification is prevalent in malignant melanocytes whereas benign melanocytes lack the ability to esterase vitamin A^71^, thus low level of VAE may be associated with benign phenotype of the cells. Moreover, reduction of DG level as a signaling molecule, results in the reduced tumor surveillance^72^.

Studies of the focal therapies proved the dualistic effects of the therapies on cancer cells. On one hand they may enhance the expression of cancer associated molecules and on the other they highly increase the expression of immune checkpoint inhibitors^13–15,20,73^. Moreover, study by Sauer et al. proved that nsPEF modulates the secretion of cytokines produced by melanoma cells^14^. Therefore, in this study we aimed to evaluate the functional properties of the cells after subjecting them to nsPEF. The adhesion, migration and proliferation were not elevated in any of the cell line, which contributes to the safety of the potential. Interestingly, the Me45 cell line reduced the migration potential through the Boyden chamber. Metabolomic studies further allowed for the assessment of cancer metabolism and function after nsPEF treatment. Crucial findings involved the lack of GSH, which may show the inhibitory role of pulsed electric field on tumor potential. Excessive GSH promotes tumor progression, where elevated levels correlate with increased metastasis as proved by Bansal et al.^74^. Our analyzed cell lysates showed lower indole levels, which is characteristic of non-cancerous tissues. Tyrosine, Phenylalanine, and Tryptophan concentration are increased in cancer tissue^75^. Moreover, low expression of tyrosine, phenylalanine and tryptophan degrading enzymes correlates with poorer survival in patients with HCC^76^. Interestingly, melanoma samples subjected to nsPEF contained higher pyroglutamine acid content, which also suggests excessive glutamic acid or glutamine in cancer cells exposed to nsPEF. Melanoma cells often experience low glutamine levels, which promote cell dedifferentiation^77^. Moreover, high glutamine diet significantly decreases tumor growth and decreases the expression of melanoma-associated oncogenes^78^. Within the diverse population of breast cancer cells, subsets exist that primarily metabolize glutamate via transaminases, enzymes whose expression levels are typically low in non-proliferative (quiescent) cells. Conversely, as these cells transition from a quiescent state to a more active, proliferative state, there is a notable induction in the expression of glutamate dehydrogenase (GLUD). GLUD plays a pivotal role in glutamate catabolism, particularly in actively proliferating cells, reflecting the metabolic shifts that support increased energy and biosynthetic demands^79^. All metabolomic and lipidomic studies suggest the reduced aggressiveness of melanoma upon exposure to nsPEF.

Our molecular biology studies showed that the release of MVs and the changes in MAGE antigens’ localization, led to the enhanced transcription of MAGE mRNA and then to the overexpression of the antigens. The expression pattern was different among different cell lines. We may see the steadily pattern of overexpressing the MAGEs that are initially highly expressed on cancer cells. Therefore, before the potential application of MAGE in melanoma therapy there has to be done an histopathological examination of antigens pattern. Interestingly, we observed changes not only in the expression of MAGE antigens but also in the PD-1 molecule, which expression increased after exposure to nsPEF. All in all, when the antigen is initially highly expressed, PEF induces its overexpression, thus the modulatory effects refers only to the proteins with high baseline expression. To further analyze the effects of nsPEF on the immunophenotype of melanoma cells, we applied electron microscopy. We observed that the excreted vesicles contain proteins, specifically the ones from MAGE family. Our immunogold staining studies proved the presence of MAGE A1 antigen in the membrane vesicles. We also observed the antigens on the cell membrane, which were released from the cell after stimulation with PEF. Curiously, most studies concerning the role of vesicles after PEF exposure focus on endocytic vesicles. Especially interesting are studies about the role of endocytosis in the electroporation-induced transport of genetic material to the cells^80–82^. The only studies concerning the electroporation-induced release of vesicles concern the joining of the cells with the intracellular vesicles and thus the release of chromogranin A and other excretory proteins^82,83^. To further investigate the MAGE antigens localization in the tumor-like environment, we applied confocal microscopy of 3D melanoma cell culture models. We observed the altered localization of MAGE A1, which was mainly expressed on the cell membrane in control cells and moved to the cytoplasm of the cells upon treatment with nsPEF. Typically, it has been noted that antigens originally situated in the cell membrane tend to migrate out of the cell via microvesicles, while those initially found in the cytoplasm and nucleus tend to relocate to the cell's periphery and membrane. This observation is crucial when moving from the cellular effects of nsPEF to the tumor related effects of nsPEF. Next stage of our study—the pathology examination proved that the expression of MAGE antigens is mainly restricted to melanoma, making them a potent target for anticancer therapy. We observe high-level variety in the expression of various forms of the antigen in different samples from melanoma patients, thus the potential therapeutical utility of the antigens should focus on the usage of antibodies that are not restricted to any specific form. The immunohistochemical reaction on melanoma tumors proved the enhanced expression of MAGE-A1 antigens after nsPEF exposure of cancer-derived tissues. Curiously, we also proved that the enhanced expression of the antigen correlates with the favorable outcome for the patients.

Although the cells differ in MAGEs expression, the epitopes from MAGE-A, MAGE-B and MAGE-C general groups are recognized by the same antibodies, thus the targeting should focus on the whole subgroup of MAGEs, rather than the exact antigen itself. The interplay between MAGE and PD-1 expression shows the complexity of the problem of membrane antigens’ modulation. Increased expression of both molecules would presumably lead to the increased recognition of the tumor, but also enhanced immune blockade mechanisms in the tumor microenvironment^13,84–86^. The problem may hopefully be overcome by the utilization of anti-PD-1 antibodies combined with anti-MAGE cell-based therapy. Besides, our study provides valuable insights into the upregulation of PD-1 in melanoma cells following nsPEF exposure, highlighting a potential avenue for modulating immune checkpoint inhibitors in cancer therapy. However, we recognize the limitation that these findings, observed in melanoma cell lines, may not directly translate to general mechanisms in immune cells, particularly T cells. While the upregulation of PD-1 in melanoma cells is an intriguing finding, it is essential to emphasize that its regulation in immune cells, specifically T cells, requires further experimental validation. This distinction is crucial, as immune cells and cancer cells may exhibit divergent regulatory mechanisms^26,87,88^.

Up to this day, various clinical trials aim to combine the immune checkpoint inhibitors targeting with cancer associated antigens targeting in the same bispecific antibody^89^. All in all, our studies showed both in vitro and ex vivo the potential of nsPEF to increase the expression of melanoma-specific molecules. The mechanism involves the shift in antigens’ localization and the increased transcription of mRNA and translation into MAGE antigens. Curiously, the increase in membrane antigens is not specific to MAGE antigens, but also PD-1 molecule enhances as well. Our study shows a novel technique to modulate membrane antigens of cancer cells, but still, much research has to be done in this field.

## Conclusions

Exposure of melanoma cells to nsPEF stimulates electric field-induced exocytosis of MAGE-A1 antigen-containing microvesicles. Initial lack of the antigen on the cell membrane upon exposure to nsPEF induces the transcription of MAGE mRNA and overexpression of the MAGE-A1 gene on the cell membrane of melanoma. Moreover, nsPEF exposure mitigates the cancerous potential of the cells in metabolomic and lipidomic studies and no pro-survival nor pro-metastatic potential of the cells is observed in functional studies. Similar effects were observed both in 3D cell culture models and studies of patients’ derived tumor ex vivo models. Statistical analysis of patients’ survival depending on the expression level of MAGE antigens showed increased survival when the expression of the antigen is enhanced. Our study suggests that nsPEF may positively affect the anti-melanoma response and make the cells more vulnerable to therapy, while not directly affecting their viability.

### Supplementary Information

Supplementary Information.

## Data Availability

The datasets generated during and/or analyzed during the current study are available from the corresponding author on reasonable request. Data is also provided within the manuscript or supplementary information files.
