# Supplementary material for: Pulsed electric field induces exocytosis and overexpression of MAGE antigens in melanoma
Source: Sci Rep. 2024 May 31;14:12546. doi: 10.1038/s41598-024-63181-x (PMC11143327; doi:10.1038/s41598-024-63181-x)
Supplement: Supplementary file 1 — Supplementary Information. [file 41598_2024_63181_MOESM1_ESM.pdf]

Supplementary materials

Western Blot:

Pages	Protein
1	Front page
2-5	PD-1
6-9	Beta-actin for PD-1 experiment
10-19	MAGE-A1
20-25	MAGE-C2
26-32	MAGE-3
33-35	Beta-actin for MAGE experiment

Fig. 3A was prepared using blot from pages: 14, 21 and 34.
Fig. 3F was prepared using blot from pages: 2, 5 and 6.

Pages 36 and 37 show the layouts of the cropped WBs

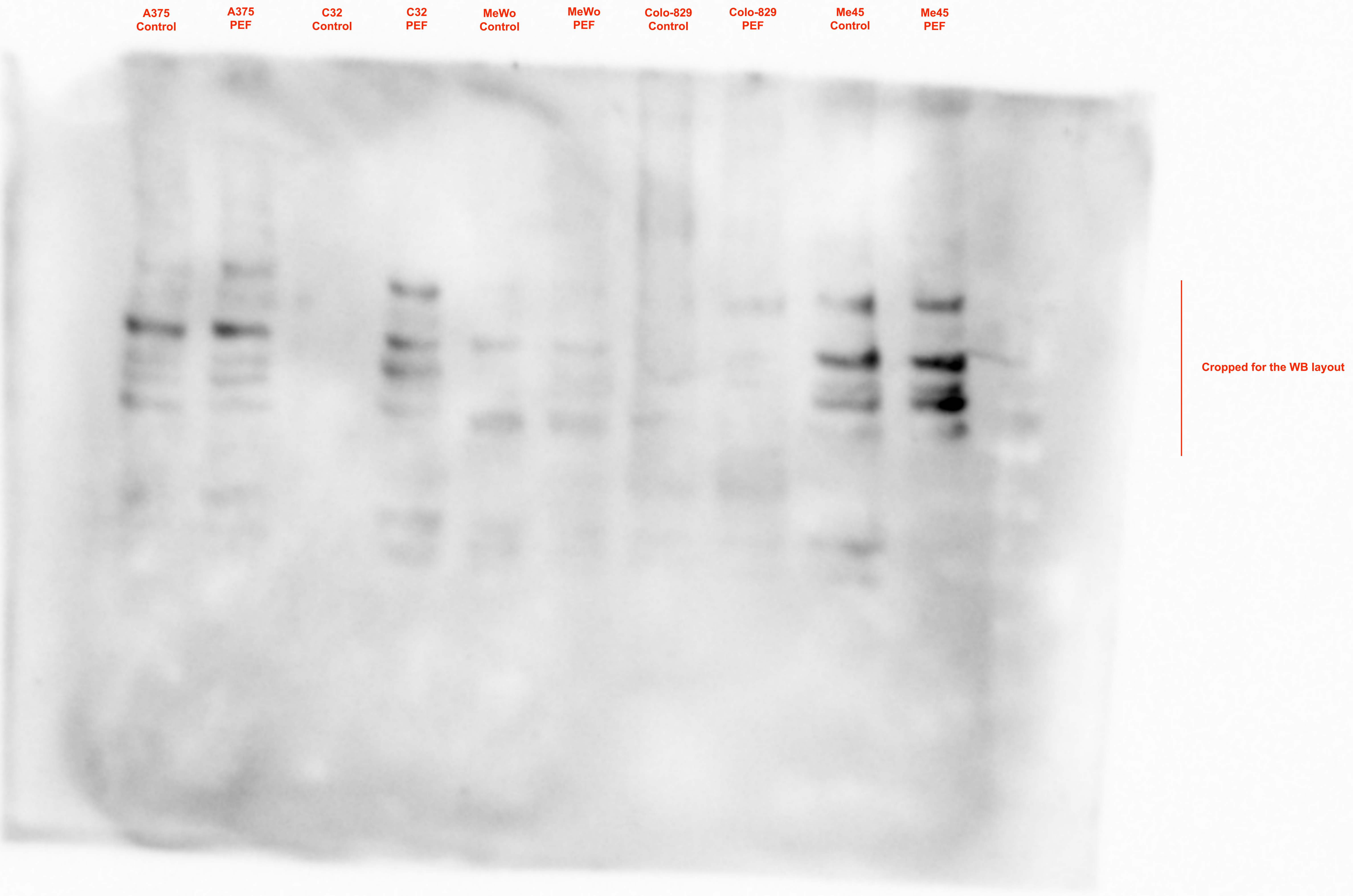
A375 Control
A375 PEF
C32 Control
C32 PEF
MeWo Control
MeWo PEF
Colo-829 Control
Colo-829 PEF
Me45 Control
Me45 PEF
Cropped for the WB layout

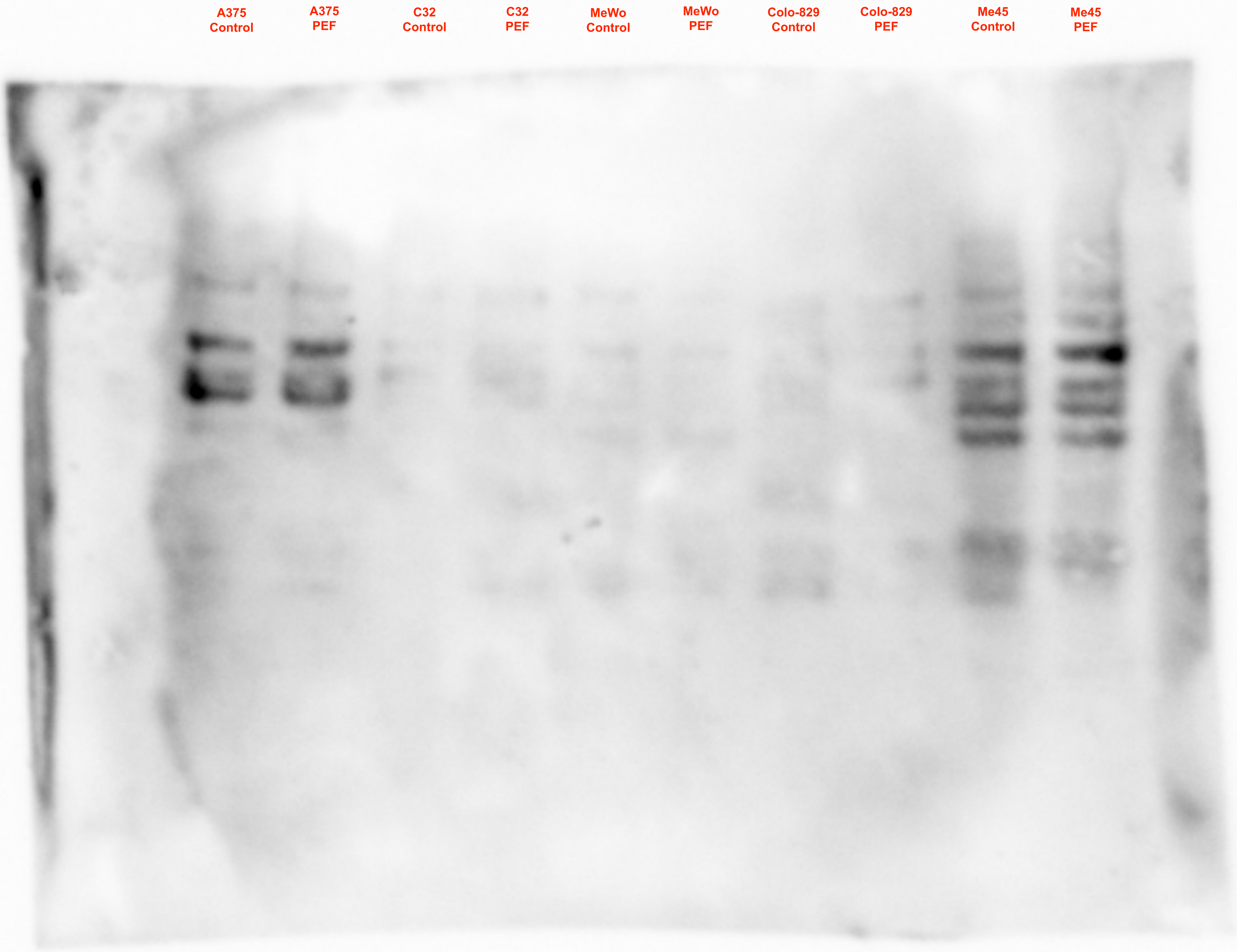
A375 Control
A375 PEF
C32 Control
C32 PEF
MeWo Control
MeWo PEF
Colo-829 Control
Colo-829 PEF
Me45 Control
Me45 PEF

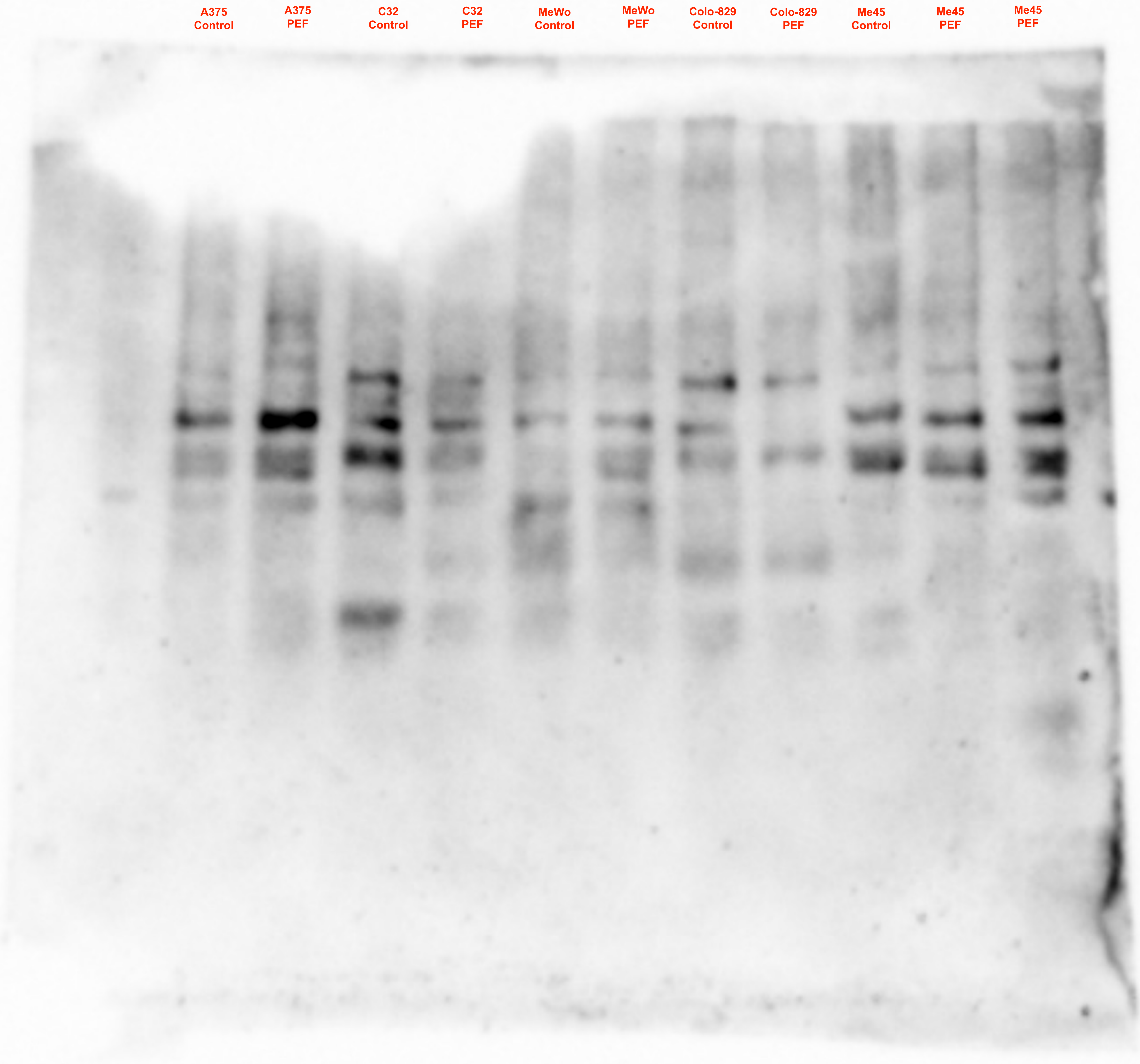

A375 Control
A375 PEF
C32 Control
C32 PEF
MeWo Control
MeWo PEF
Colo-829 Control
Colo-829 PEF
Me45 Control
Me45 PEF
Me45 PEF

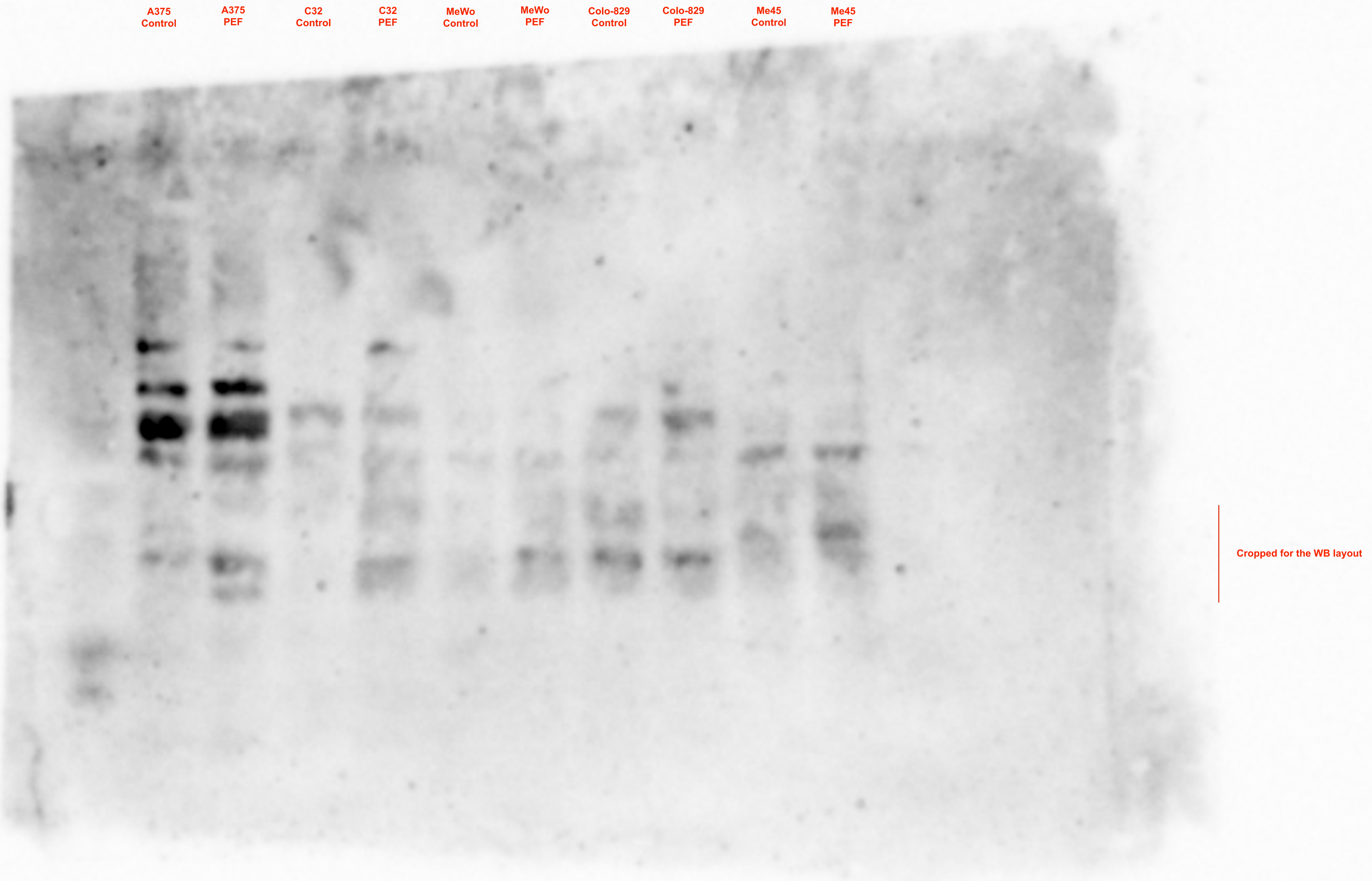
A375 Control
A375 PEF
C32 Control
C32 PEF
MeWo Control
MeWo PEF
Colo-829 Control
Colo-829 PEF
Me45 Control
Me45 PEF
Cropped for the WB layout

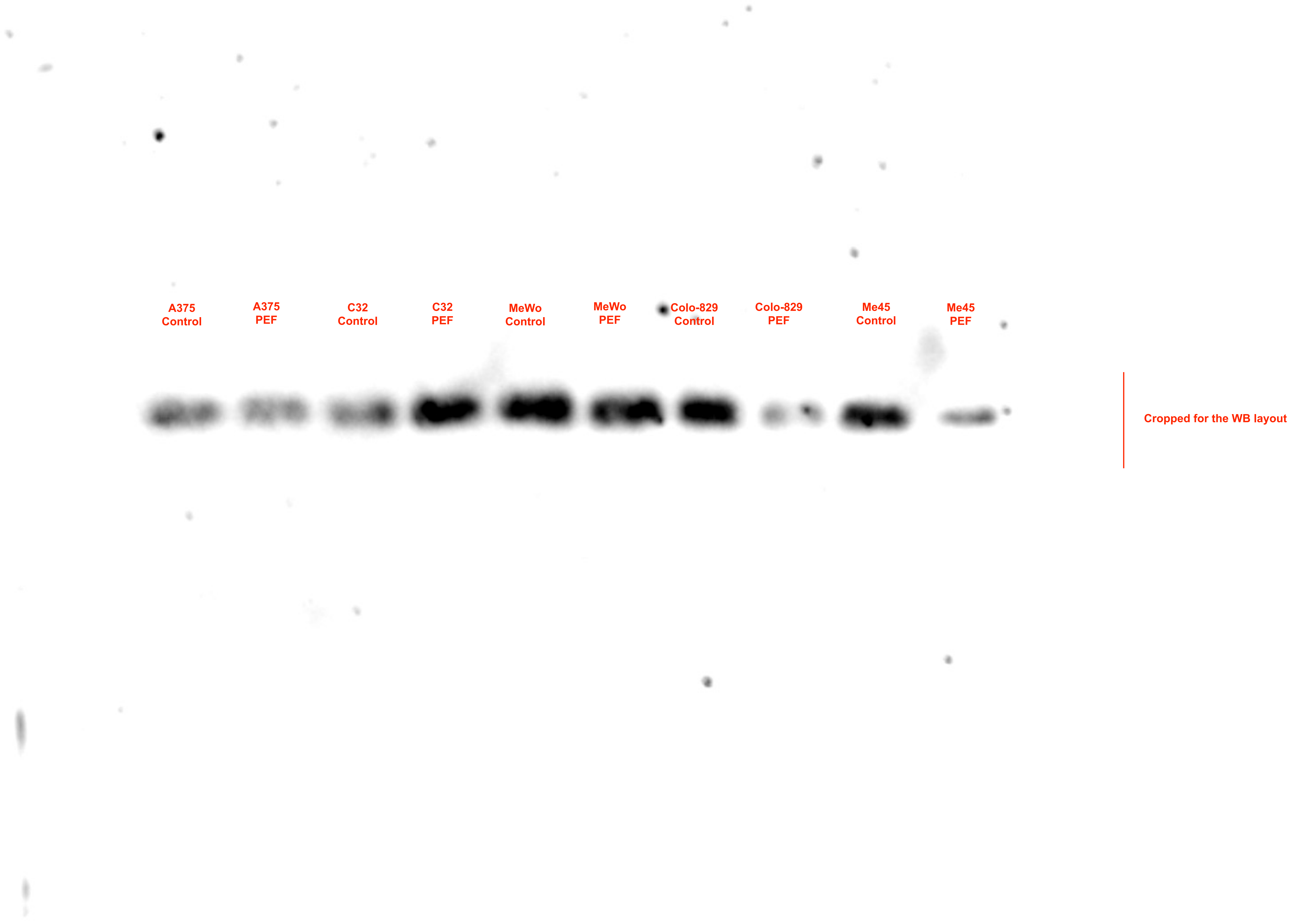
A375 Control
A375 PEF
C32 Control
C32 PEF
MeWo Control
MeWo PEF
Colo-829 Control
Colo-829 PEF
Me45 Control
Me45 PEF
Cropped for the WB layout

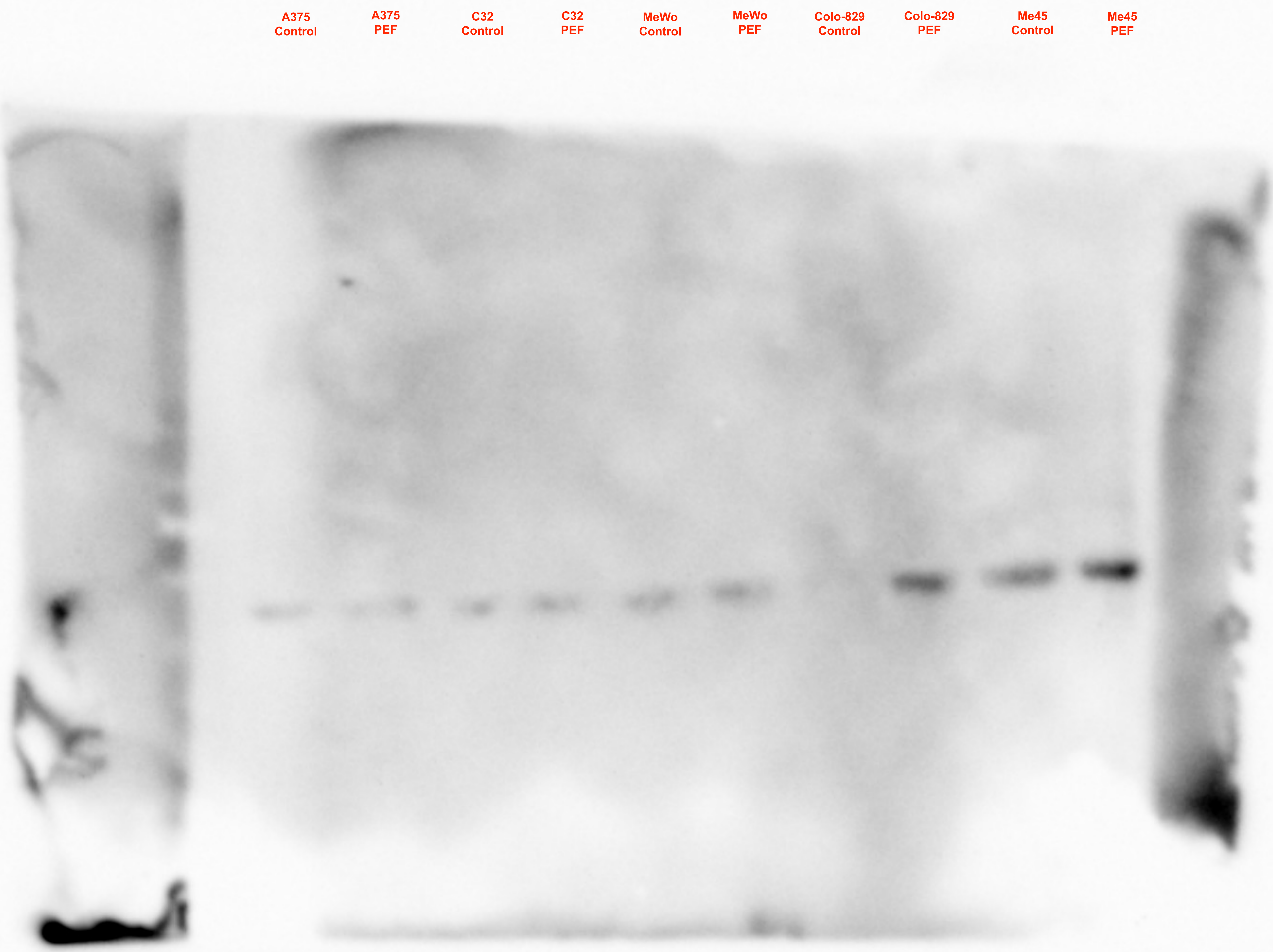
A375 Control
A375 PEF
C32 Control
C32 PEF
MeWo Control
MeWo PEF
Colo-829 Control
Colo-829 PEF
Me45 Control
Me45 PEF

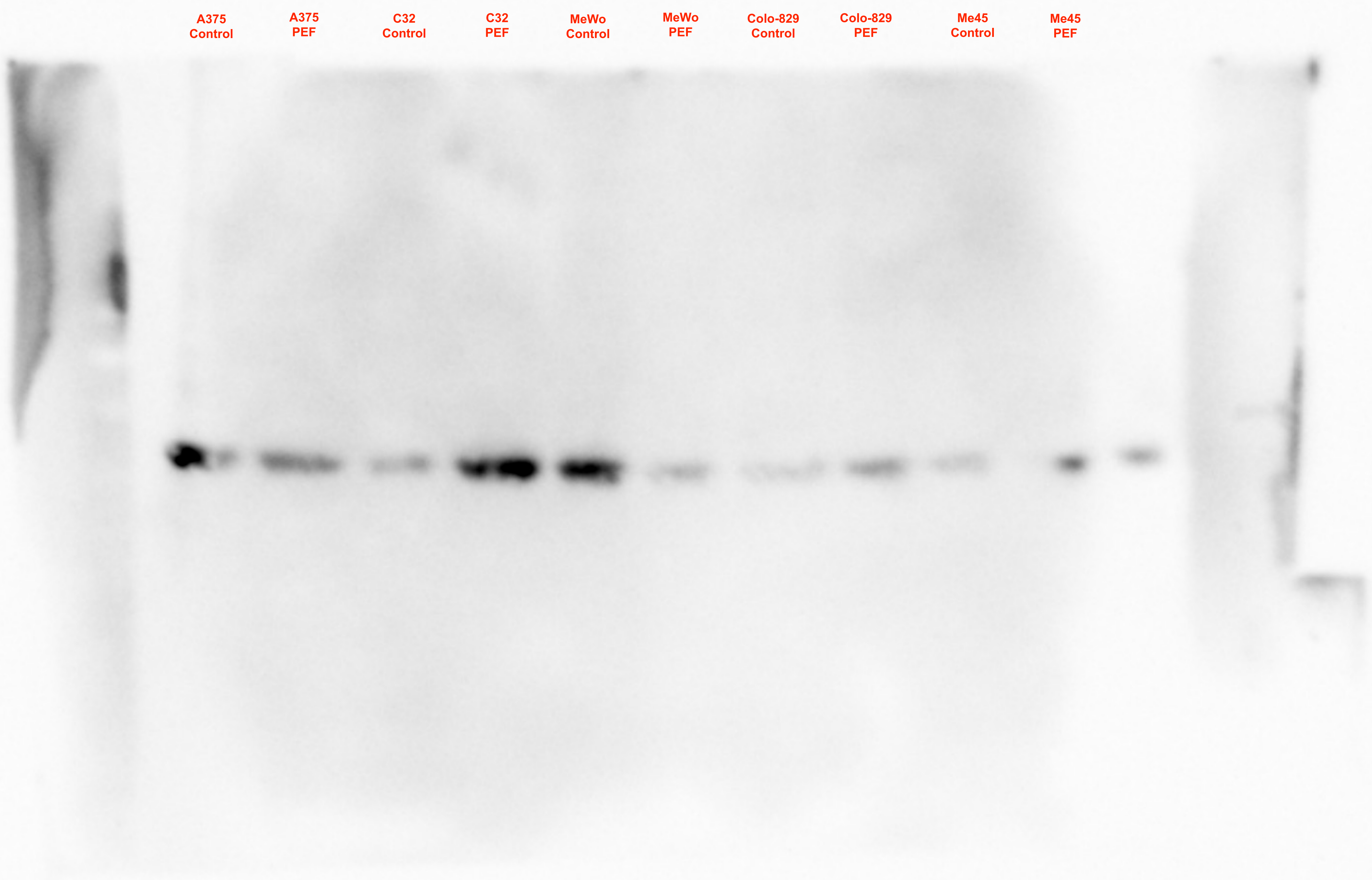
A375 Control
A375 PEF
C32 Control
C32 PEF
MeWo Control
MeWo PEF
Colo-829 Control
Colo-829 PEF
Me45 Control
Me45 PEF

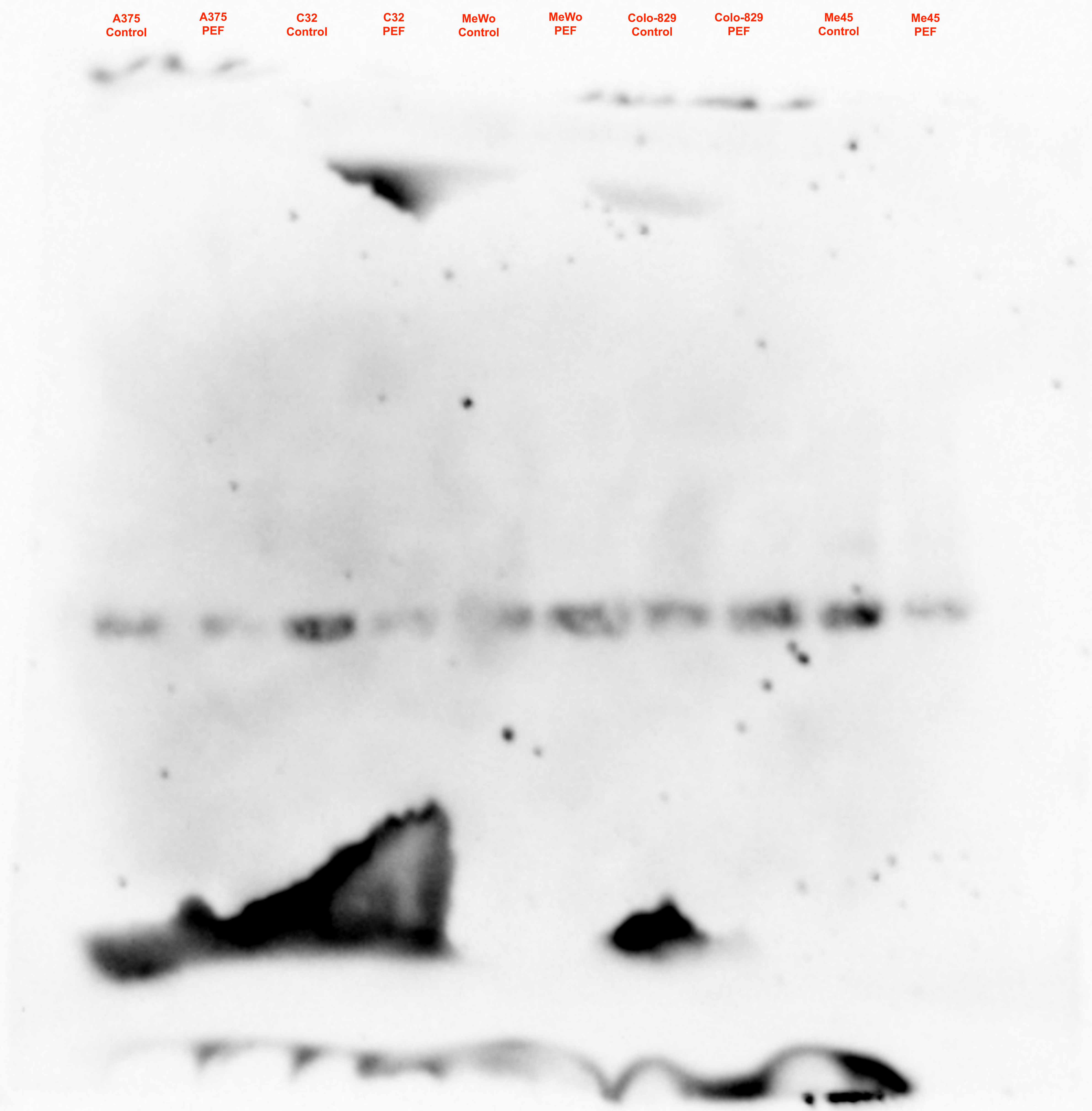
A375 Control
A375 PEF
C32 Control
C32 PEF
MeWo Control
MeWo PEF
Colo-829 Control
Colo-829 PEF
Me45 Control
Me45 PEF

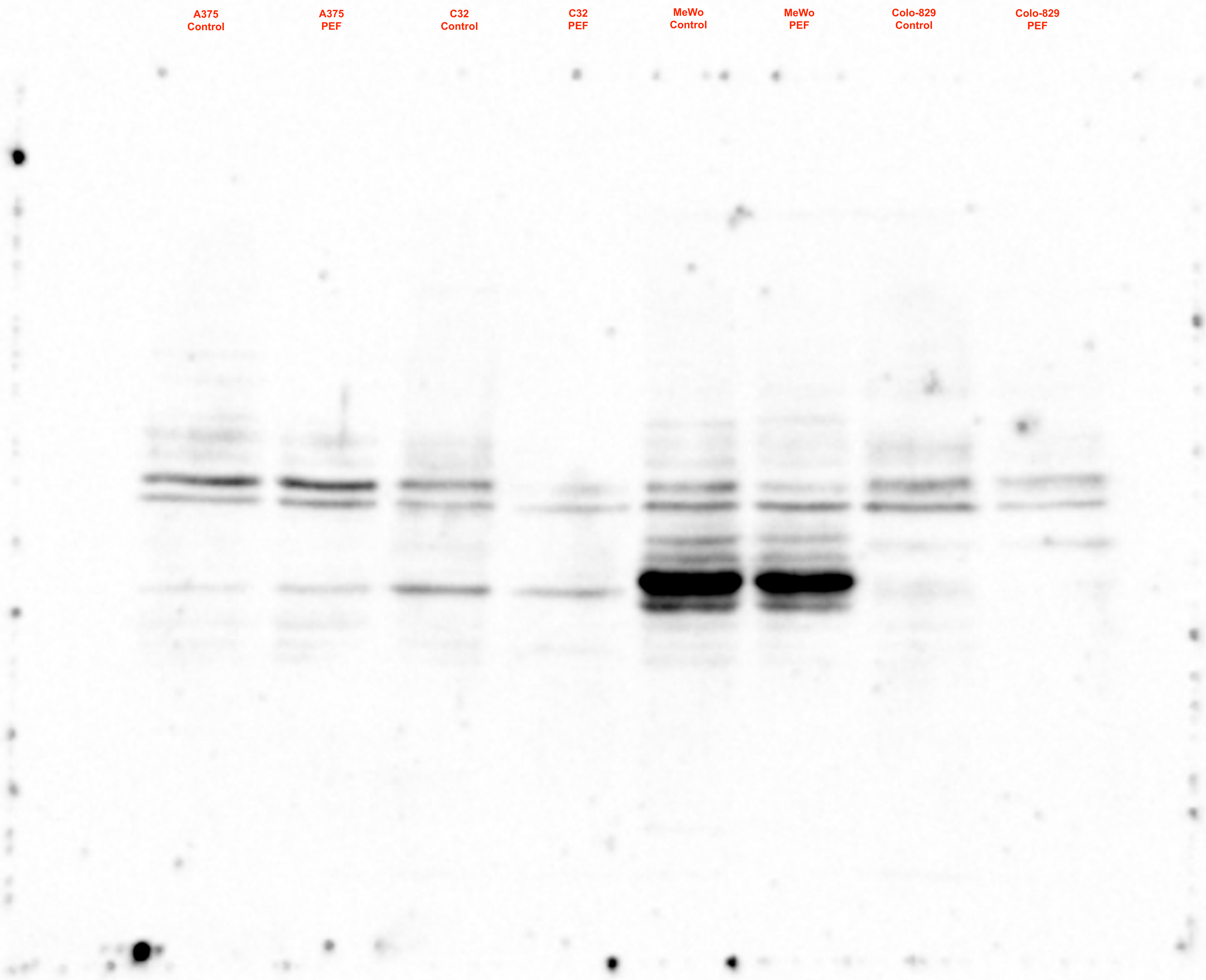
A375 Control
A375 PEF
C32 Control
C32 PEF
MeWo Control
MeWo PEF
Colo-829 Control
Colo-829 PEF

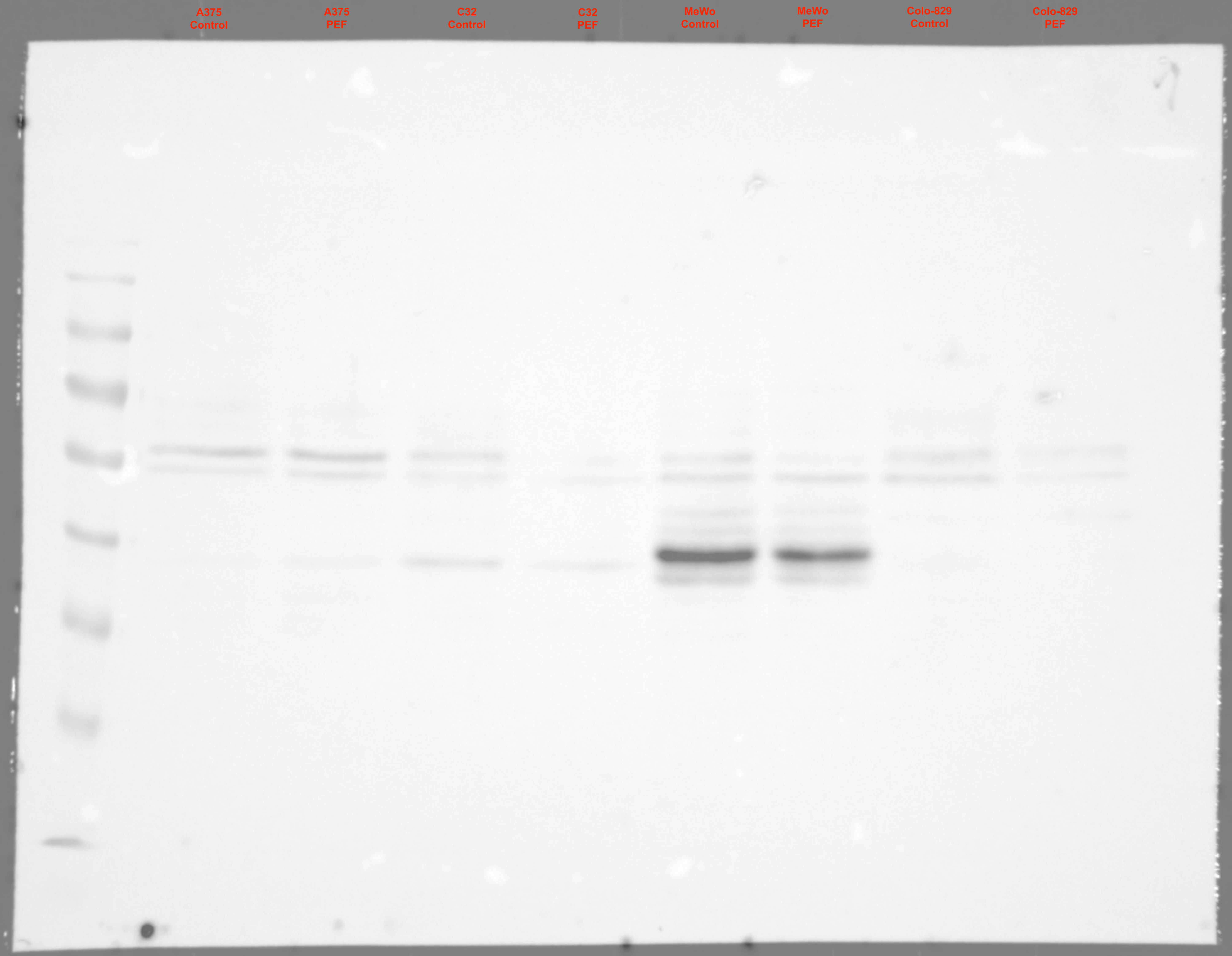
A375 Control
A375 PEF
C32 Control
C32 PEF
MeWo Control
MeWo PEF
Colo-829 Control
Colo-829 PEF

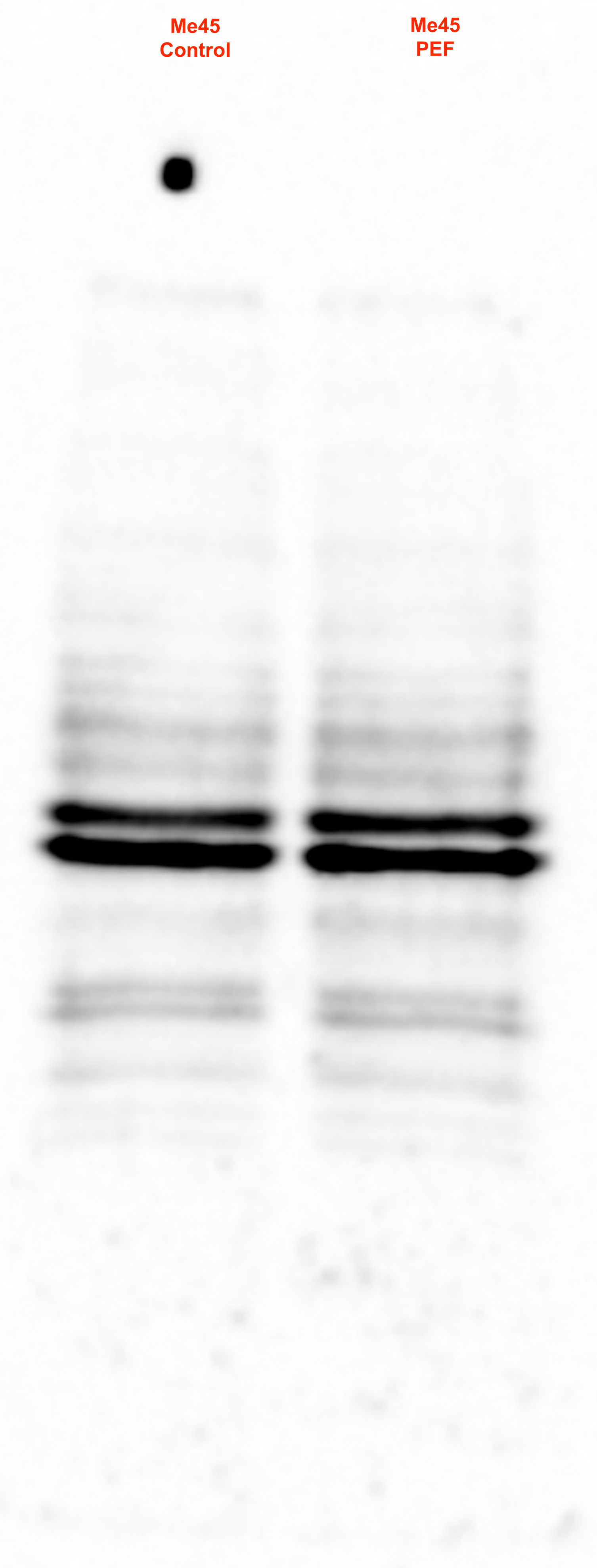
Me45
Control
Me45
PEF

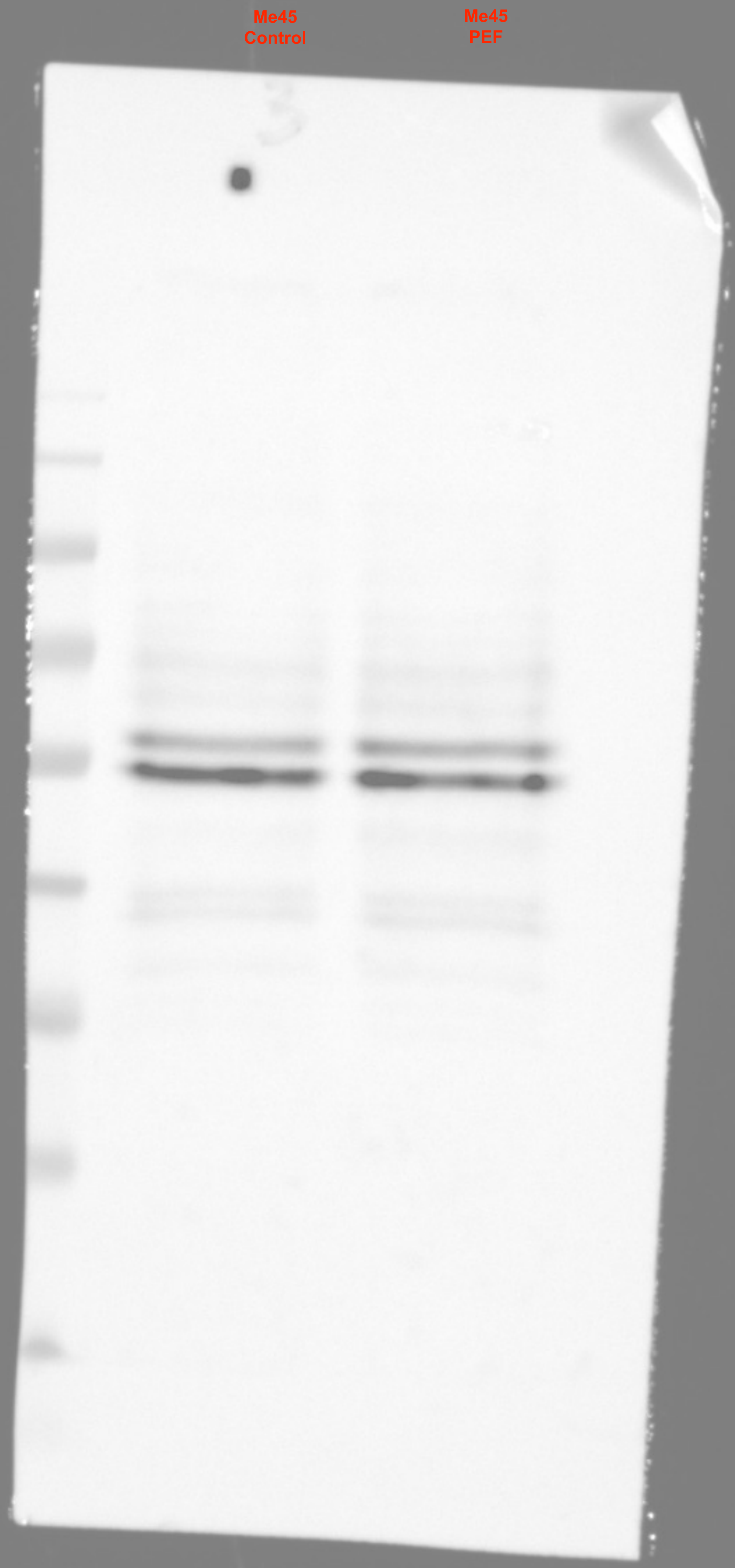

Me45
Control
Me45
PEF

A375 Control | A375 PEF | C32 Control | C32 PEF | MeWo Control | MeWo PEF | Colo-829 Control | Colo-829 PEF | Me45 Control | Me45 PEF

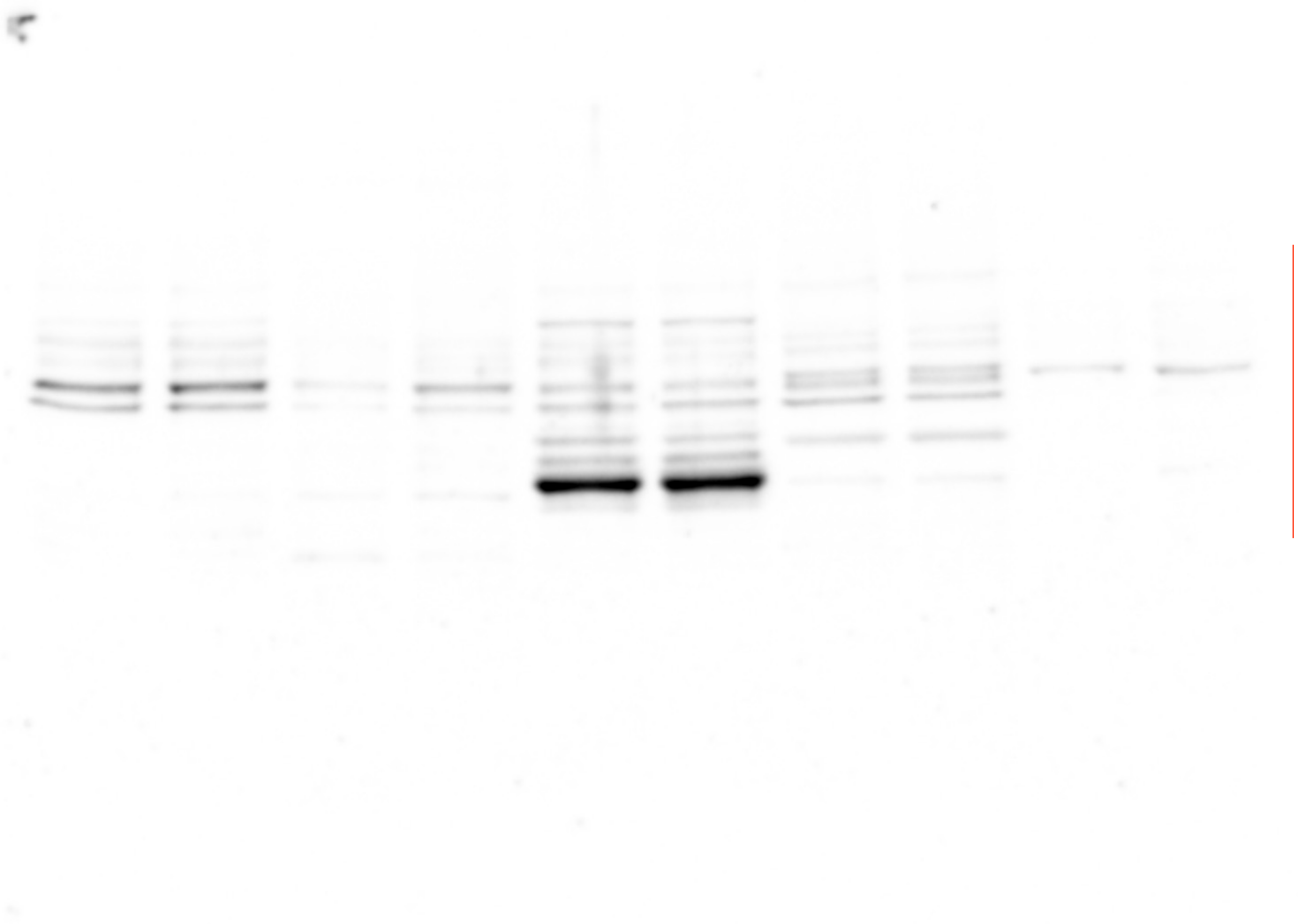

Cropped for the WB layout

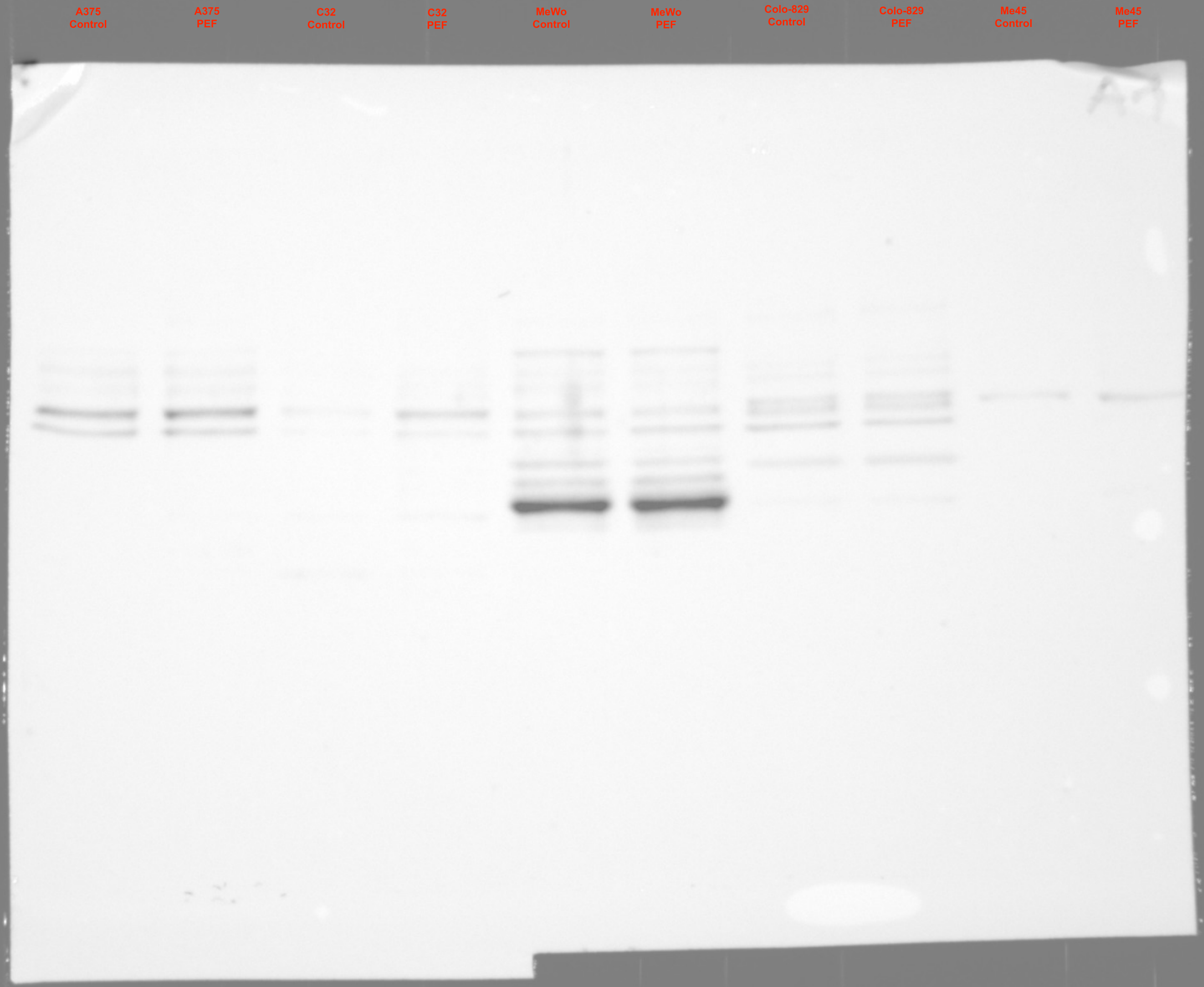

A375 Control
A375 PEF
C32 Control
C32 PEF
MeWo Control
MeWo PEF
Colo-829 Control
Colo-829 PEF
Me45 Control
Me45 PEF

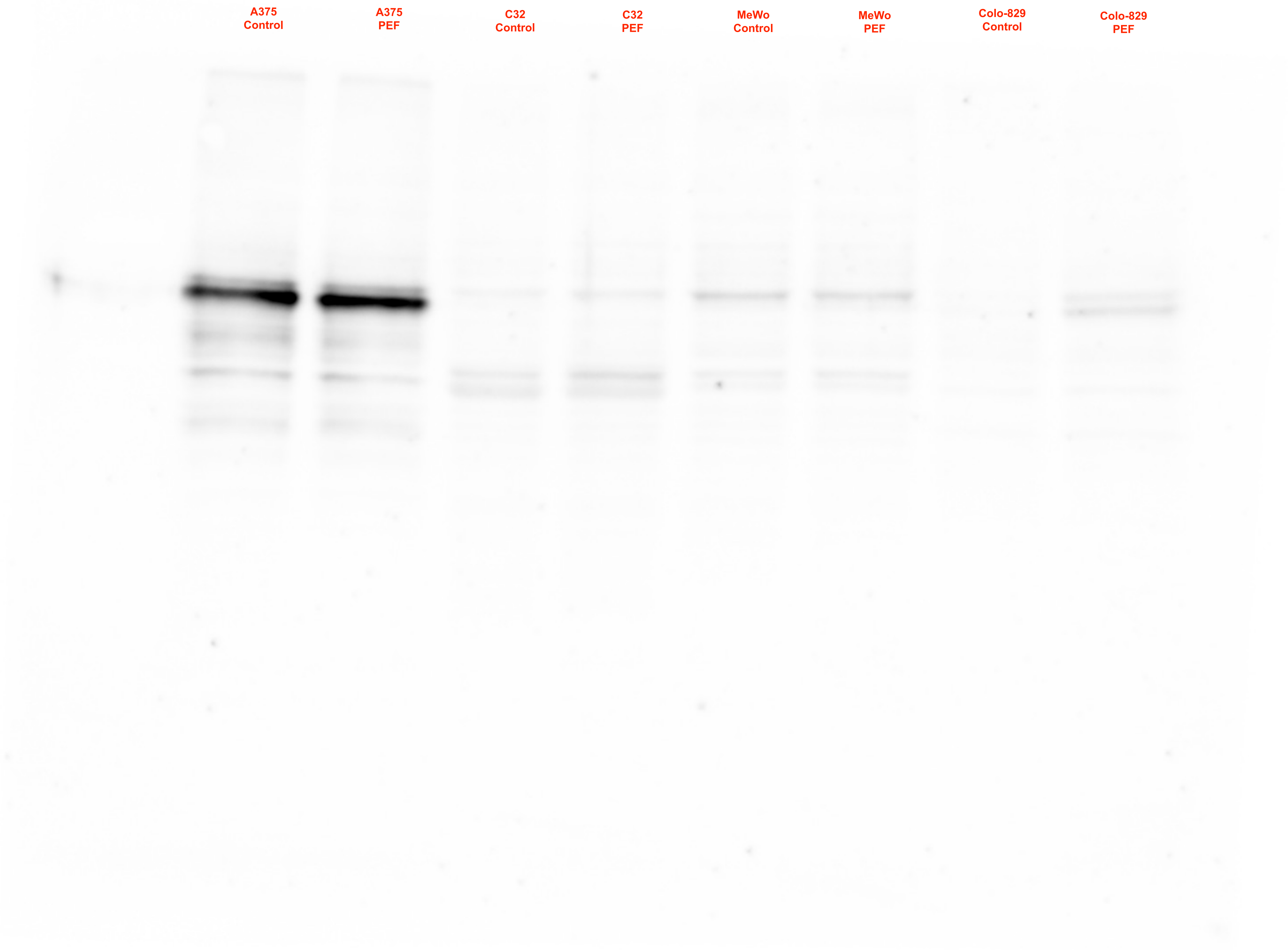
A375 Control
A375 PEF
C32 Control
C32 PEF
MeWo Control
MeWo PEF
Colo-829 Control
Colo-829 PEF

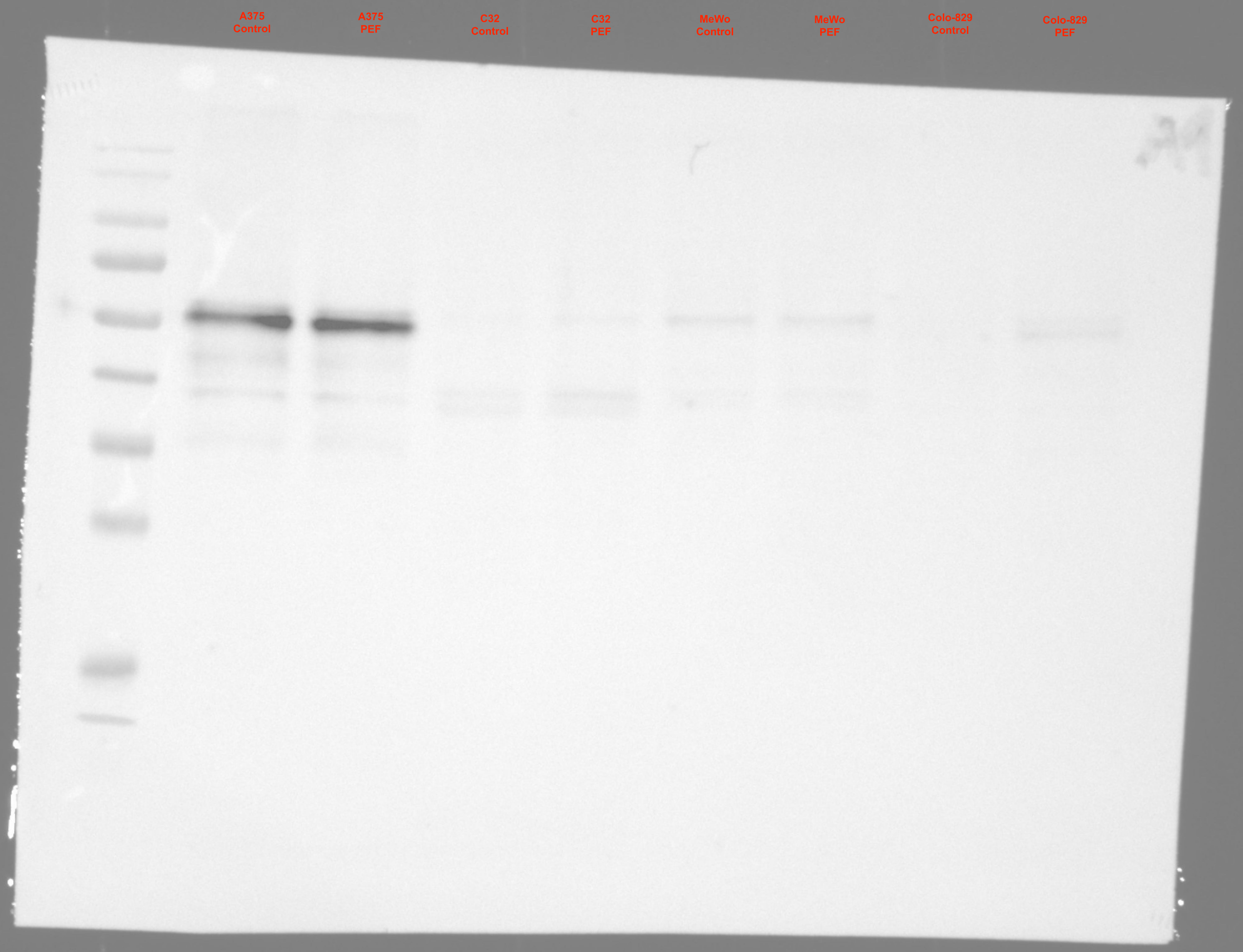

A375
Control
A375
PEF
C32
Control
C32
PEF
MeWo
Control
MeWo
PEF
Colo-829
Control
Colo-829
PEF

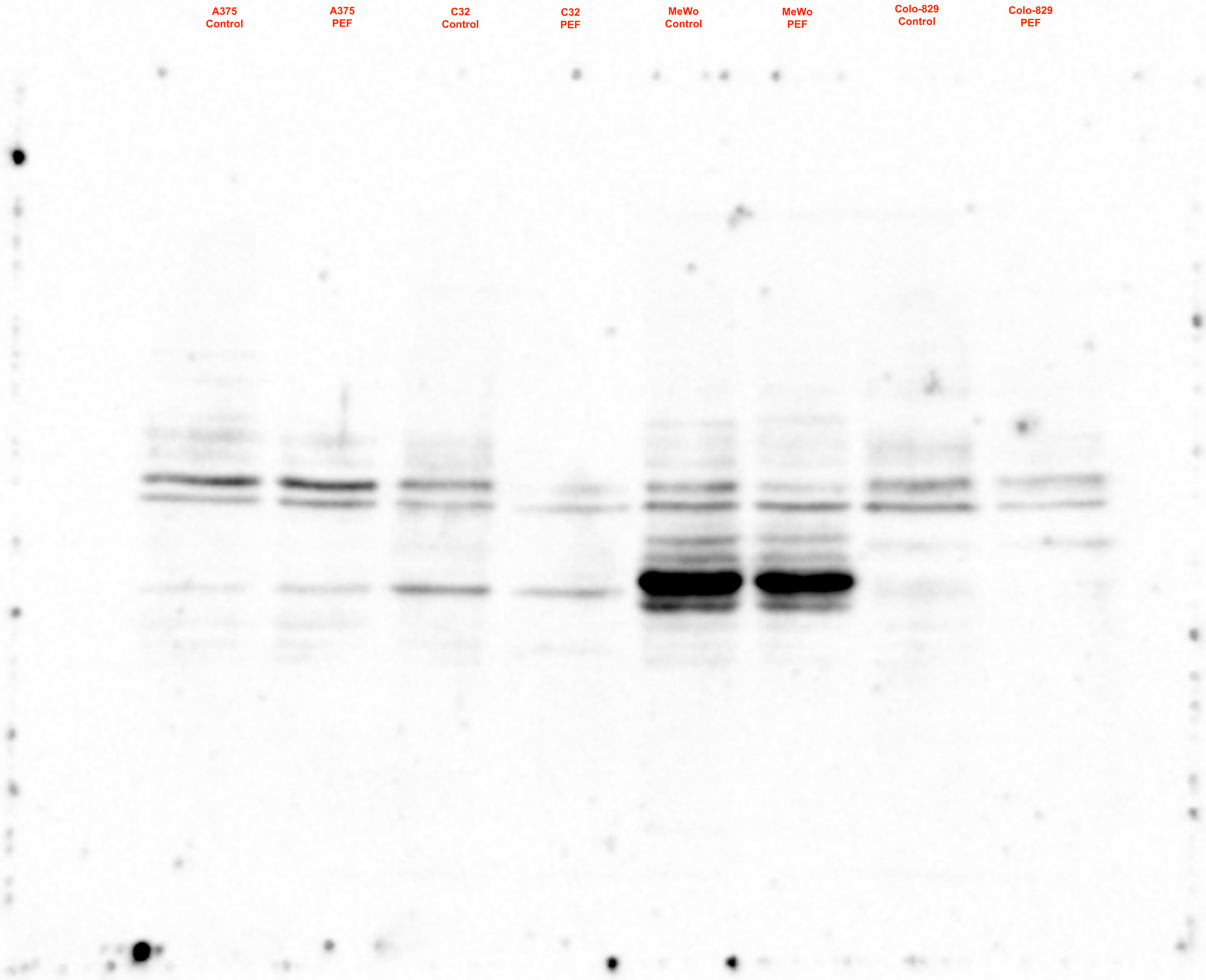
A375 Control
A375 PEF
C32 Control
C32 PEF
MeWo Control
MeWo PEF
Colo-829 Control
Colo-829 PEF

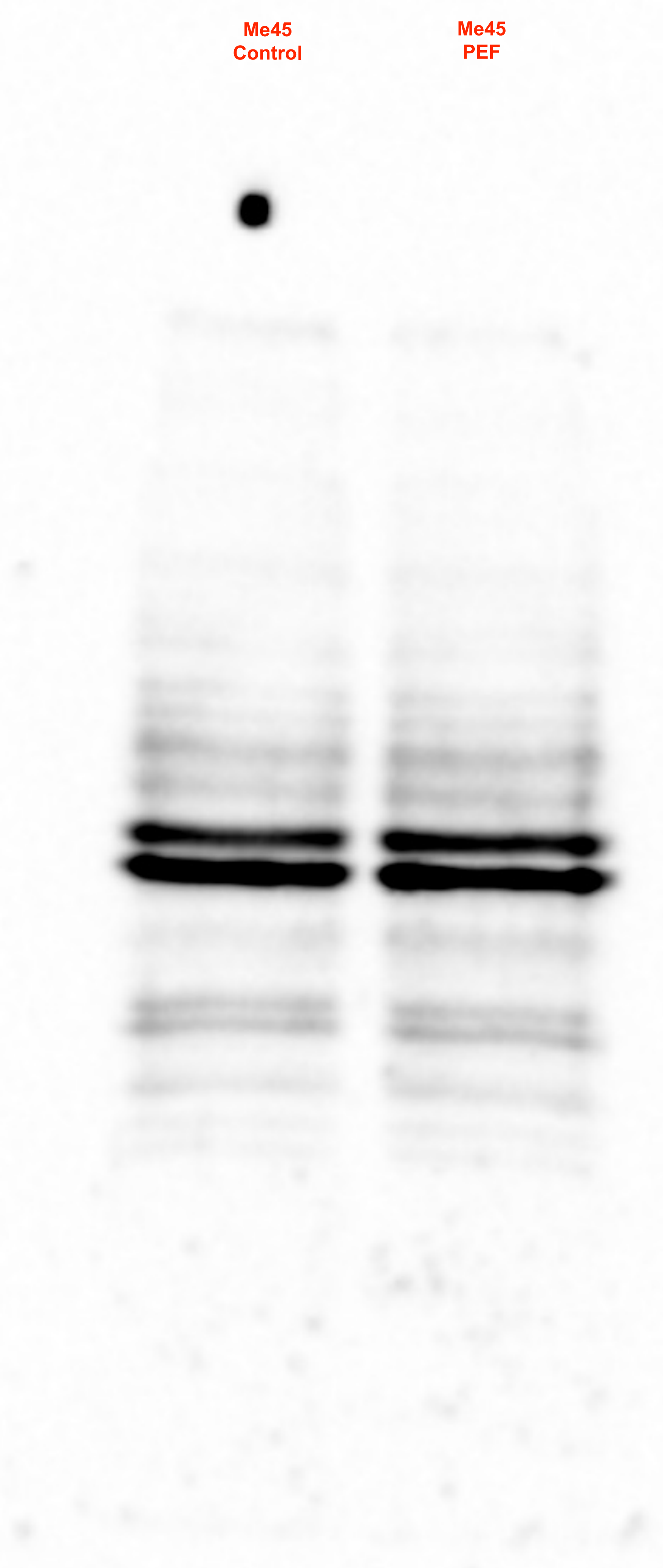
Me45
Control
Me45
PEF

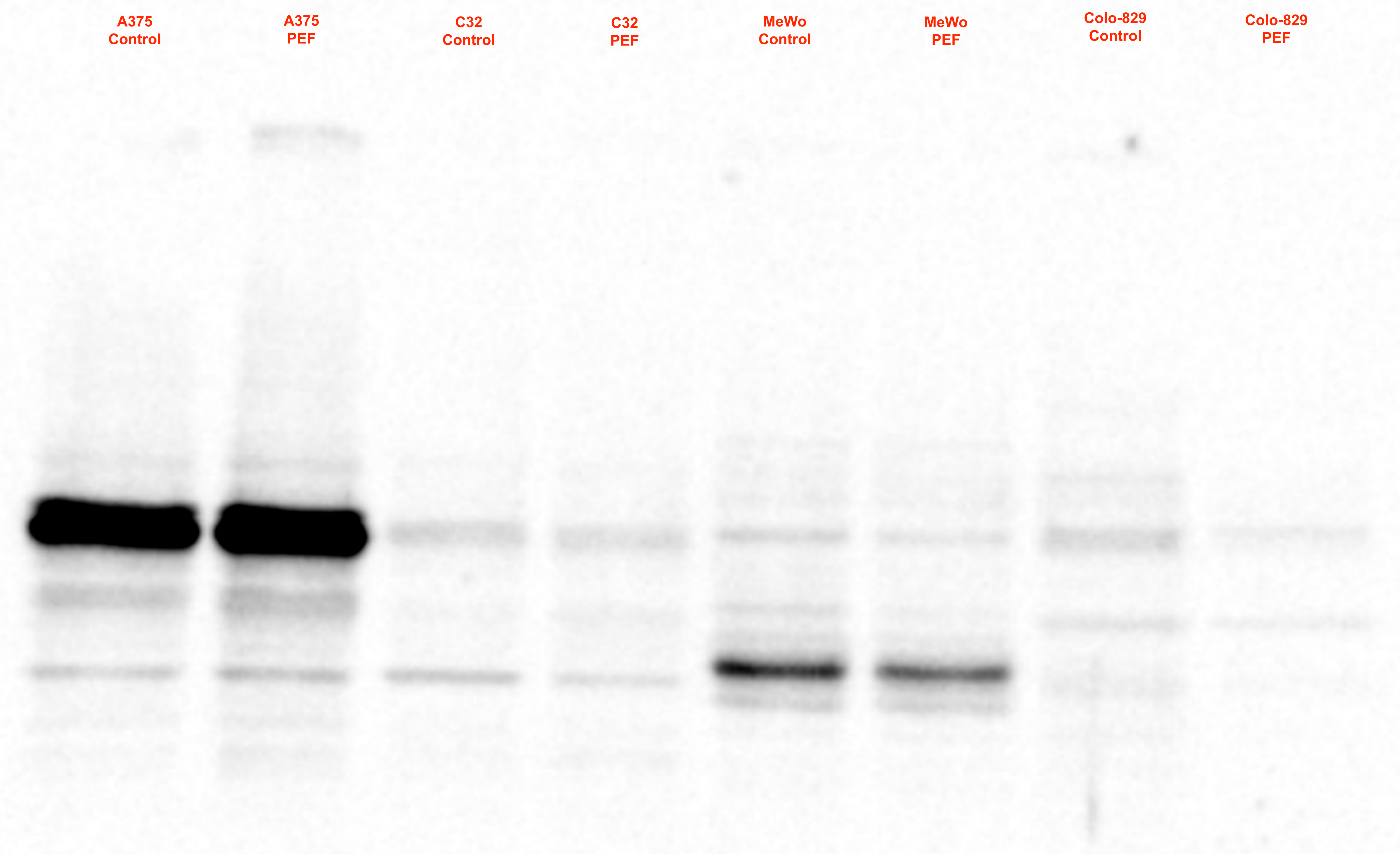
A375 Control
A375 PEF
C32 Control
C32 PEF
MeWo Control
MeWo PEF
Colo-829 Control
Colo-829 PEF

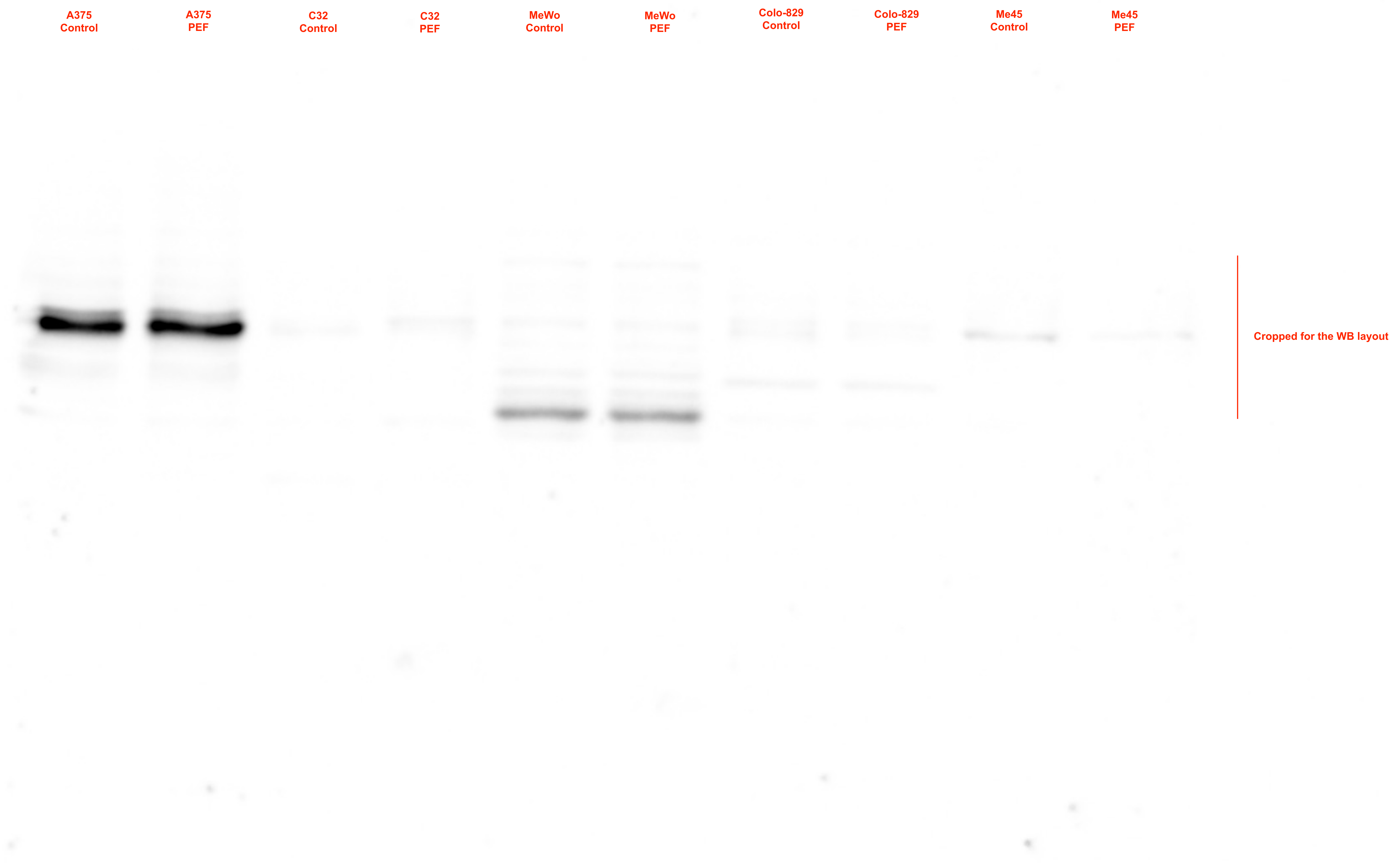
A375 Control
A375 PEF
C32 Control
C32 PEF
MeWo Control
MeWo PEF
Colo-829 Control
Colo-829 PEF
Me45 Control
Me45 PEF
Cropped for the WB layout

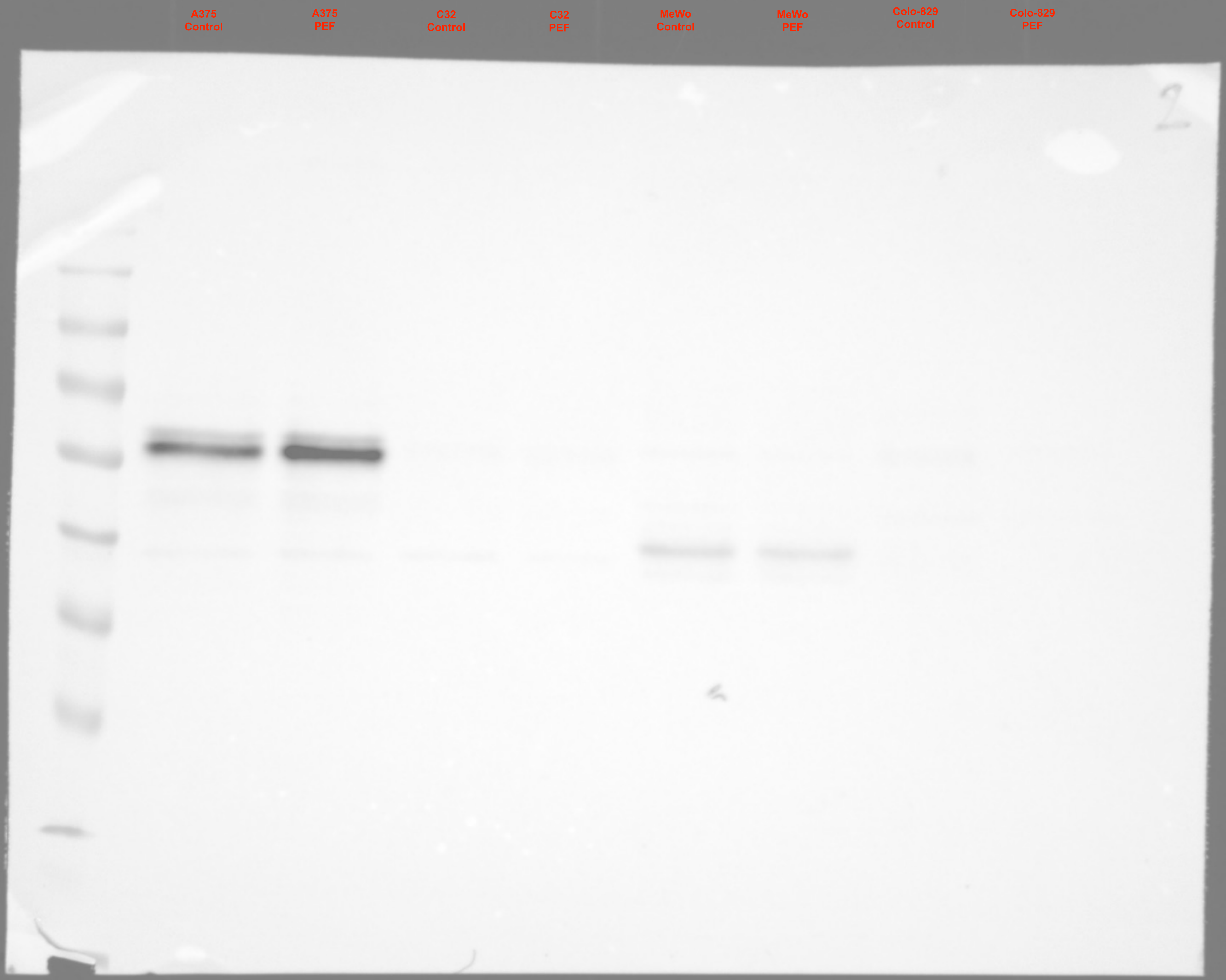
A375 Control
A375 PEF
C32 Control
C32 PEF
MeWo Control
MeWo PEF
Colo-829 Control
Colo-829 PEF
2

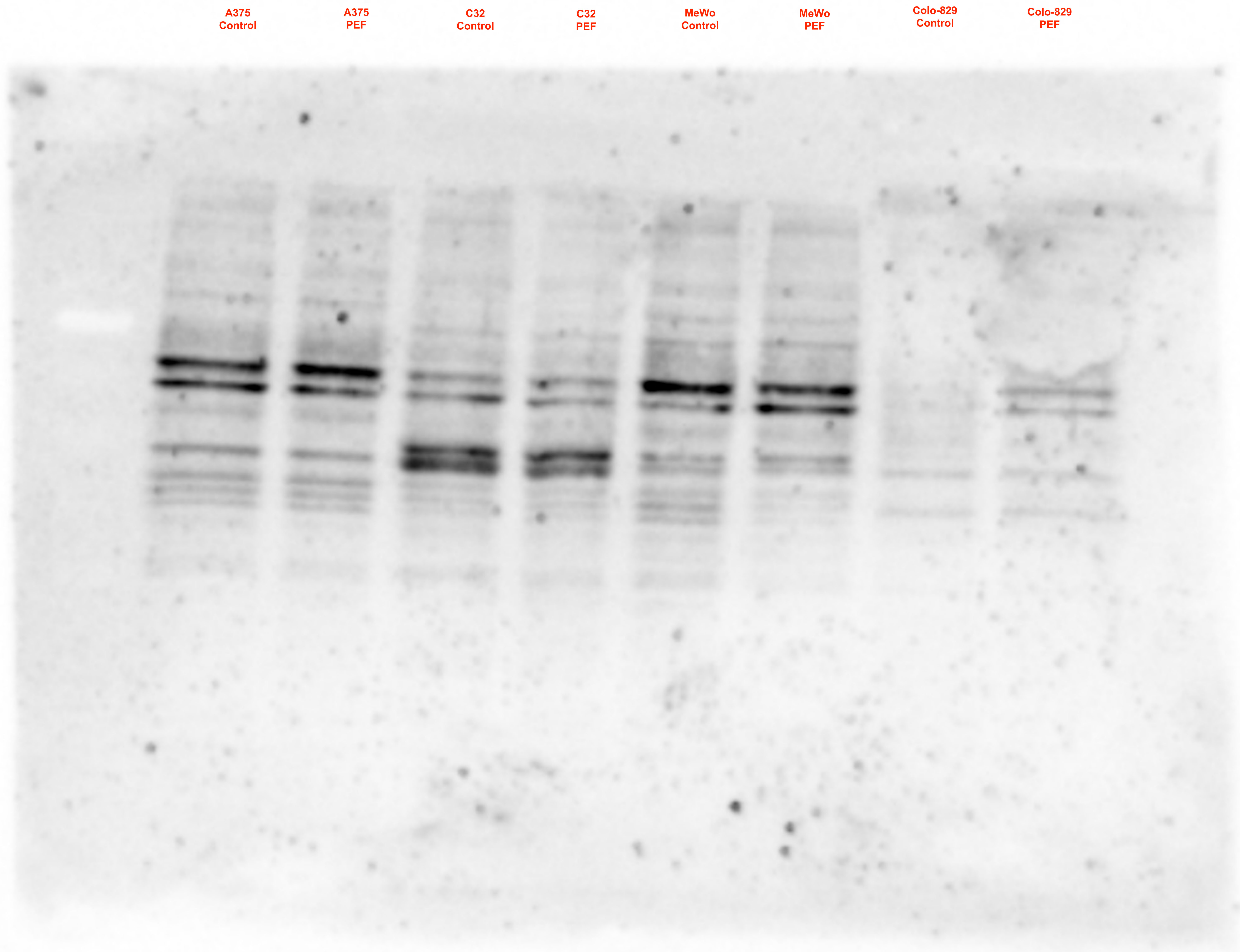
A375 Control
A375 PEF
C32 Control
C32 PEF
MeWo Control
MeWo PEF
Colo-829 Control
Colo-829 PEF

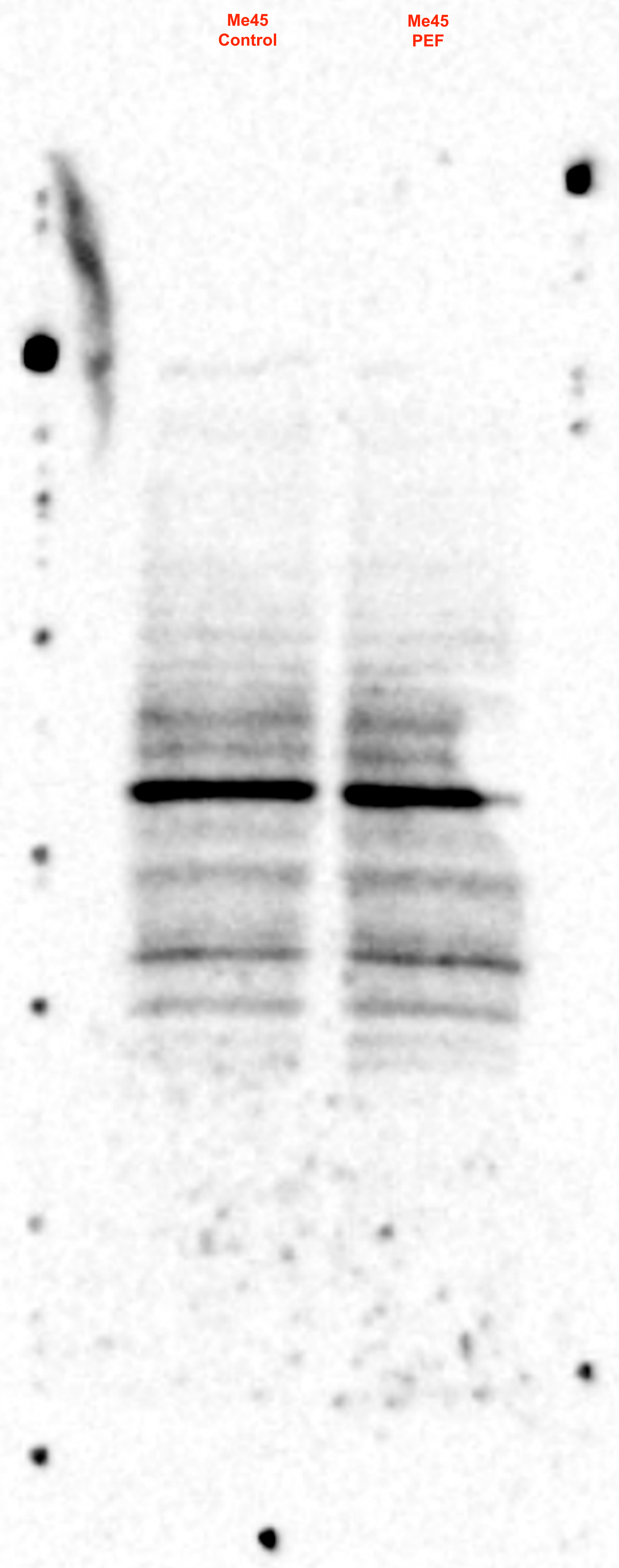

Me45
Control
Me45
PEF

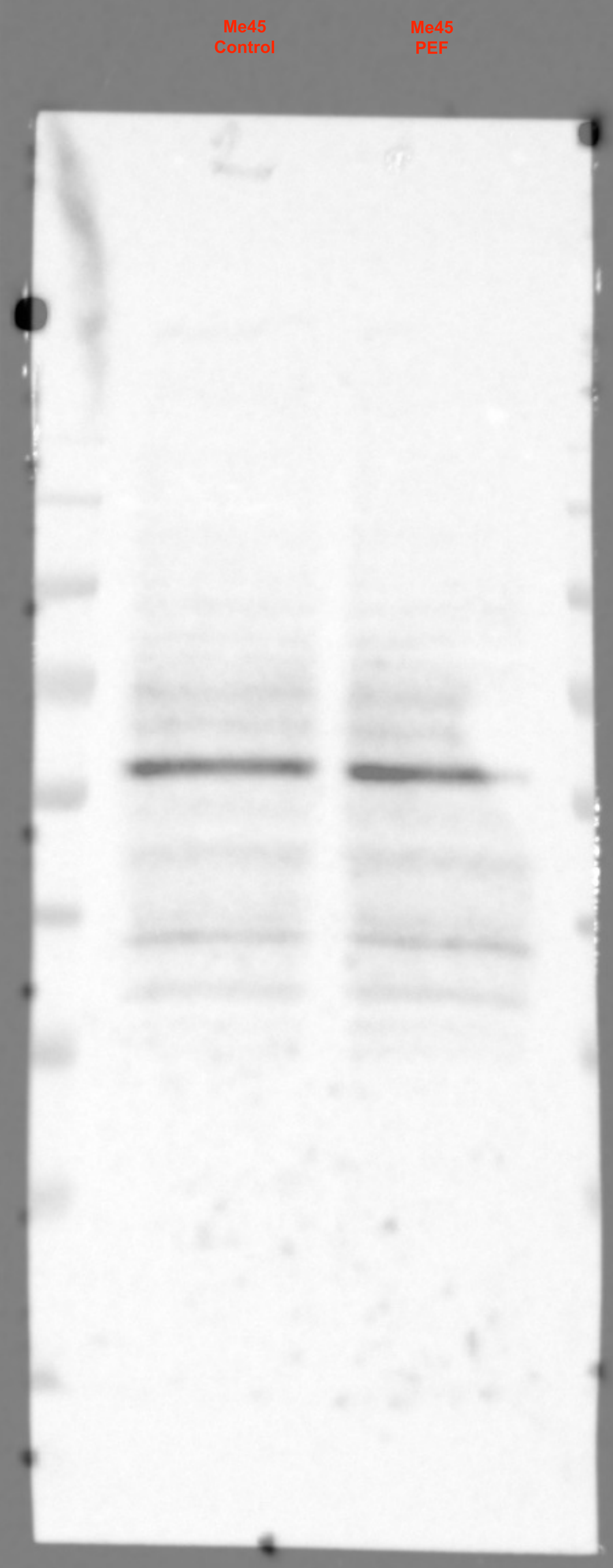
Me45
Control
Me45
PEF

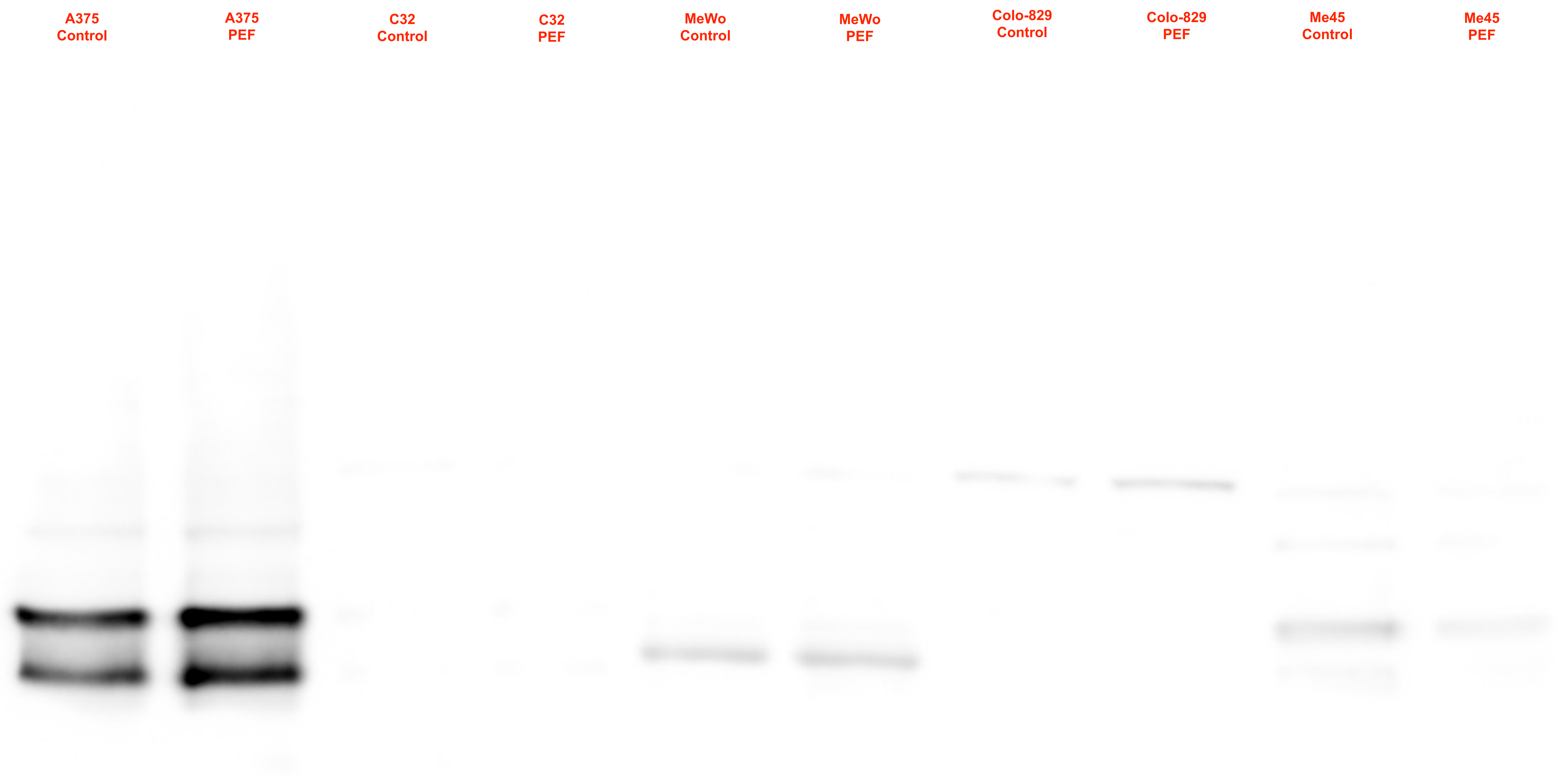
A375 Control
A375 PEF
C32 Control
C32 PEF
MeWo Control
MeWo PEF
Colo-829 Control
Colo-829 PEF
Me45 Control
Me45 PEF

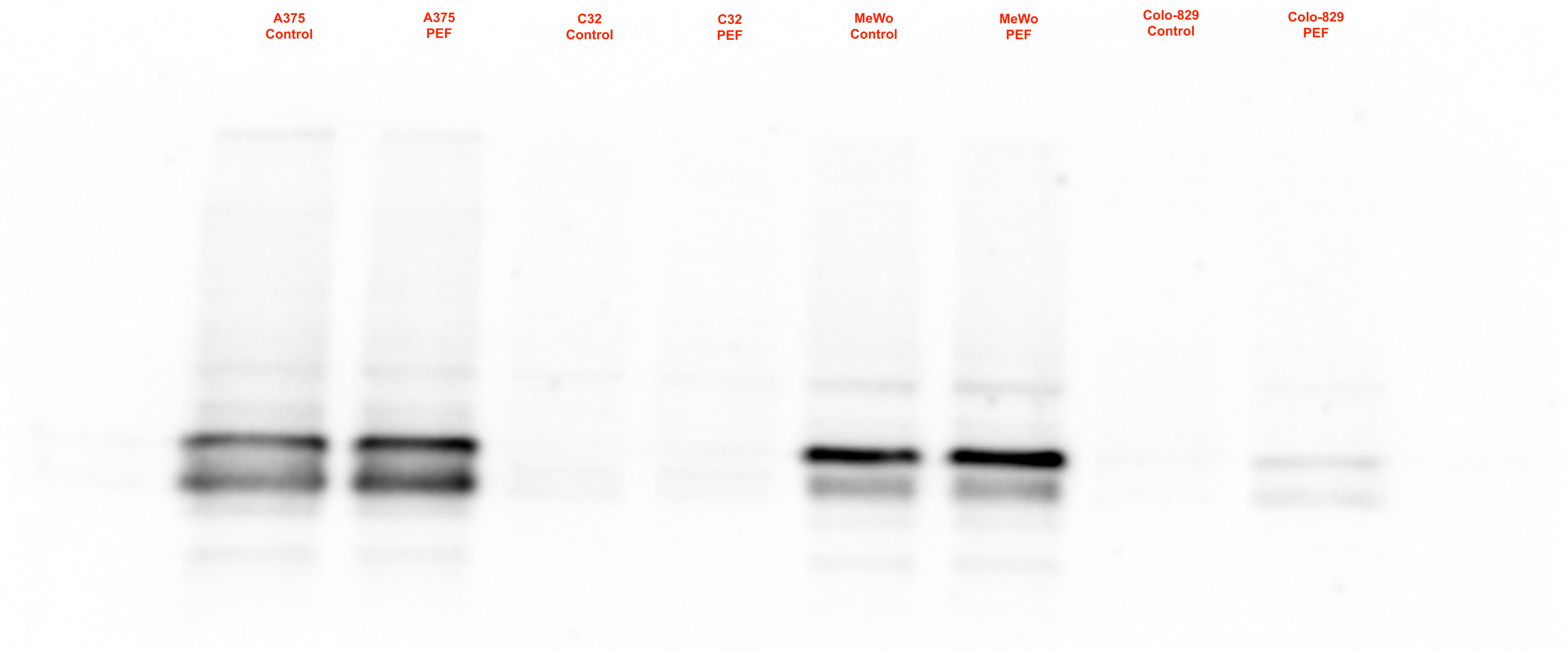
A375 Control
A375 PEF
C32 Control
C32 PEF
MeWo Control
MeWo PEF
Colo-829 Control
Colo-829 PEF

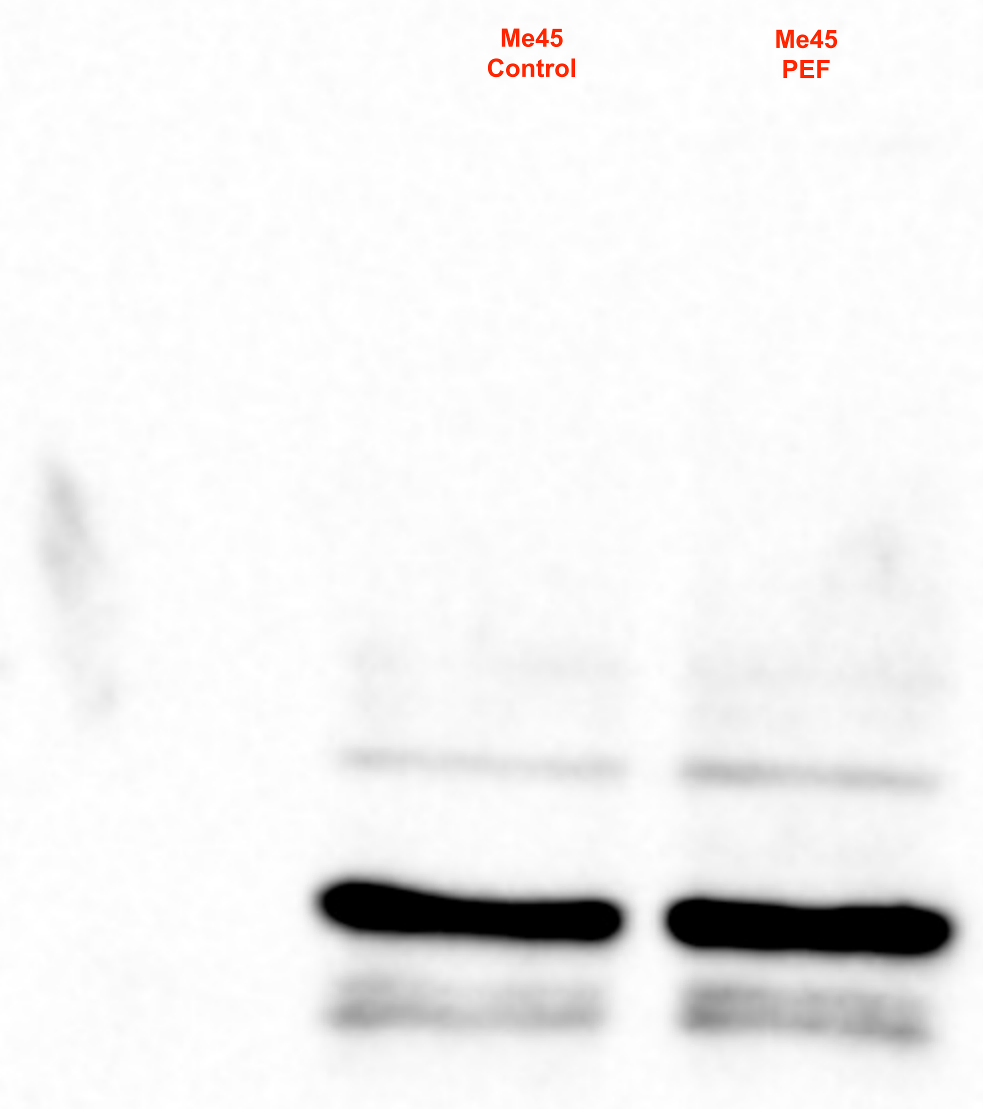
Me45
Control
Me45
PEF

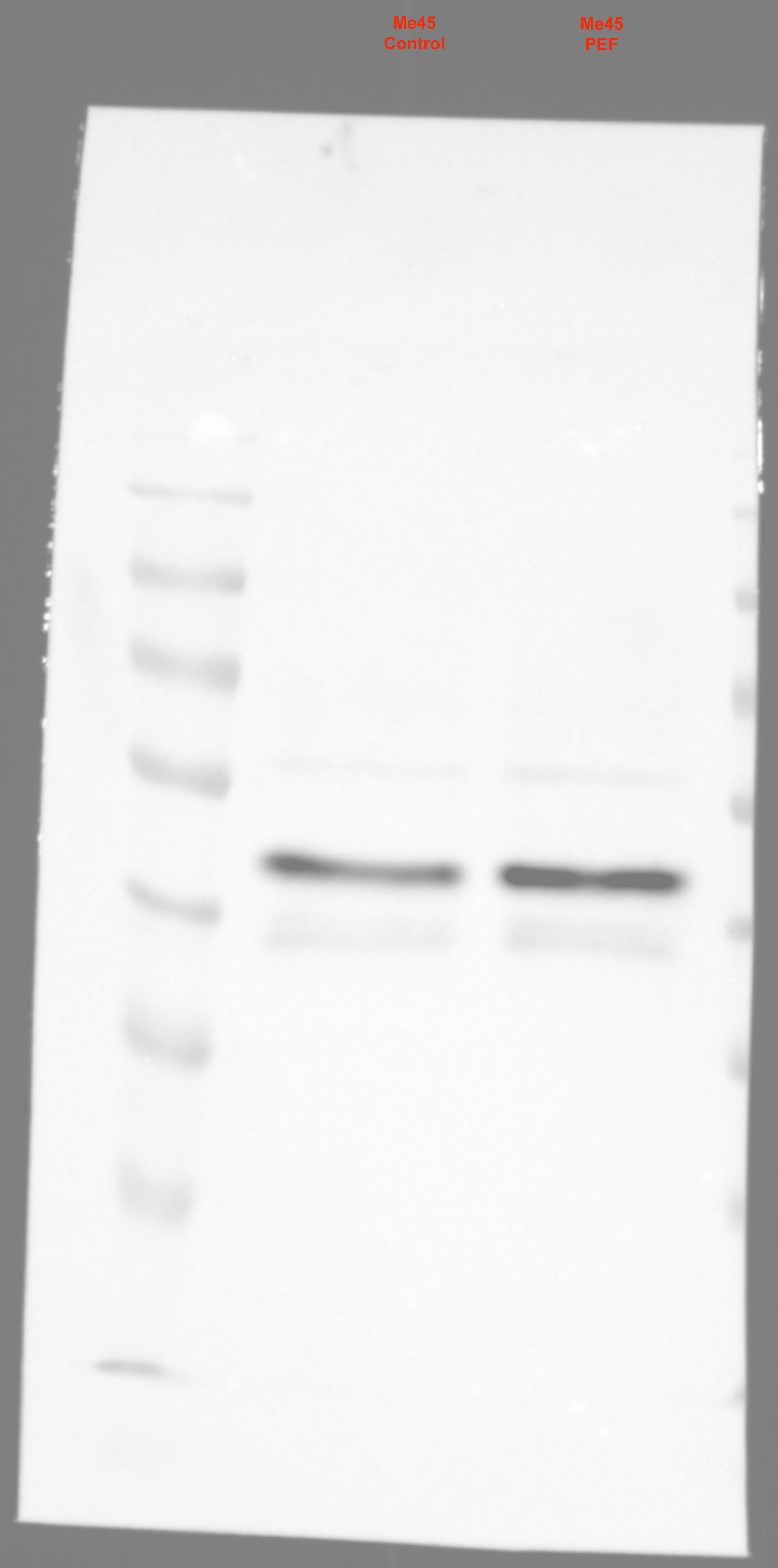
Me45
Control
Me45
PEF

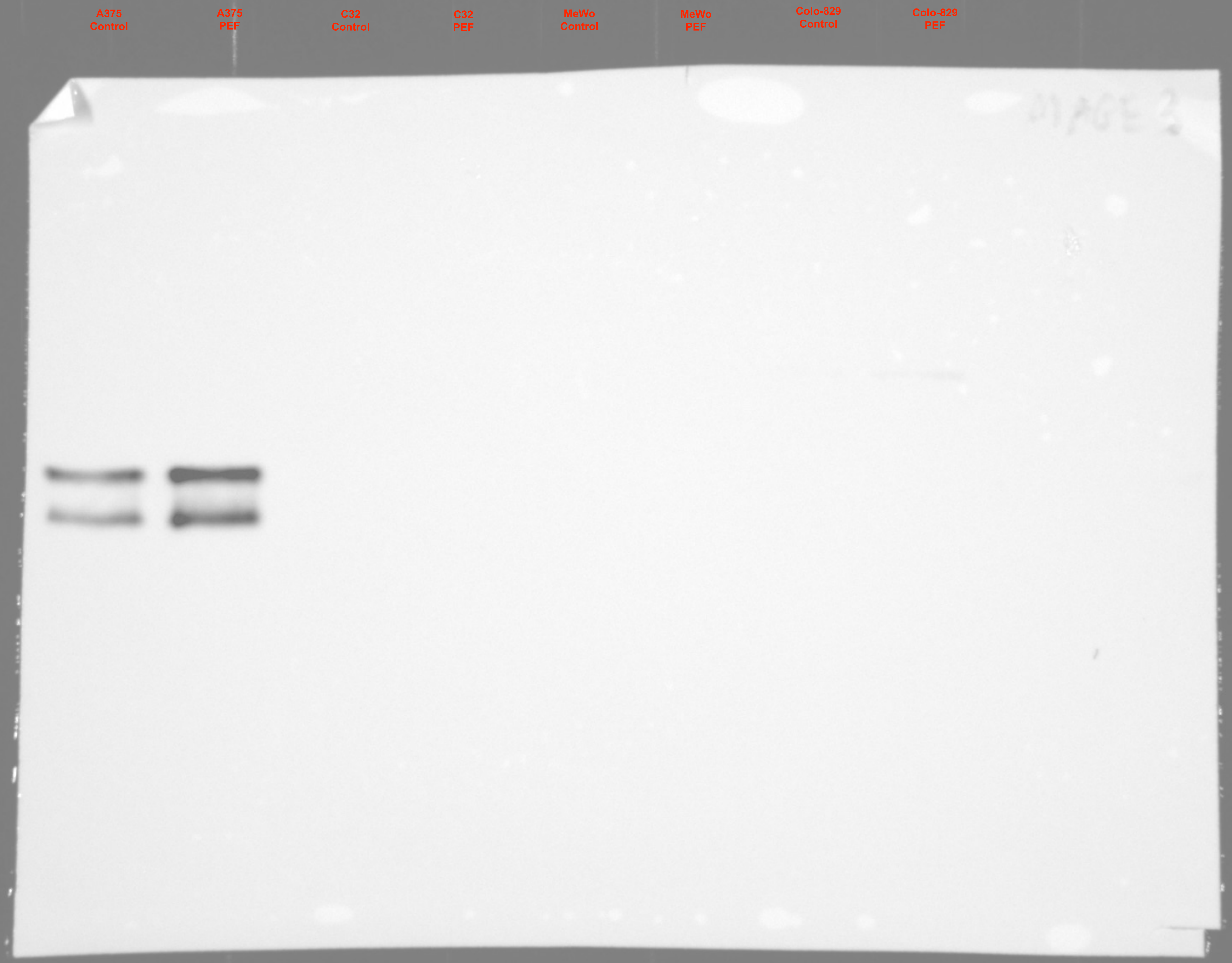
A375 Control
A375 PEF
C32 Control
C32 PEF
MeWo Control
MeWo PEF
Colo-829 Control
Colo-829 PEF
MAGE 2

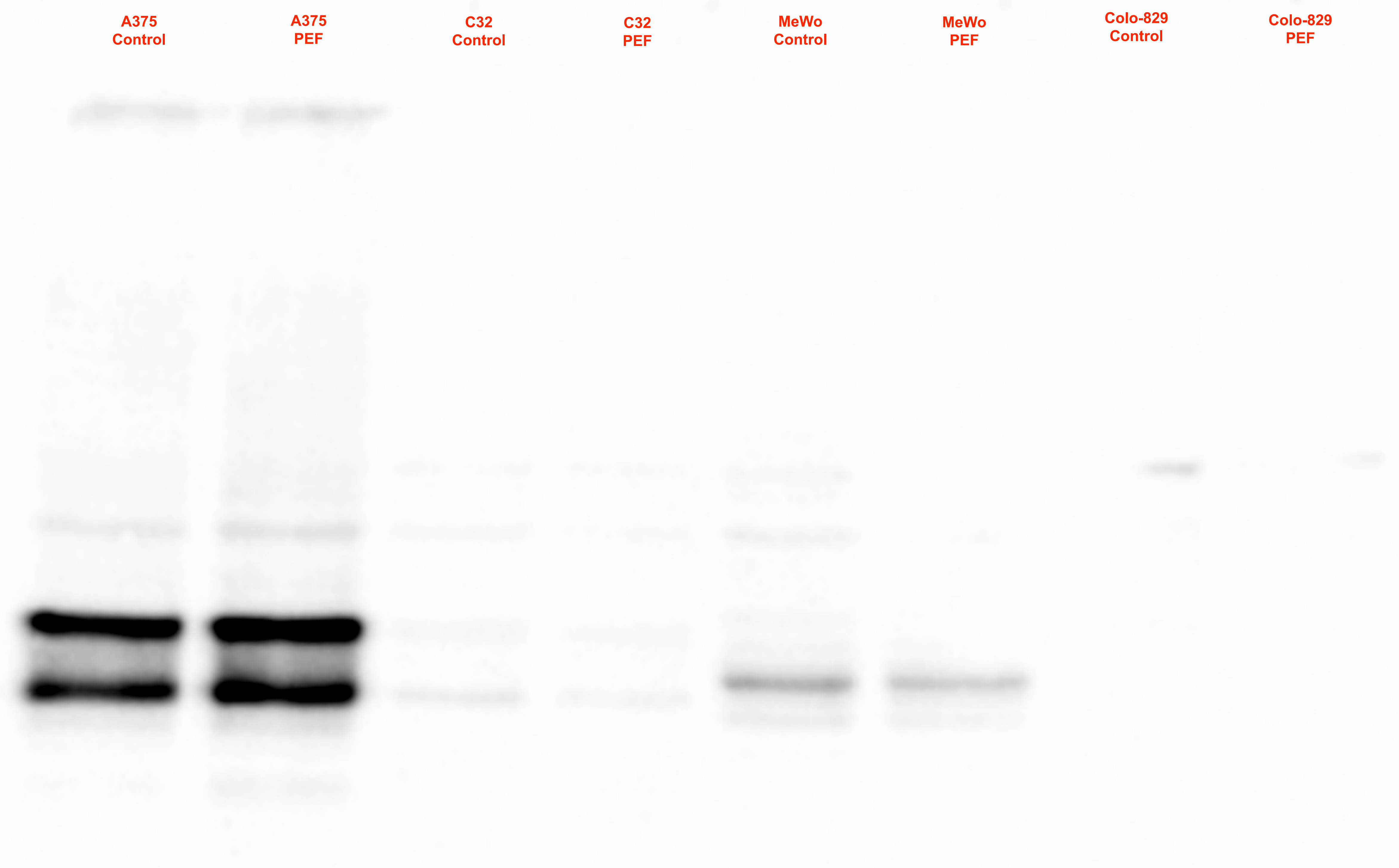
A375 Control
A375 PEF
C32 Control
C32 PEF
MeWo Control
MeWo PEF
Colo-829 Control
Colo-829 PEF

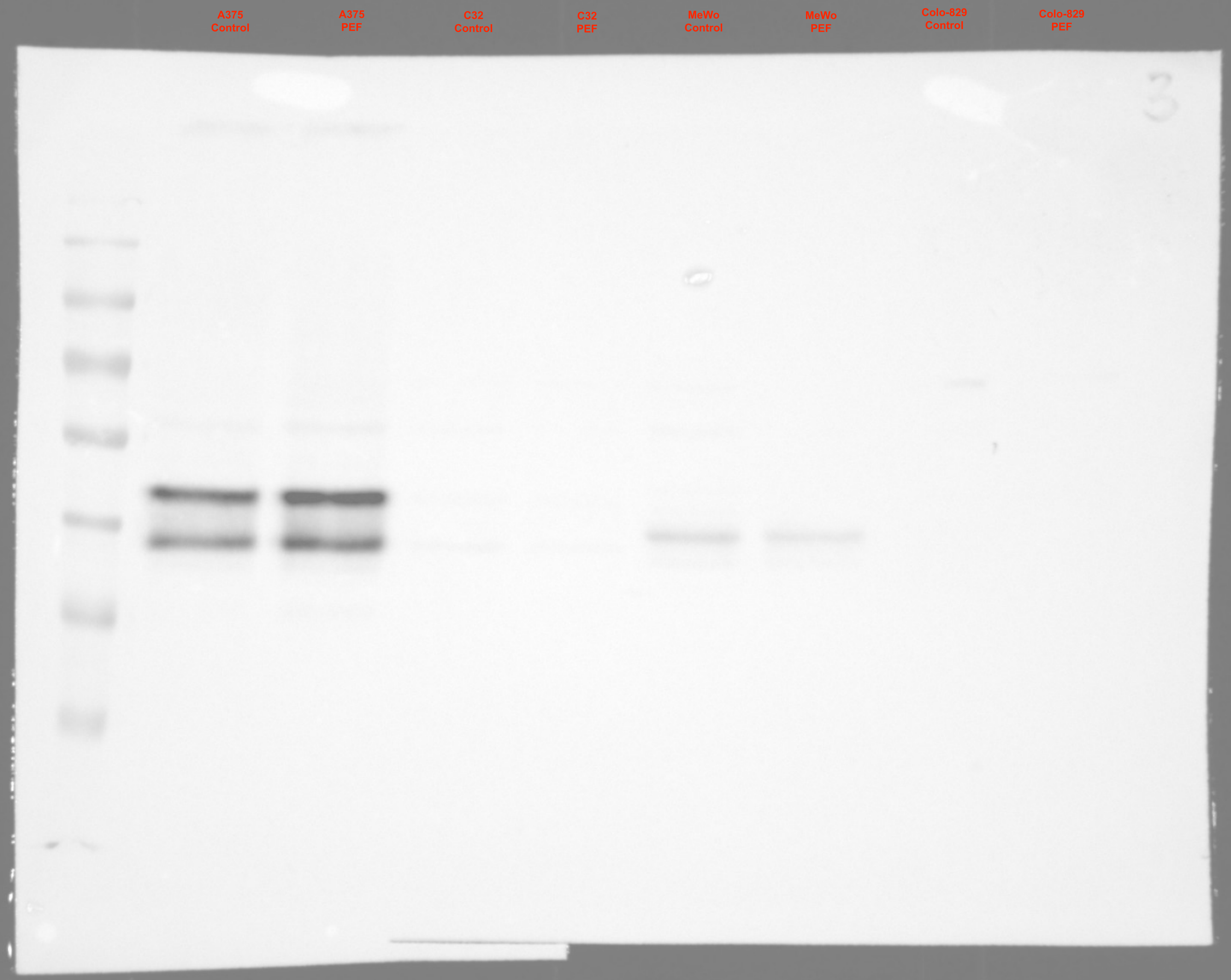
A375 Control
A375 PEF
C32 Control
C32 PEF
MeWo Control
MeWo PEF
Colo-829 Control
Colo-829 PEF

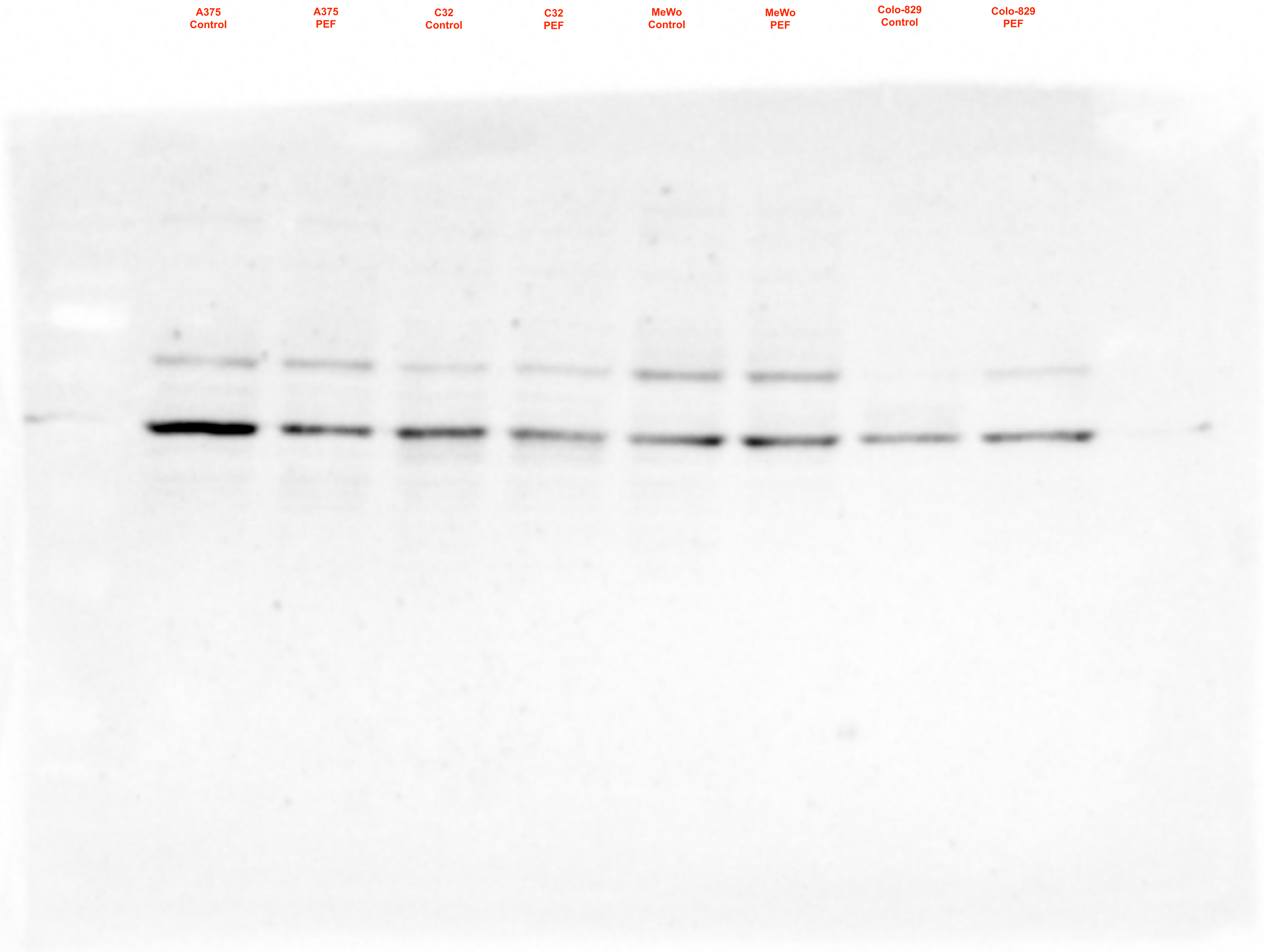

A375 Control
A375 PEF
C32 Control
C32 PEF
MeWo Control
MeWo PEF
Colo-829 Control
Colo-829 PEF

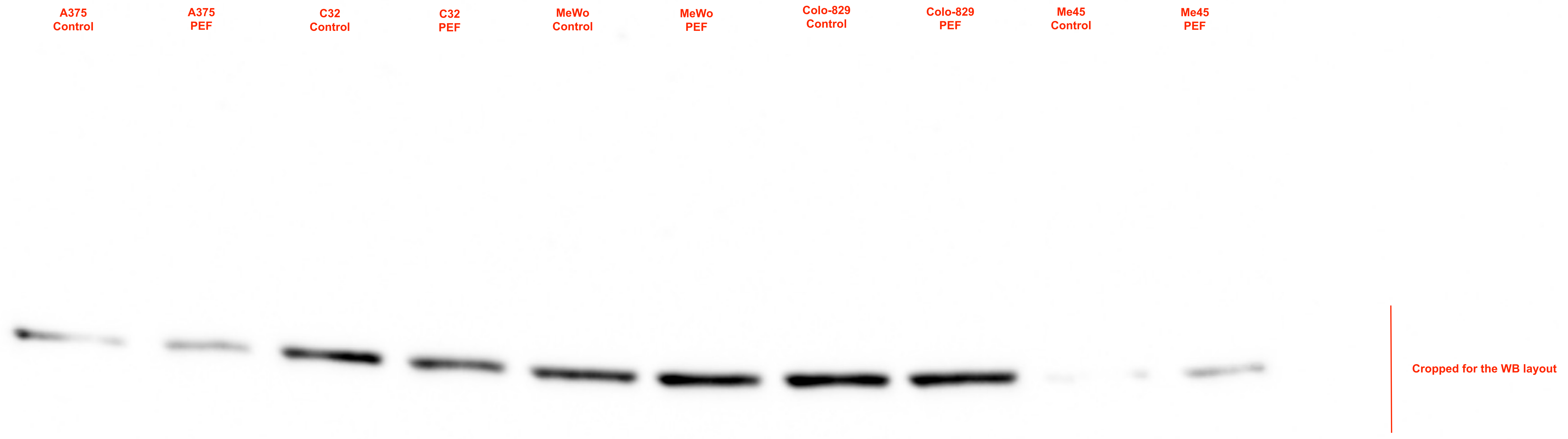
A375 Control
A375 PEF
C32 Control
C32 PEF
MeWo Control
MeWo PEF
Colo-829 Control
Colo-829 PEF
Me45 Control
Me45 PEF
Cropped for the WB layout

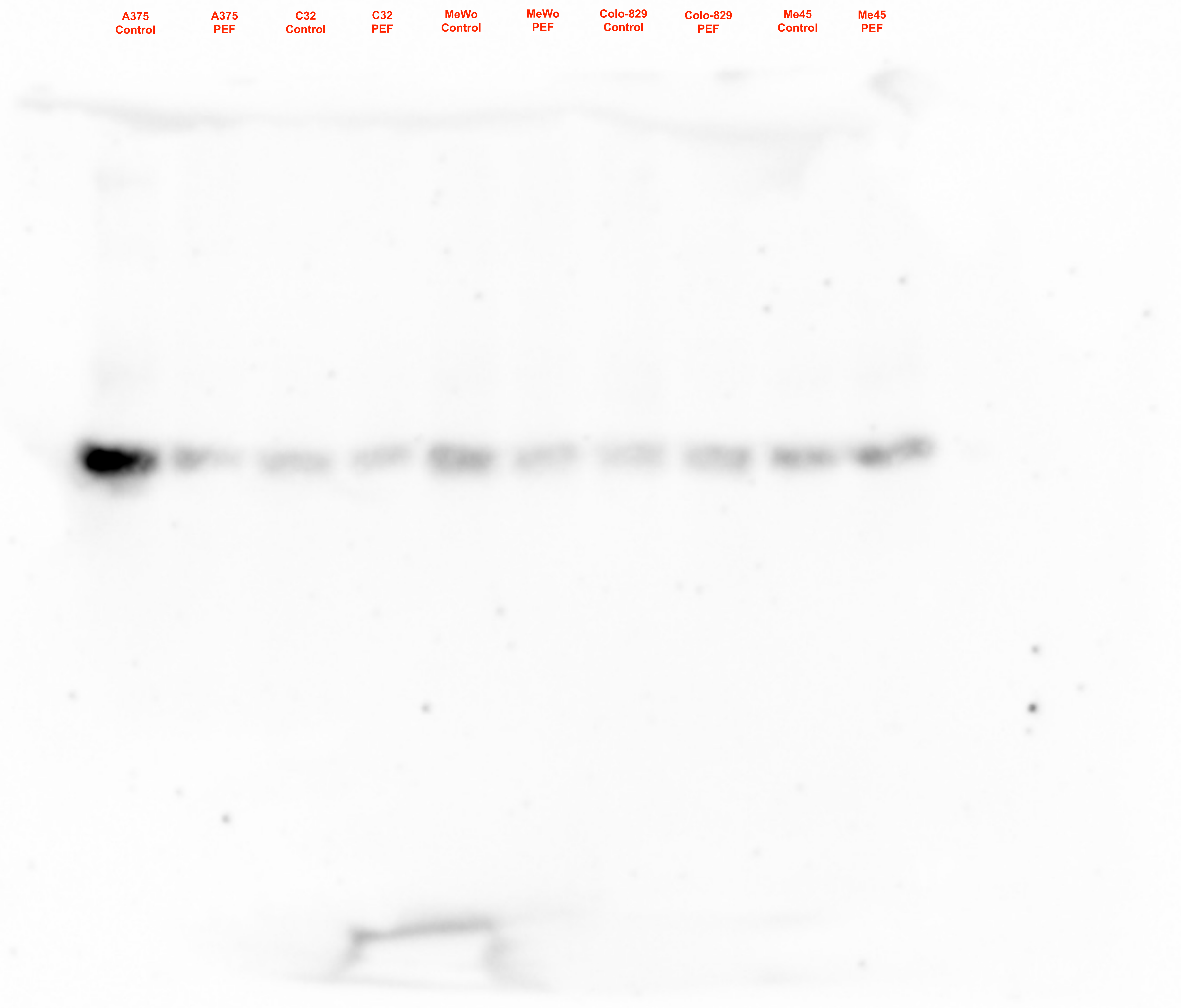
A375 Control
A375 PEF
C32 Control
C32 PEF
MeWo Control
MeWo PEF
Colo-829 Control
Colo-829 PEF
Me45 Control
Me45 PEF

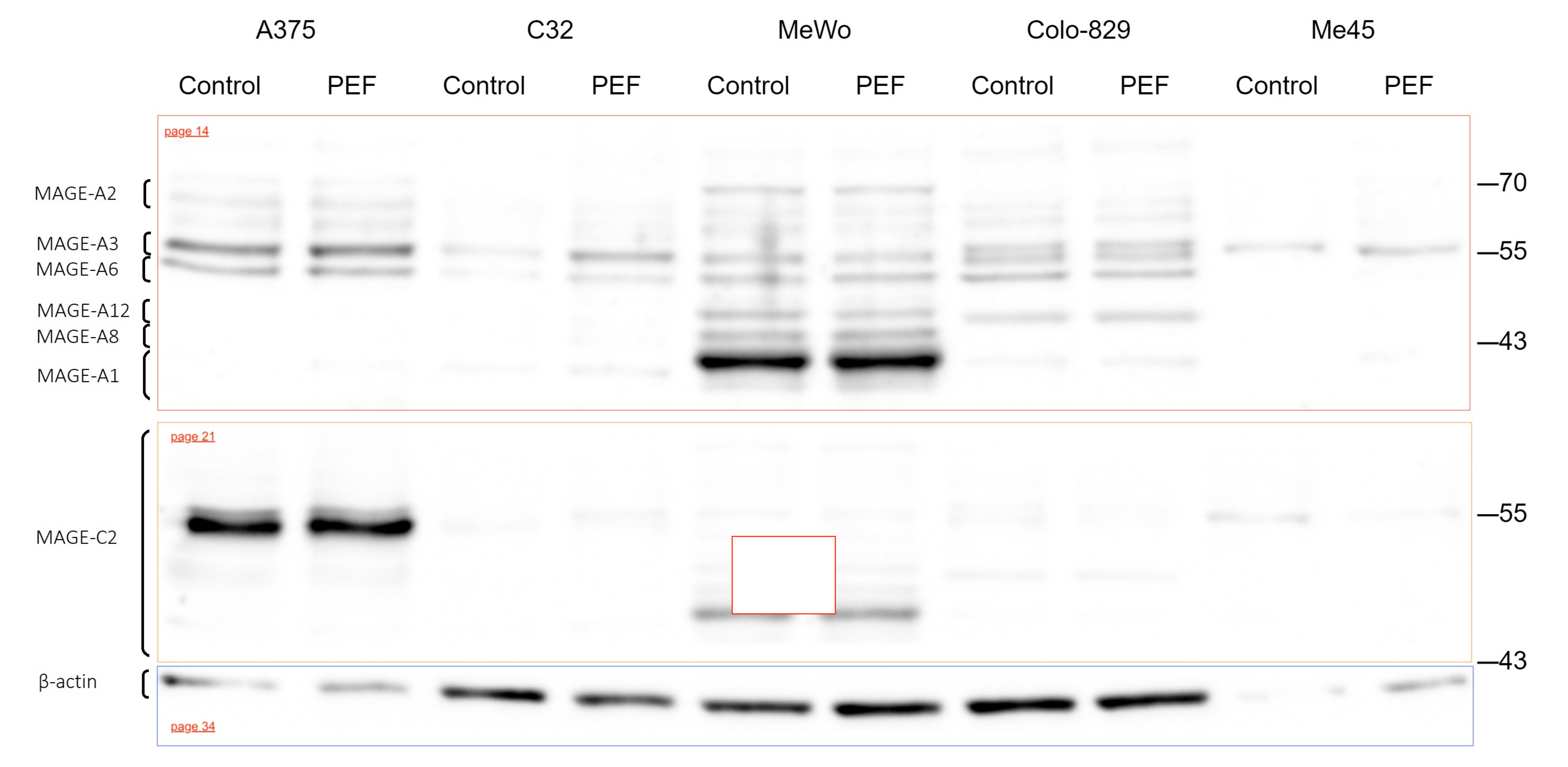

A375
C32
MeWo
Colo-829
Me45
Control
PEF
Control
PEF
Control
PEF
Control
PEF
Control
PEF
page 14
MAGE-A2
MAGE-A3
MAGE-A6
MAGE-A12
MAGE-A8
MAGE-A1
—70
—55
—43
page 21
MAGE-C2
—55
—43
β-actin
page 34

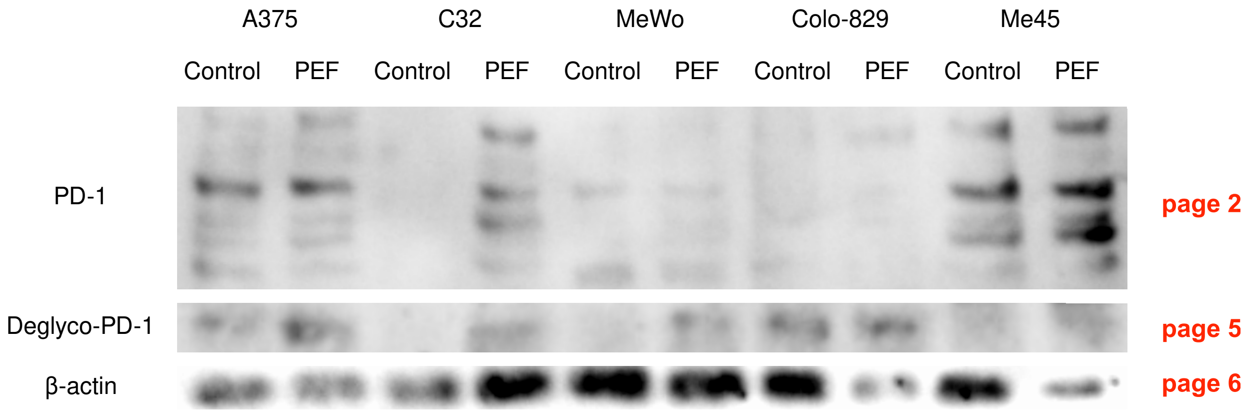

A375
C32
MeWo
Colo-829
Me45
Control
PEF
Control
PEF
Control
PEF
Control
PEF
Control
PEF
PD-1
Deglyco-PD-1
β-actin
page 2
page 5
page 6

Metabolomics and lipidomics studies

Using high-throughput untargeted metabolomics, we compared A375, C32, ME45, MeWo and CaCo-2 cell lines subjected to nanosecond electrical pulses and controls against each other. The analysis revealed 35 hydrophilic/polar identified metabolites. Univariate one-way ANOVA revealed 29 significant hits, with at least two of the ten groups being significantly different (false discovery rate (FDR)-corrected; *p*-value <0.05). Hierarchical clustering analysis represented in the heatmap (Fig. 1.) illustrates clear sample clustering within each of the cell groups. Principal component analysis (PCA) revealed significant separation between the melanomas and CaCo-2 cell lines as was to be thought, furthermore in the melanomas group A375 and C32 showed significantly different metabolic profiles, while ME34 and MeWo showed a similar metabolic profile overlapped with each other and partly with the A375 and C32 melanomas (Fig. 2.). Partial least squares-discriminant analysis (PLS-DA) intensified the clustering (Fig. 3.). Removal of CaCo-2 from the analysis did not affect the separation of melanoma. The PLS-DA variable importance in projection(VIP) score, which ranks metabolites according to their importance for group separation, revealed 7 metabolites with a VIP-score >1 (Fig 4.). However, taking into account the pilot nature of the research, the compounds with VIP scores below 1 were also loaded for the study of metabolic pathways. The following compounds were taken into account for the following analysis: Glutathione, 1-Monostearin, Oleamide, Spermine, Creatine, Pyruvic acid, Cysteinylglycine, Tryptophan, Tyrosine, Valine, Glutamate, Spermidine. Pathway analysis were conducted using Metaboanalyst 5.0 module. Three metabolomic pathways with impact score above 0.3 were revealed, namely: Glutathione metabolism (impact score = 0.34), Phenylalanine, tyrosine and tryptophan biosynthesis (impact score = 0.5), D-Glutamine and D-glutamate metabolism (impact score = 0.5) (Fig. 5.).

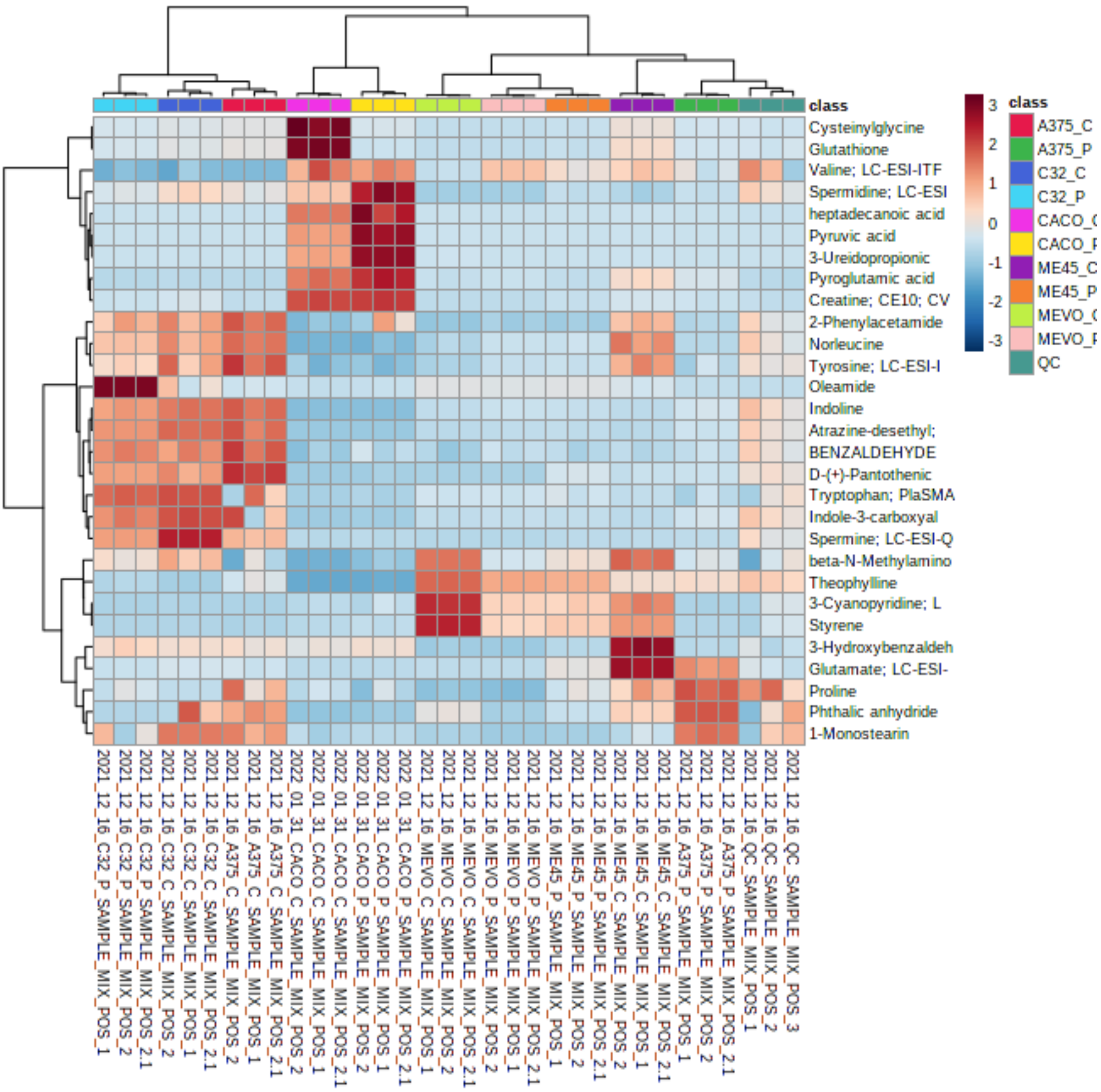
class
Cysteinylglycine
Glutathione
Valine; LC-ESI-ITF
Spermidine; LC-ESI
heptadecanoic acid
Pyruvic acid
3-Ureidopropionic
Pyroglutamic acid
Creatine; CE10; CV
2-Phenylacetamide
Norleucine
Tyrosine; LC-ESI-I
Oleamide
Indoline
Atrazine-desethyl;
BENZALDEHYDE
D-(+)-Pantothenic
Tryptophan; PlaSMA
Indole-3-carboxyal
Spermine; LC-ESI-Q
beta-N-Methylamino
Theophylline
3-Cyanopyridine; L
Styrene
3-Hydroxybenzaldeh
Glutamate; LC-ESI-
Proline
Phthalic anhydride
1-Monostearin
3
2
1
0
-1
-2
-3
class
A375_C
A375_P
C32_C
C32_P
CACO_(
CACO_I
ME45_C
ME45_P
MEVO_(
MEVO_I
QC
2021_12_16_C32_P_SAMPLE_MIX_POS_1
2021_12_16_C32_P_SAMPLE_MIX_POS_2
2021_12_16_C32_P_SAMPLE_MIX_POS_2.1
2021_12_16_C32_C_SAMPLE_MIX_POS_2
2021_12_16_C32_C_SAMPLE_MIX_POS_1
2021_12_16_C32_C_SAMPLE_MIX_POS_2.1
2021_12_16_A375_C_SAMPLE_MIX_POS_2
2021_12_16_A375_C_SAMPLE_MIX_POS_1
2021_12_16_A375_C_SAMPLE_MIX_POS_2.1
2022_01_31_CACO_C_SAMPLE_MIX_POS_2
2022_01_31_CACO_C_SAMPLE_MIX_POS_1
2022_01_31_CACO_C_SAMPLE_MIX_POS_2.1
2022_01_31_CACO_P_SAMPLE_MIX_POS_2
2022_01_31_CACO_P_SAMPLE_MIX_POS_1
2022_01_31_CACO_P_SAMPLE_MIX_POS_2.1
2021_12_16_MEVO_C_SAMPLE_MIX_POS_1
2021_12_16_MEVO_C_SAMPLE_MIX_POS_2
2021_12_16_MEVO_C_SAMPLE_MIX_POS_2.1
2021_12_16_MEVO_P_SAMPLE_MIX_POS_2
2021_12_16_MEVO_P_SAMPLE_MIX_POS_1
2021_12_16_MEVO_P_SAMPLE_MIX_POS_2.1
2021_12_16_ME45_P_SAMPLE_MIX_POS_1
2021_12_16_ME45_P_SAMPLE_MIX_POS_2
2021_12_16_ME45_P_SAMPLE_MIX_POS_2.1
2021_12_16_ME45_C_SAMPLE_MIX_POS_2
2021_12_16_ME45_C_SAMPLE_MIX_POS_1
2021_12_16_ME45_C_SAMPLE_MIX_POS_2.1
2021_12_16_A375_P_SAMPLE_MIX_POS_1
2021_12_16_A375_P_SAMPLE_MIX_POS_2
2021_12_16_A375_P_SAMPLE_MIX_POS_2.1
2021_12_16_QC_SAMPLE_MIX_POS_1
2021_12_16_QC_SAMPLE_MIX_POS_2
2021_12_16_QC_SAMPLE_MIX_POS_3

A

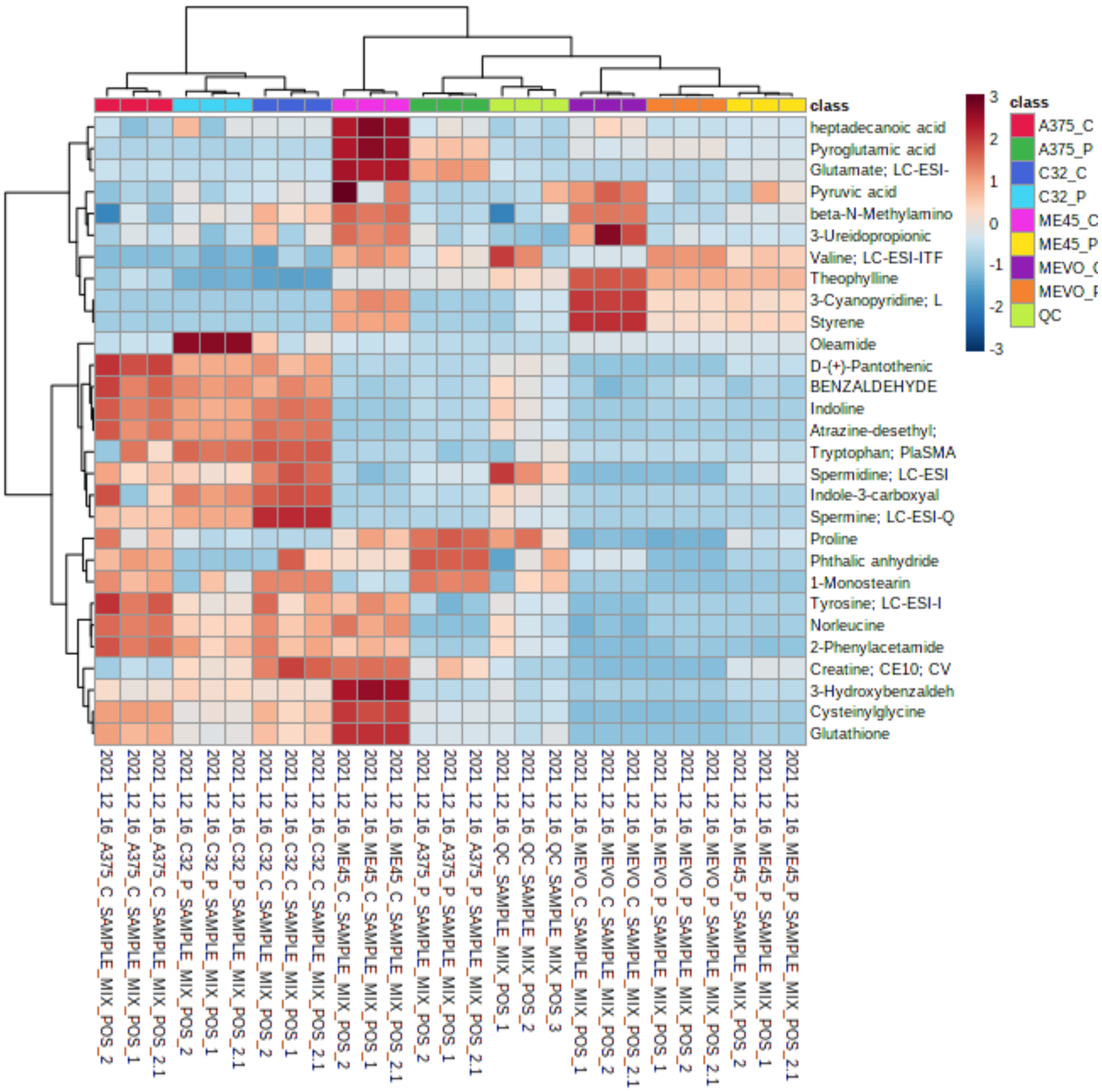

B

Fig. 1 Clustering result shown as heatmap (A) A375, C32, ME45, MeWo and CaCo-2 cell lines (B)) A375, C32, ME45, MeWo cell lines. Distance measure using euclidean, and clustering algorithm using ward.D.

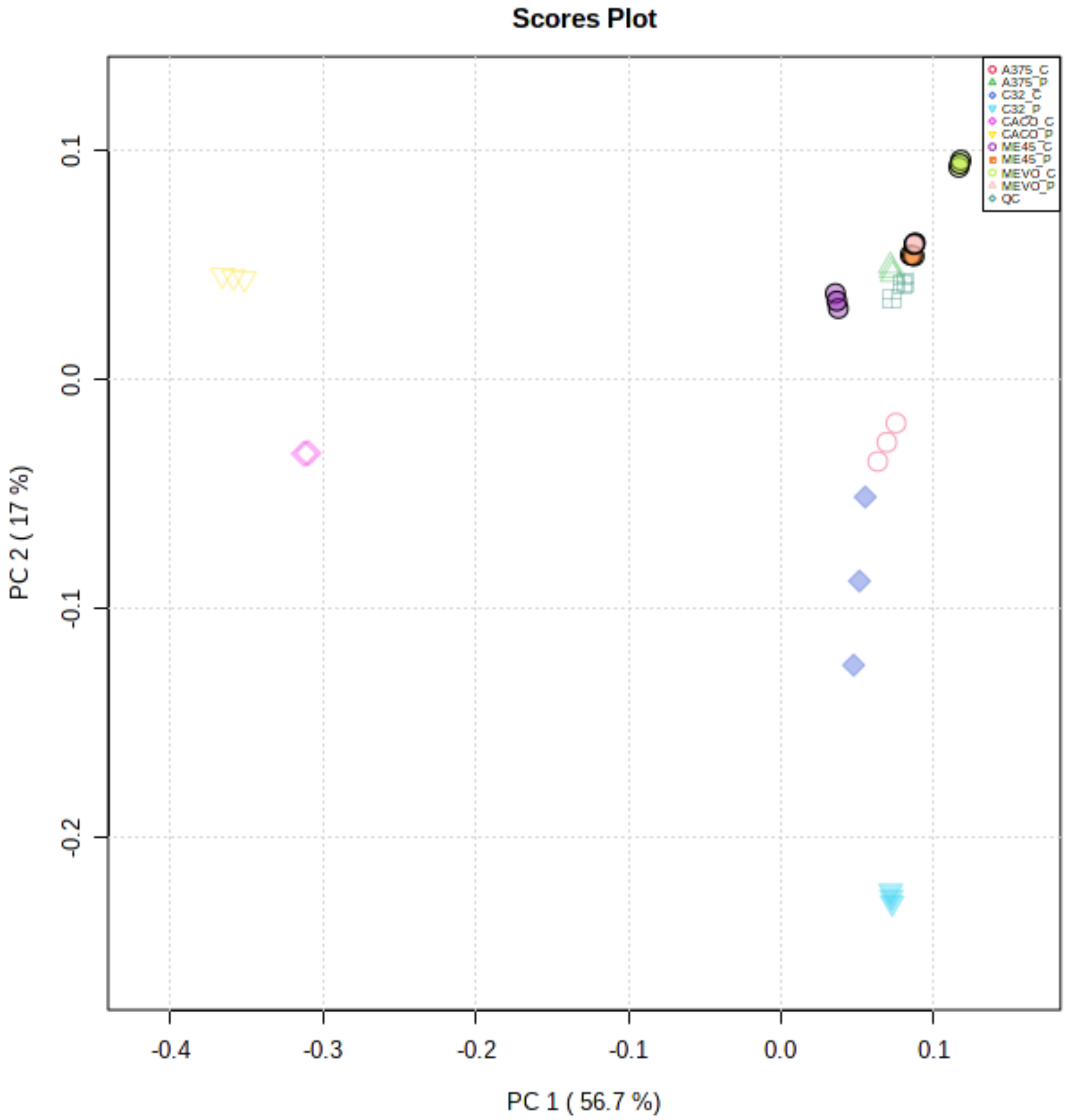
Scores Plot
A375_C
A375_P
C32_C
C32_P
CACO_C
CACO_P
ME45_C
ME45_P
MEVO_C
MEVO_P
QC
0.1
0.0
-0.1
-0.2
PC 2 (17 %)
-0.4
-0.3
-0.2
-0.1
0.0
0.1
PC 1 (56.7 %)

A

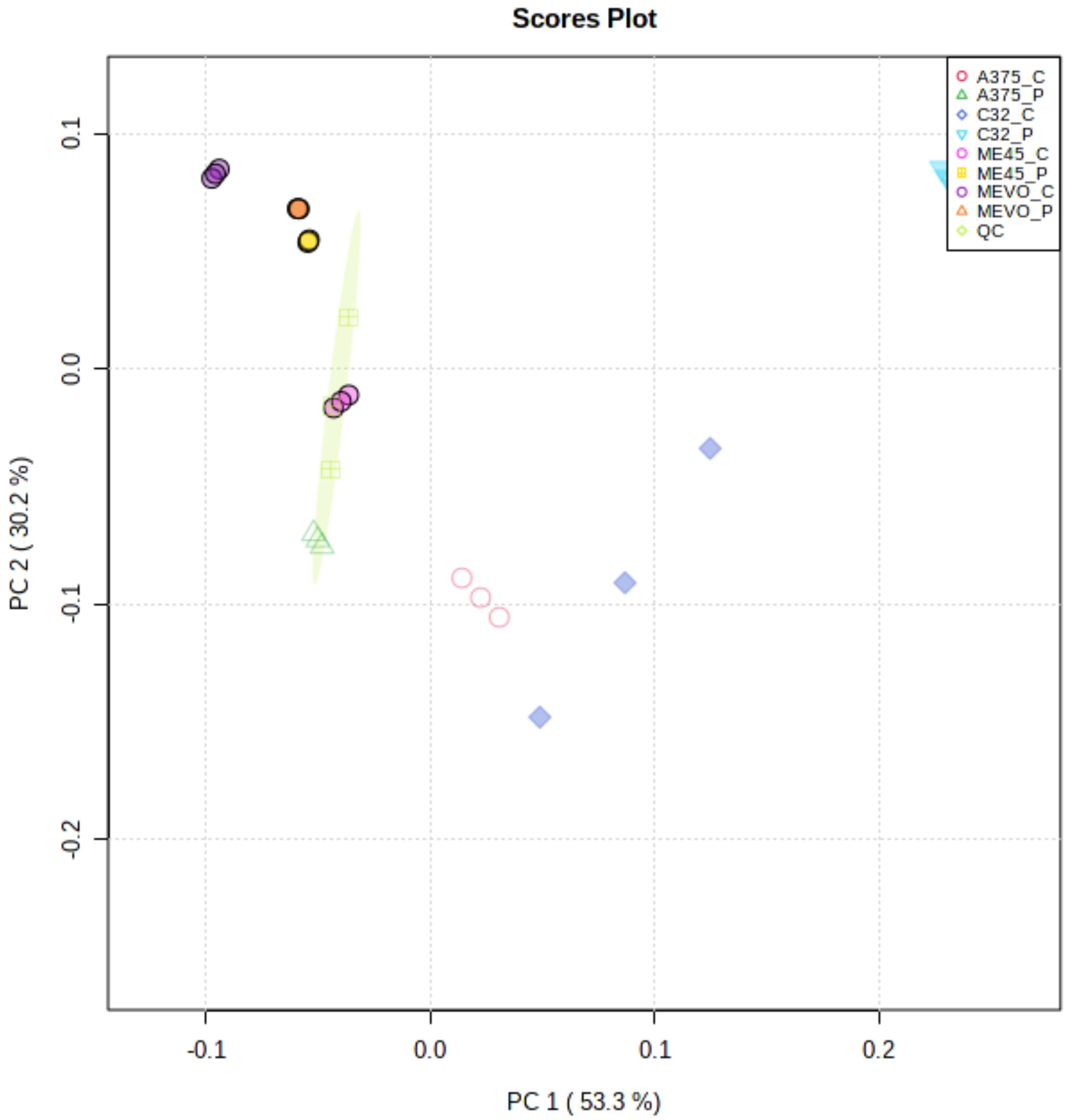

B

Fig. 2. Principal component analysis (PCA) scores (A) A375, C32, ME45, MeWo and CaCo-2 cell lines (B)) A375, C32, ME45, MeWo cell lines. Scores plot between the selected PCs. The explained variances are shown in brackets.

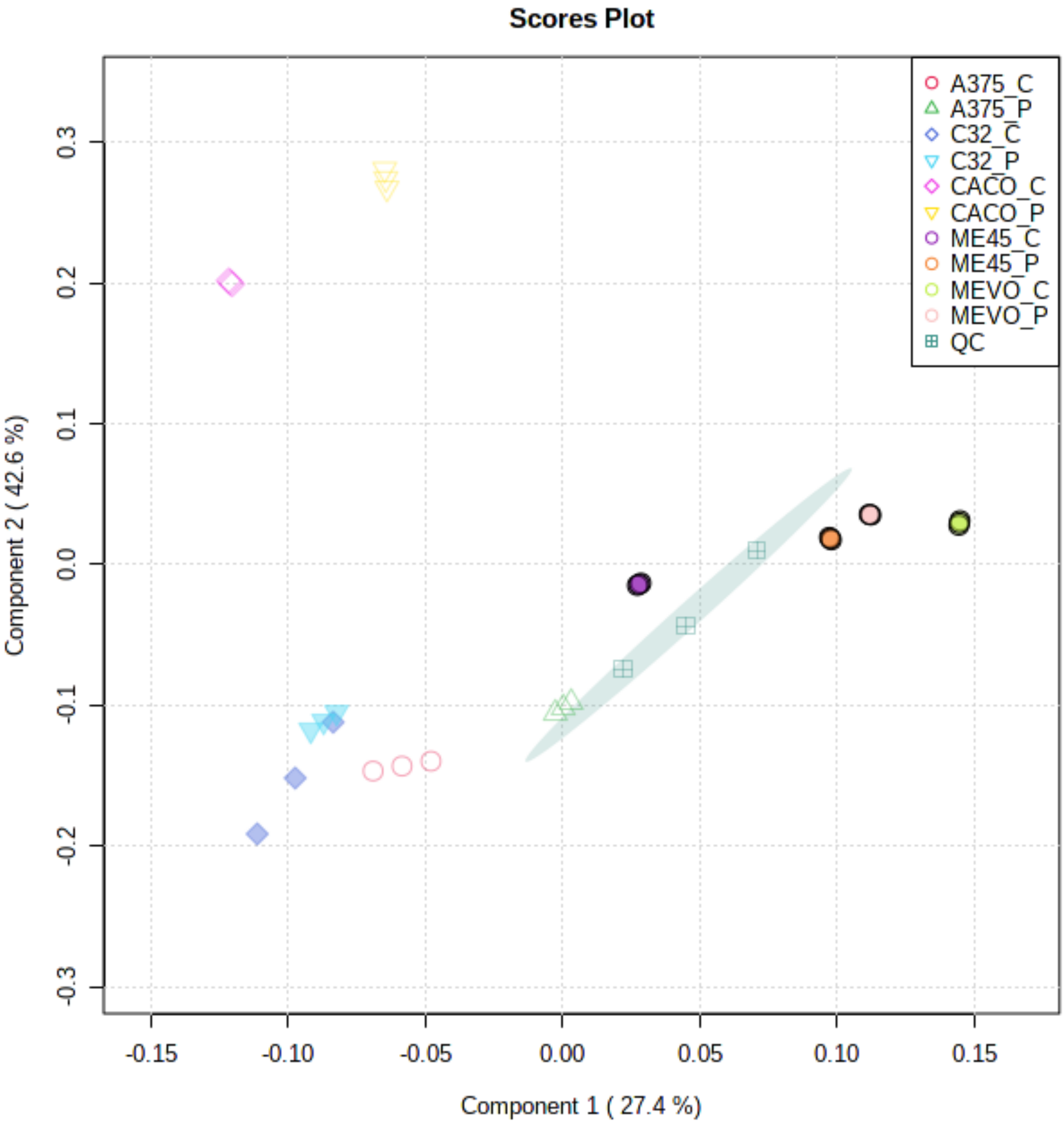

Scores Plot
A375_C
A375_P
C32_C
C32_P
CACO_C
CACO_P
ME45_C
ME45_P
MEVO_C
MEVO_P
QC
0.3
0.2
0.1
0.0
-0.1
-0.2
-0.3
Component 2 (42.6 %)
-0.15
-0.10
-0.05
0.00
0.05
0.10
0.15
Component 1 (27.4 %)

A

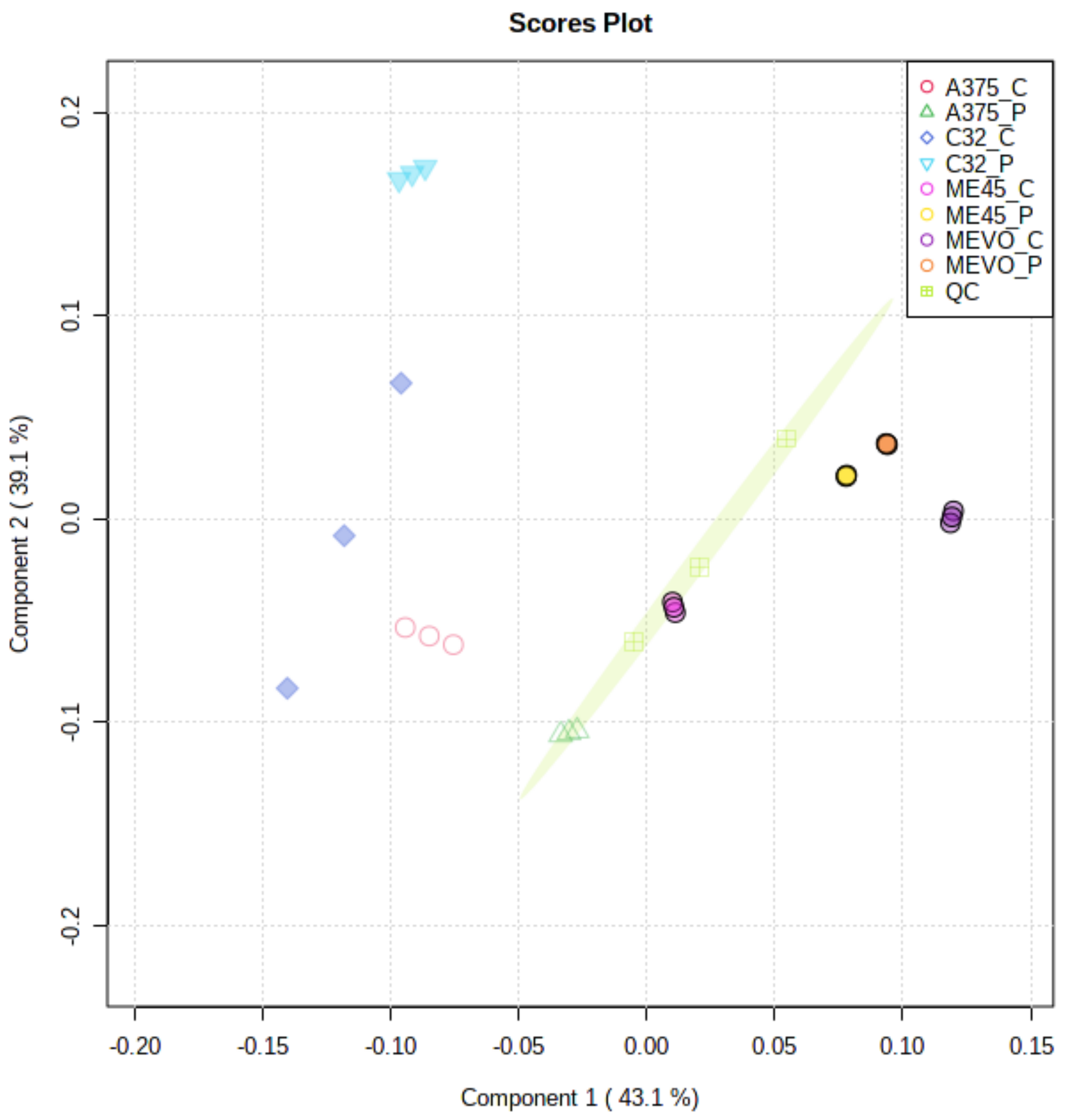

B

Fig. 3. Partial least squares-discriminant analysis (PLS-DA) scores (A) A375, C32, ME45, MeWo and CaCo-2 cell lines (B)) A375, C32, ME45, MeWo cell lines. Scores plot between the selected PCs. The explained variances are shown in brackets.

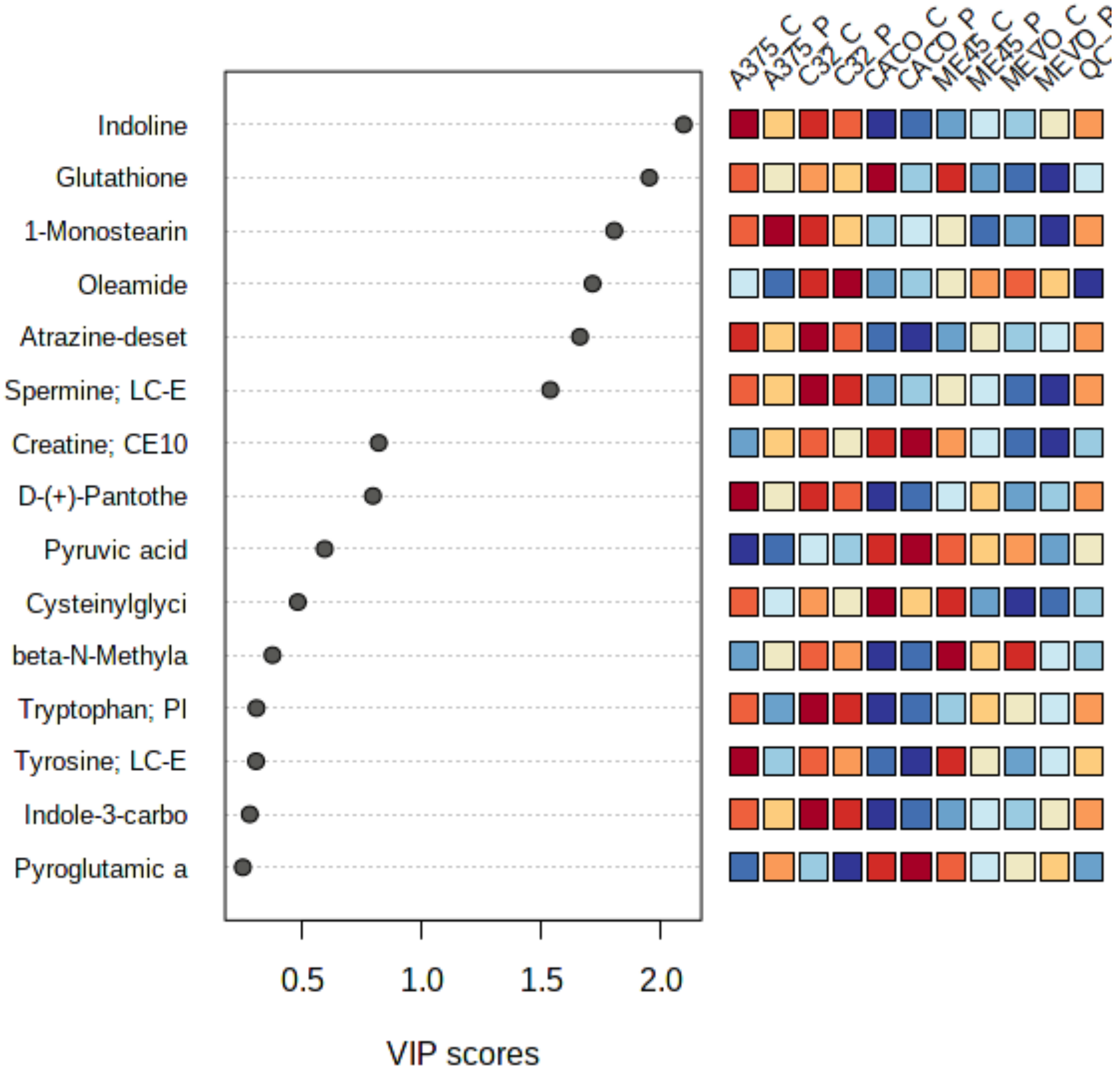

Fig. 4. PLS-DA variable importance in projection (VIP) score derived from the hydrophilic/polar metabolite profiling data of C32, ME45, MeWo and CaCo-2. The colored boxes on the right indicate the relative concentrations of the corresponding metabolite in each group under study.

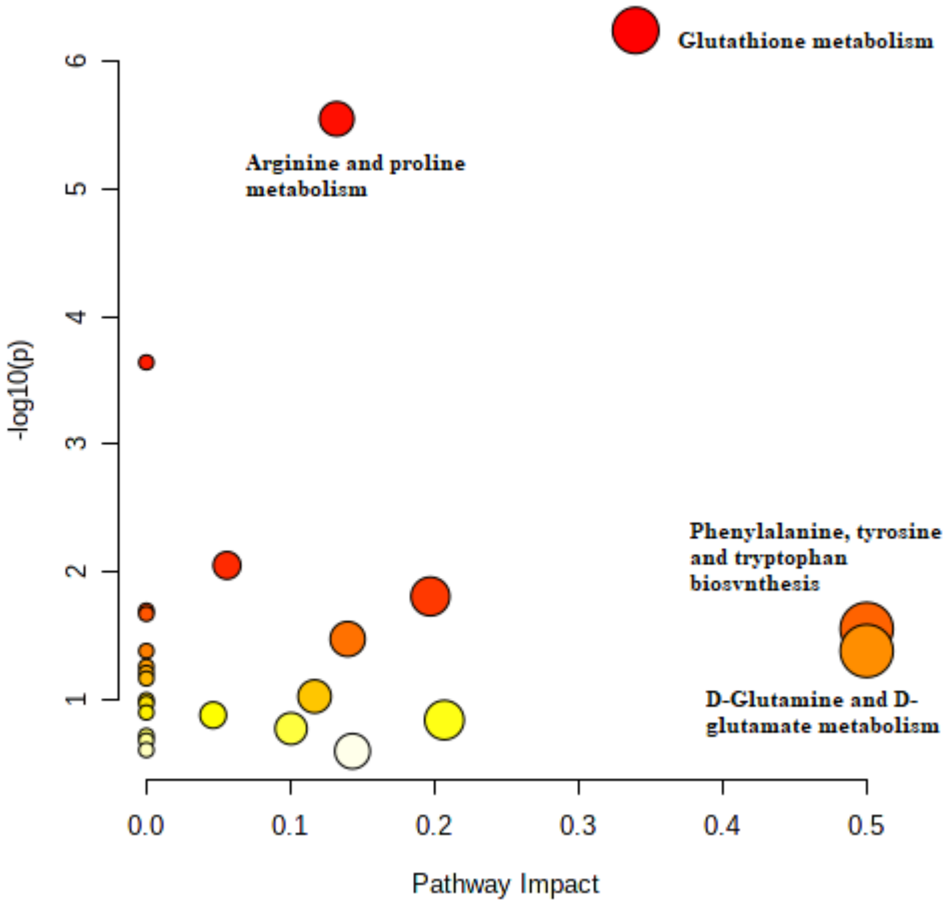

Fig. 5. Metabolic pathway analysis of selected metabolites. The metabolome view shows matched pathways arranged by p-values from pathway enrichment analysis (Y-axis) and pathway impact values from pathway topology analysis (X-axis). Node color and radius are based on the p-value and pathway impact value, respectively.

Lipidomic untargeted analysis were conducted in positive and negative ionization moce, A375, C32, ME45, MeWo and CaCo cell lines subjected to nanosecond electrical pulses and controls were compared against each other. The analysis idnetified 2106 and 320 lipids and lipid-like metabolites in positive and negative ionization modes, respectivly . For further analysis 100 lipid species with highest intensity from each MS lipid analysis (positive/negative) were chosen. Positve charge lipids consisted of 14% SM species, 13% Cer species, 12% NAG species, 9% FA, 8% DG species, 7% VAE, 7% TG, 6% NAE, 6% SL, 4% PC, 3% PS, 2% PI, 2% SPB, 1% each of PG, PE, ST, and BMP. Negative charge lipids consited of 36% PE species, 14% Cer species, 14% FA species, 11% PC species, 7% PG species, 5% PI, 4% PS, 2% SM, 2% CL, 2% LPE, and 2% LPG.

One-way ANOVA resulted in 200 (all selected lipids) significant hits, with at least two of the four groups being significantly different (FDR-corrected; *p*-value <0.05). The results of the hierarchical clustering analysis (heatmap) illustrate a good group separation between the four

melanomas in both positive and negative groups and joined group (positive plus negative) (Fig 6.).

Principal component analysis (PCA) showed that CaCo-2 is significantly separated from the melanoma cell lines groups(for both caseswith postive and naegative charge). In contrast, PCA as well as PLS-DA showed also good group separation between the melanomas (Fig. 7 and Fig. 8).

In positive charge lipids species groups, A375, C32, Me45 and MeWo form clusters. In addition, the A375 and C32 melanoma group overlap with each other and partially with Me45. Partial least squares-discriminant analysis (PLS-DA) intensified cluster formation. The PLS-DA variable importance in projection (VIP) score which ranks metabolites according to their importance for group separation revealed 15 metabolites with VIP>1 score (Fig. 9.). The following compounds have VIP score above 1: TG 25:1_18:2_24:6, HexCer 17:3;3O/13:1;(2OH), DG 47:8, NAGly 19:1;O(FA 19:4), SL 15:3;O/22:6;O, NAGlySer 16:4;O, Carnitine, Tyrosine, VAE 17: 2, VAE 24:6, SL 17:3;O/18:2, Cer 22:2;2O/24:4;O, NAGly 16:3;O(FA 17:4), SM 21:2;2O/9:0, SM 17:1;2O/14:1, VAE 12:0, Cer 21:3;2O/18:5, SM 28:2;3O, NAGlySer 21:0;O, DG 19:0_18:1.

In negative charge lipids species groups, in PCA analysis Me45 and MeWo are significantly separated from the other melanomas. In addition, the A375 and C32 melanoma groups overlap with each other. Partial least squares-discriminant analysis (PLS-DA) intensified cluster formation for all melanoma groups. The PLS-DA variable importance in projection (VIP) score revealed the following 10 metabolites with VIP>1 score (Fig. 9.): PC 15:2_38:2, HexCer 18:1;2O/34:3, PC O-20:3_12:0;2O, HexCer 20:1;2O/30:1;O, FA 22:5, LPG O-14:0, FA 42:5, AAHFA 6:0/15:2;O, PE 18:1_19:1, Cer 14:1;2O/36:5.

Using high-throughput untargeted metabolomics, we compared A375, C32, ME45, MeWo and CaCo cell lines subjected to nanosecond electrical pulses and controls

The largest differences in lipidome between the group subjected to nanosecond electrical pulses and controls were found for the ME and MeWo cell lines, however, it should be mentioned that the differences were caused mainly by negatively charged lipids species.

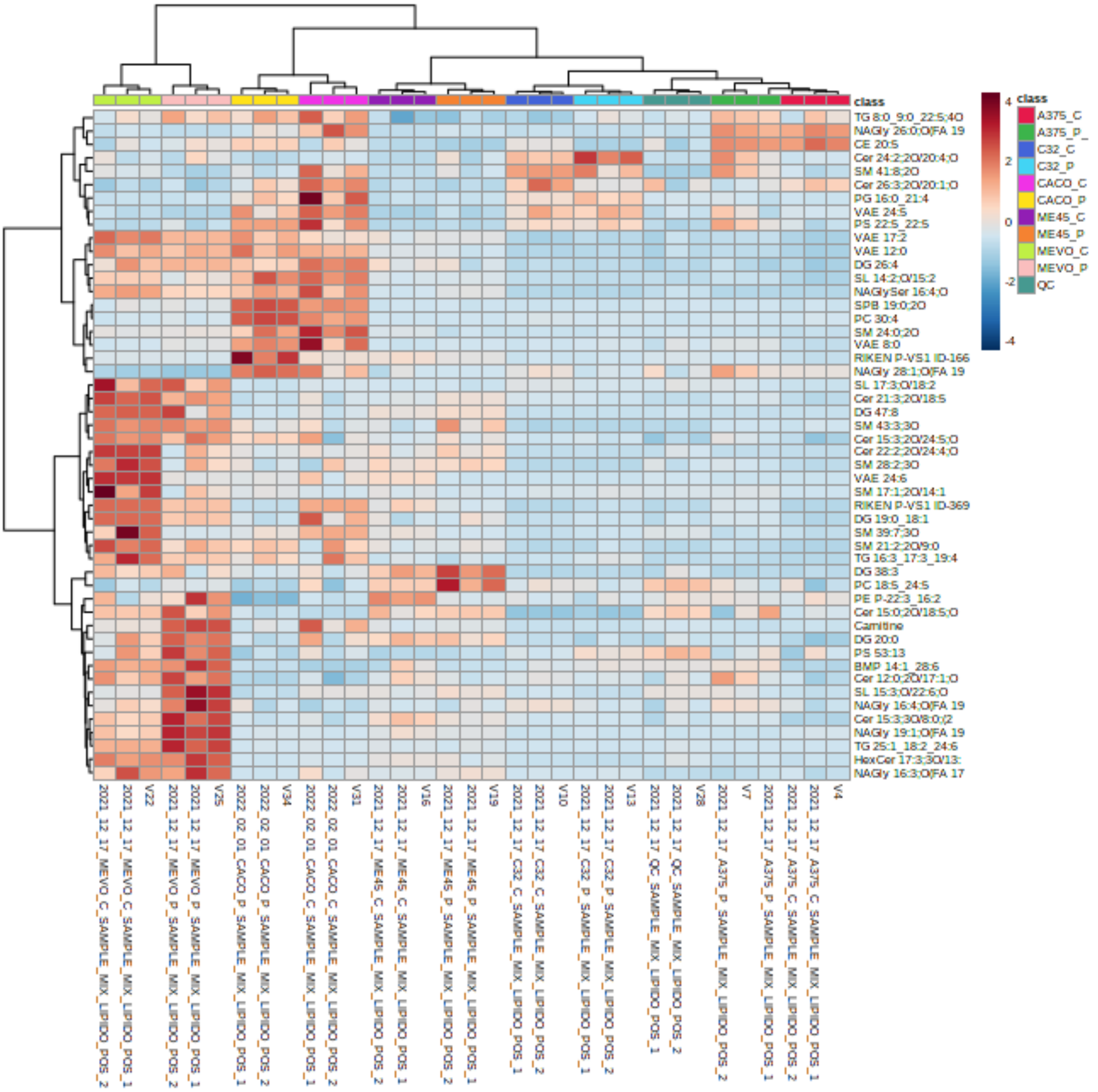
class
TG 8:0_9:0_22:5;4O
NAGly 26:0;O/FA 19
CE 20:5
Cer 24:2;2O/20:4;O
SM 41:8;2O
Cer 26:3;2O/20:1;O
PG 16:0_21:4
VAE 24:5
PS 22:5_22:5
VAE 17:2
VAE 12:0
DG 26:4
SL 14:2;O/15:2
NAGlySer 16:4;O
SPB 19:0;2O
PC 30:4
SM 24:0;2O
VAE 8:0
RIKEN P-VS1 ID-166
NAGly 28:1;O/FA 19
SL 17:3;O/18:2
Cer 21:3;2O/18:5
DG 47:8
SM 43:3;3O
Cer 15:3;2O/24:5;O
Cer 22:2;2O/24:4;O
SM 28:2;3O
VAE 24:6
SM 17:1;2O/14:1
RIKEN P-VS1 ID-369
DG 19:0_18:1
SM 39:7;3O
SM 21:2;2O/9:0
TG 16:3_17:3_19:4
DG 38:3
PC 18:5_24:5
PE P-22:3_16:2
Cer 15:0;2O/18:5;O
Carnitine
DG 20:0
PS 53:13
BMP 14:1_28:6
Cer 12:0;2O/17:1;O
SL 15:3;O/22:6;O
NAGly 16:4;O/FA 19
Cer 15:3;3O/8:0;(2
NAGly 19:1;O/FA 19
TG 25:1_18:2_24:6
HexCer 17:3;3O/13:
NAGly 16:3;O/FA 17
2021_12_17_MEVO_C_SAMPLE_MIX_LIPIDO_POS_2
2021_12_17_MEVO_C_SAMPLE_MIX_LIPIDO_POS_1
V22
2021_12_17_MEVO_P_SAMPLE_MIX_LIPIDO_POS_2
2021_12_17_MEVO_P_SAMPLE_MIX_LIPIDO_POS_1
V25
2022_02_01_CACO_P_SAMPLE_MIX_LIPIDO_POS_1
2022_02_01_CACO_P_SAMPLE_MIX_LIPIDO_POS_2
V34
2022_02_01_CACO_C_SAMPLE_MIX_LIPIDO_POS_1
2022_02_01_CACO_C_SAMPLE_MIX_LIPIDO_POS_2
V31
2021_12_17_ME45_C_SAMPLE_MIX_LIPIDO_POS_2
2021_12_17_ME45_C_SAMPLE_MIX_LIPIDO_POS_1
V16
2021_12_17_ME45_P_SAMPLE_MIX_LIPIDO_POS_2
2021_12_17_ME45_P_SAMPLE_MIX_LIPIDO_POS_1
V19
2021_12_17_C32_C_SAMPLE_MIX_LIPIDO_POS_1
2021_12_17_C32_C_SAMPLE_MIX_LIPIDO_POS_2
V10
2021_12_17_C32_P_SAMPLE_MIX_LIPIDO_POS_1
2021_12_17_C32_P_SAMPLE_MIX_LIPIDO_POS_2
V13
2021_12_17_QC_SAMPLE_MIX_LIPIDO_POS_1
2021_12_17_QC_SAMPLE_MIX_LIPIDO_POS_2
V28
2021_12_17_A375_P_SAMPLE_MIX_LIPIDO_POS_2
V7
2021_12_17_A375_P_SAMPLE_MIX_LIPIDO_POS_1
2021_12_17_A375_C_SAMPLE_MIX_LIPIDO_POS_2
2021_12_17_A375_C_SAMPLE_MIX_LIPIDO_POS_1
V4
4
2
0
-2
-4
class
A375_C
A375_P_
C32_C
C32_P
CACO_C
CACO_P
ME45_C
ME45_P
MEVO_C
MEVO_P
QC

A

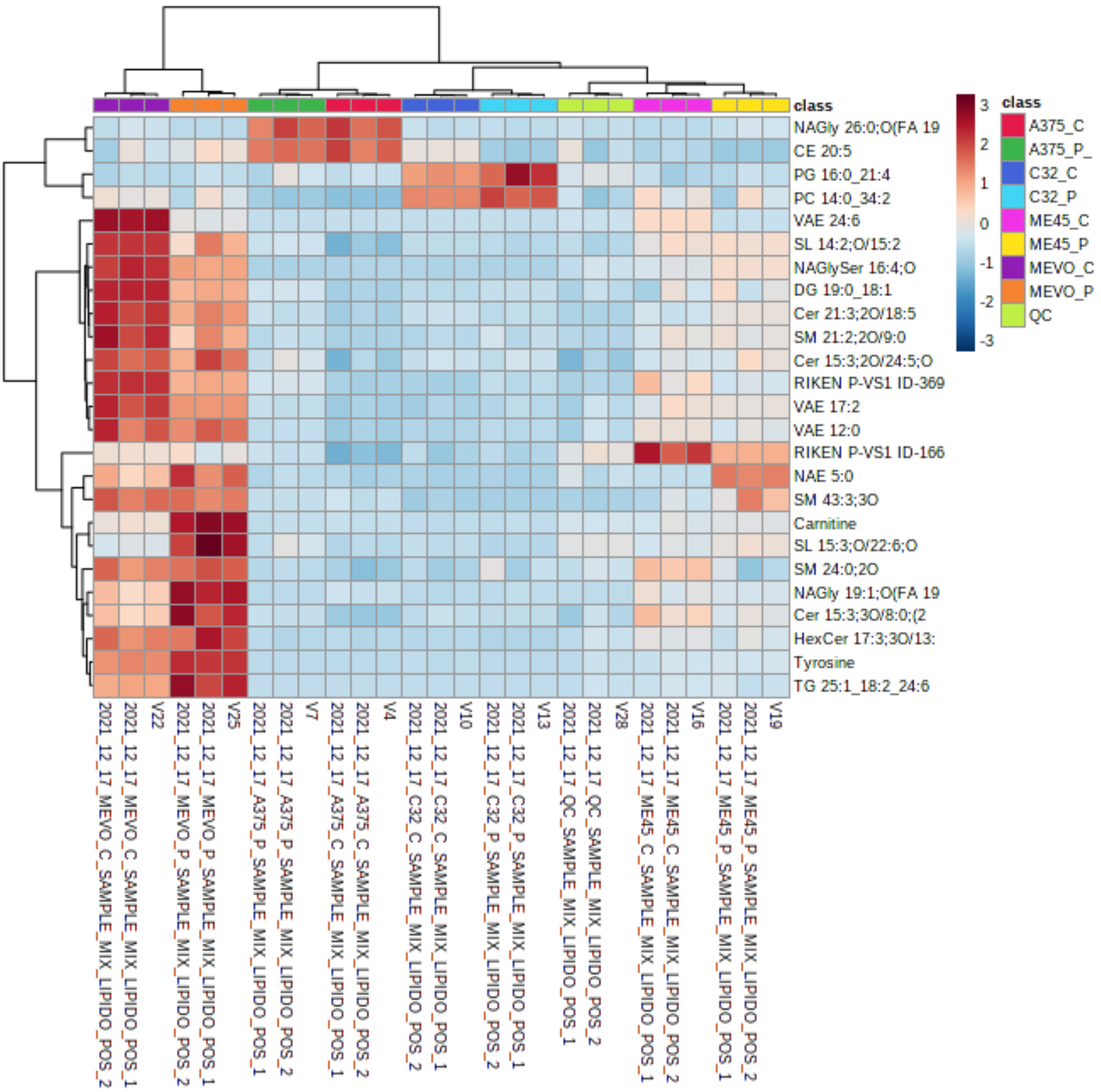
class
NAGly 26:0;O(FA 19
CE 20:5
PG 16:0_21:4
PC 14:0_34:2
VAE 24:6
SL 14:2;O/15:2
NAGlySer 16:4;O
DG 19:0_18:1
Cer 21:3;2O/18:5
SM 21:2;2O/9:0
Cer 15:3;2O/24:5;O
RIKEN P-VS1 ID-369
VAE 17:2
VAE 12:0
RIKEN P-VS1 ID-166
NAE 5:0
SM 43:3;3O
Carnitine
SL 15:3;O/22:6;O
SM 24:0;2O
NAGly 19:1;O(FA 19
Cer 15:3;3O/8:0;(2
HexCer 17:3;3O/13:
Tyrosine
TG 25:1_18:2_24:6
2021_12_17_MEVO_C_SAMPLE_MIX_LIPIDO_POS_2
2021_12_17_MEVO_C_SAMPLE_MIX_LIPIDO_POS_1
V22
2021_12_17_MEVO_P_SAMPLE_MIX_LIPIDO_POS_2
2021_12_17_MEVO_P_SAMPLE_MIX_LIPIDO_POS_1
V25
2021_12_17_A375_P_SAMPLE_MIX_LIPIDO_POS_1
2021_12_17_A375_P_SAMPLE_MIX_LIPIDO_POS_2
V7
2021_12_17_A375_C_SAMPLE_MIX_LIPIDO_POS_1
2021_12_17_A375_C_SAMPLE_MIX_LIPIDO_POS_2
V4
2021_12_17_C32_C_SAMPLE_MIX_LIPIDO_POS_2
2021_12_17_C32_C_SAMPLE_MIX_LIPIDO_POS_1
V10
2021_12_17_C32_P_SAMPLE_MIX_LIPIDO_POS_2
2021_12_17_C32_P_SAMPLE_MIX_LIPIDO_POS_1
V13
2021_12_17_QC_SAMPLE_MIX_LIPIDO_POS_1
2021_12_17_QC_SAMPLE_MIX_LIPIDO_POS_2
V28
2021_12_17_ME45_C_SAMPLE_MIX_LIPIDO_POS_1
2021_12_17_ME45_C_SAMPLE_MIX_LIPIDO_POS_2
V16
2021_12_17_ME45_P_SAMPLE_MIX_LIPIDO_POS_1
2021_12_17_ME45_P_SAMPLE_MIX_LIPIDO_POS_2
V19
3
2
1
0
-1
-2
-3
class
A375_C
A375_P_
C32_C
C32_P
ME45_C
ME45_P
MEVO_C
MEVO_P
QC

B

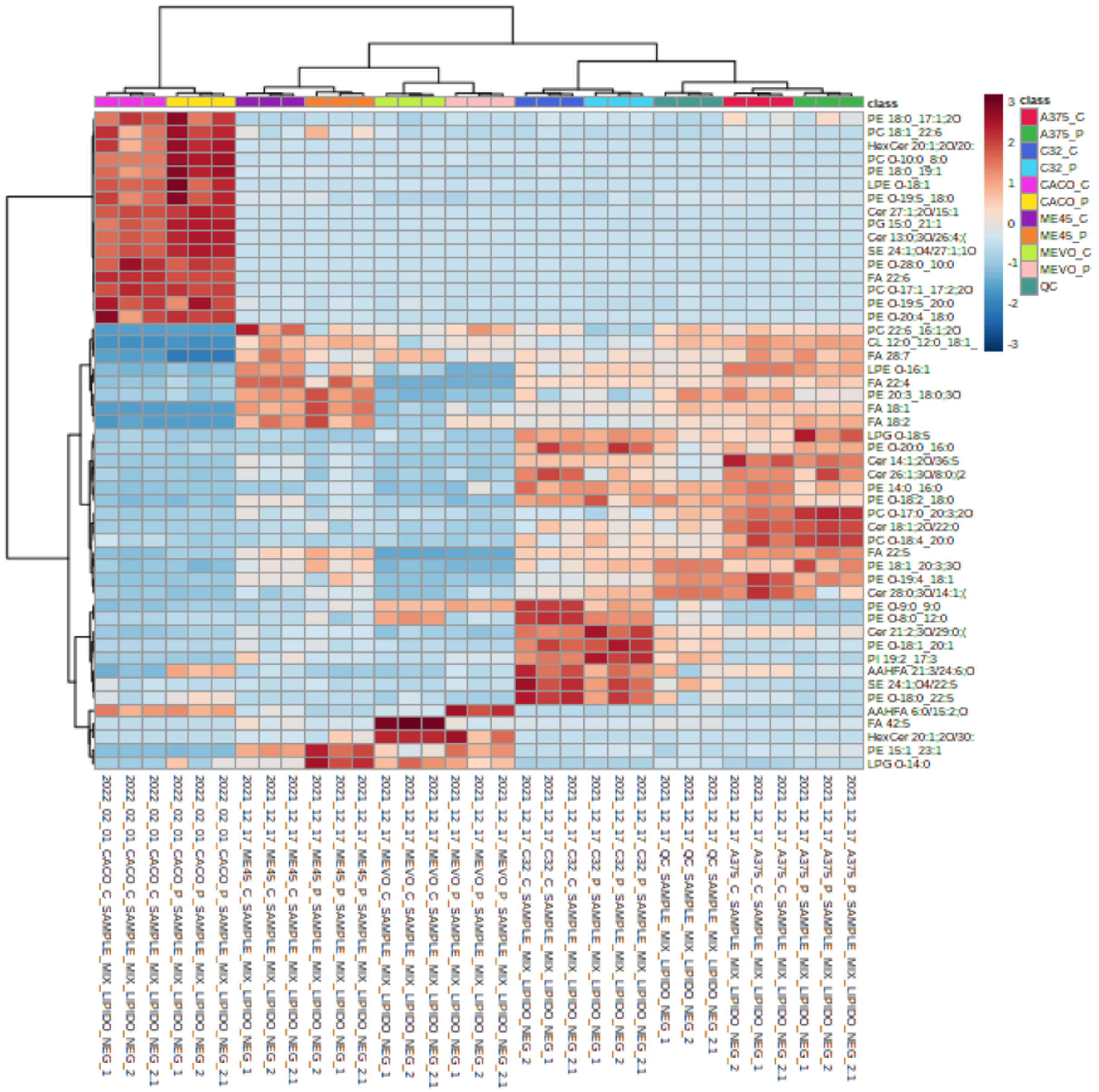
class
PE 18:0_17:1;2O
PC 18:1_22:6
HexCer 20:1;2O/20:
PC O-10:0_8:0
PE 18:0_19:1
LPE O-18:1
PE O-19:5_18:0
Cer 27:1;2O/15:1
PG 15:0_21:1
Cer 13:0;3O/26:4;(
SE 24:1;O4/27:1;1O
PE O-28:0_10:0
FA 22:6
PC O-17:1_17:2;2O
PE O-19:5_20:0
PE O-20:4_18:0
PC 22:6_16:1;2O
CL 12:0_12:0_18:1_
FA 28:7
LPE O-16:1
FA 22:4
PE 20:3_18:0;3O
FA 18:1
FA 18:2
LPG O-18:5
PE O-20:0_16:0
Cer 14:1;2O/36:5
Cer 26:1;3O/8:0;(2
PE 14:0_16:0
PE O-18:2_18:0
PC O-17:0_20:3;2O
Cer 18:1;2O/22:0
PC O-18:4_20:0
FA 22:5
PE 18:1_20:3;3O
PE O-19:4_18:1
Cer 28:0;3O/14:1;(
PE O-9:0_9:0
PE O-8:0_12:0
Cer 21:2;3O/29:0;(
PE O-18:1_20:1
PI 19:2_17:3
AAHFA 21:3/24:6;O
SE 24:1;O4/22:5
PE O-18:0_22:5
AAHFA 6:0/15:2;O
FA 42:5
HexCer 20:1;2O/30:
PE 15:1_23:1
LPG O-14:0
3
2
1
0
-1
-2
-3
class
A375_C
A375_P
C32_C
C32_P
CACO_C
CACO_P
ME45_C
ME45_P
MEVO_C
MEVO_P
QC
2022_02_01_CACO_C_SAMPLE_MIX_LIPIDO_NEG_1
2022_02_01_CACO_C_SAMPLE_MIX_LIPIDO_NEG_2
2022_02_01_CACO_C_SAMPLE_MIX_LIPIDO_NEG_2.1
2022_02_01_CACO_P_SAMPLE_MIX_LIPIDO_NEG_1
2022_02_01_CACO_P_SAMPLE_MIX_LIPIDO_NEG_2
2022_02_01_CACO_P_SAMPLE_MIX_LIPIDO_NEG_2.1
2021_12_17_ME45_C_SAMPLE_MIX_LIPIDO_NEG_1
2021_12_17_ME45_C_SAMPLE_MIX_LIPIDO_NEG_2
2021_12_17_ME45_C_SAMPLE_MIX_LIPIDO_NEG_2.1
2021_12_17_ME45_P_SAMPLE_MIX_LIPIDO_NEG_2
2021_12_17_ME45_P_SAMPLE_MIX_LIPIDO_NEG_1
2021_12_17_ME45_P_SAMPLE_MIX_LIPIDO_NEG_2.1
2021_12_17_MEVO_C_SAMPLE_MIX_LIPIDO_NEG_1
2021_12_17_MEVO_C_SAMPLE_MIX_LIPIDO_NEG_2
2021_12_17_MEVO_C_SAMPLE_MIX_LIPIDO_NEG_2.1
2021_12_17_MEVO_P_SAMPLE_MIX_LIPIDO_NEG_1
2021_12_17_MEVO_P_SAMPLE_MIX_LIPIDO_NEG_2
2021_12_17_MEVO_P_SAMPLE_MIX_LIPIDO_NEG_2.1
2021_12_17_C32_C_SAMPLE_MIX_LIPIDO_NEG_2
2021_12_17_C32_C_SAMPLE_MIX_LIPIDO_NEG_1
2021_12_17_C32_C_SAMPLE_MIX_LIPIDO_NEG_2.1
2021_12_17_C32_P_SAMPLE_MIX_LIPIDO_NEG_1
2021_12_17_C32_P_SAMPLE_MIX_LIPIDO_NEG_2
2021_12_17_C32_P_SAMPLE_MIX_LIPIDO_NEG_2.1
2021_12_17_QC_SAMPLE_MIX_LIPIDO_NEG_1
2021_12_17_QC_SAMPLE_MIX_LIPIDO_NEG_2
2021_12_17_QC_SAMPLE_MIX_LIPIDO_NEG_2.1
2021_12_17_A375_C_SAMPLE_MIX_LIPIDO_NEG_2
2021_12_17_A375_C_SAMPLE_MIX_LIPIDO_NEG_1
2021_12_17_A375_C_SAMPLE_MIX_LIPIDO_NEG_2.1
2021_12_17_A375_P_SAMPLE_MIX_LIPIDO_NEG_1
2021_12_17_A375_P_SAMPLE_MIX_LIPIDO_NEG_2
2021_12_17_A375_P_SAMPLE_MIX_LIPIDO_NEG_2.1

C

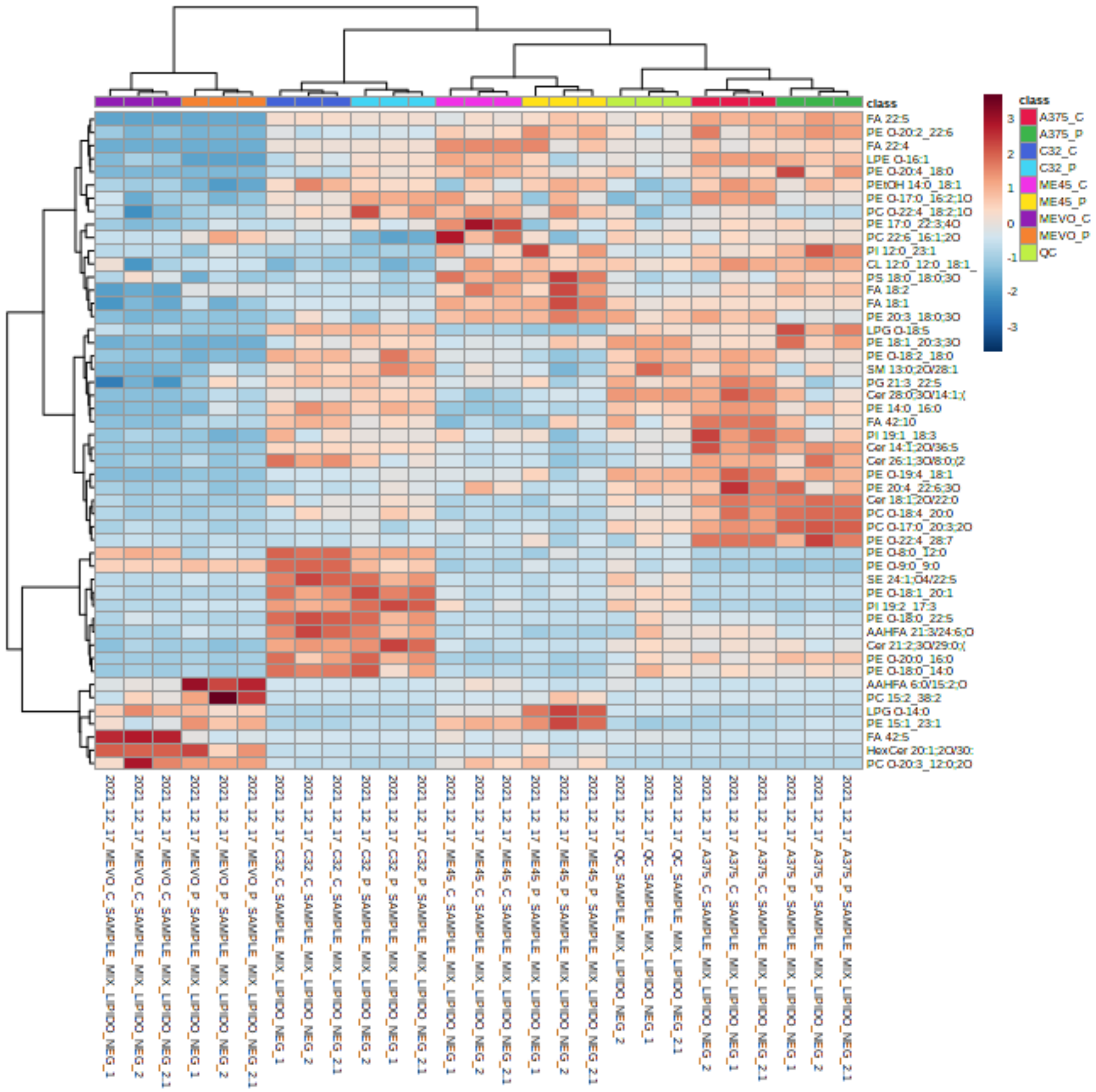

class
FA 22:5
PE O-20:2_22:6
FA 22:4
LPE O-16:1
PE O-20:4_18:0
PEtOH 14:0_18:1
PE O-17:0_16:2;1O
PC O-22:4_18:2;1O
PE 17:0_22:3;4O
PC 22:6_16:1;2O
PI 12:0_23:1
CL 12:0_12:0_18:1_
PS 18:0_18:0;3O
FA 18:2
FA 18:1
PE 20:3_18:0;3O
LPG O-18:5
PE 18:1_20:3;3O
PE O-18:2_18:0
SM 13:0;2O/28:1
PG 21:3_22:5
Cer 28:0;3O/14:1;(
PE 14:0_16:0
FA 42:10
PI 19:1_18:3
Cer 14:1;2O/36:5
Cer 26:1;3O/8:0;(2
PE O-19:4_18:1
PE 20:4_22:6;3O
Cer 18:1;2O/22:0
PC O-18:4_20:0
PC O-17:0_20:3;2O
PE O-22:4_28:7
PE O-8:0_12:0
PE O-9:0_9:0
SE 24:1;O4/22:5
PE O-18:1_20:1
PI 19:2_17:3
PE O-18:0_22:5
AAHFA 21:3/24:6;O
Cer 21:2;3O/29:0;(
PE O-20:0_16:0
PE O-18:0_14:0
AAHFA 6:0/15:2;O
PC 15:2_38:2
LPG O-14:0
PE 15:1_23:1
FA 42:5
HexCer 20:1;2O/30:
PC O-20:3_12:0;2O
3
2
1
0
-1
-2
-3
class
A375_C
A375_P
C32_C
C32_P
ME45_C
ME45_P
MEVO_C
MEVO_P
QC
2021_12_17_MEVO_C_SAMPLE_MIX_LIPIDO_NEG_1
2021_12_17_MEVO_C_SAMPLE_MIX_LIPIDO_NEG_2
2021_12_17_MEVO_C_SAMPLE_MIX_LIPIDO_NEG_2.1
2021_12_17_MEVO_P_SAMPLE_MIX_LIPIDO_NEG_1
2021_12_17_MEVO_P_SAMPLE_MIX_LIPIDO_NEG_2
2021_12_17_MEVO_P_SAMPLE_MIX_LIPIDO_NEG_2.1
2021_12_17_C32_C_SAMPLE_MIX_LIPIDO_NEG_1
2021_12_17_C32_C_SAMPLE_MIX_LIPIDO_NEG_2
2021_12_17_C32_C_SAMPLE_MIX_LIPIDO_NEG_2.1
2021_12_17_C32_P_SAMPLE_MIX_LIPIDO_NEG_2
2021_12_17_C32_P_SAMPLE_MIX_LIPIDO_NEG_1
2021_12_17_C32_P_SAMPLE_MIX_LIPIDO_NEG_2.1
2021_12_17_ME45_C_SAMPLE_MIX_LIPIDO_NEG_1
2021_12_17_ME45_C_SAMPLE_MIX_LIPIDO_NEG_2
2021_12_17_ME45_C_SAMPLE_MIX_LIPIDO_NEG_2.1
2021_12_17_ME45_P_SAMPLE_MIX_LIPIDO_NEG_1
2021_12_17_ME45_P_SAMPLE_MIX_LIPIDO_NEG_2
2021_12_17_ME45_P_SAMPLE_MIX_LIPIDO_NEG_2.1
2021_12_17_QC_SAMPLE_MIX_LIPIDO_NEG_2
2021_12_17_QC_SAMPLE_MIX_LIPIDO_NEG_1
2021_12_17_QC_SAMPLE_MIX_LIPIDO_NEG_2.1
2021_12_17_A375_C_SAMPLE_MIX_LIPIDO_NEG_2
2021_12_17_A375_C_SAMPLE_MIX_LIPIDO_NEG_1
2021_12_17_A375_C_SAMPLE_MIX_LIPIDO_NEG_2.1
2021_12_17_A375_P_SAMPLE_MIX_LIPIDO_NEG_1
2021_12_17_A375_P_SAMPLE_MIX_LIPIDO_NEG_2
2021_12_17_A375_P_SAMPLE_MIX_LIPIDO_NEG_2.1

D

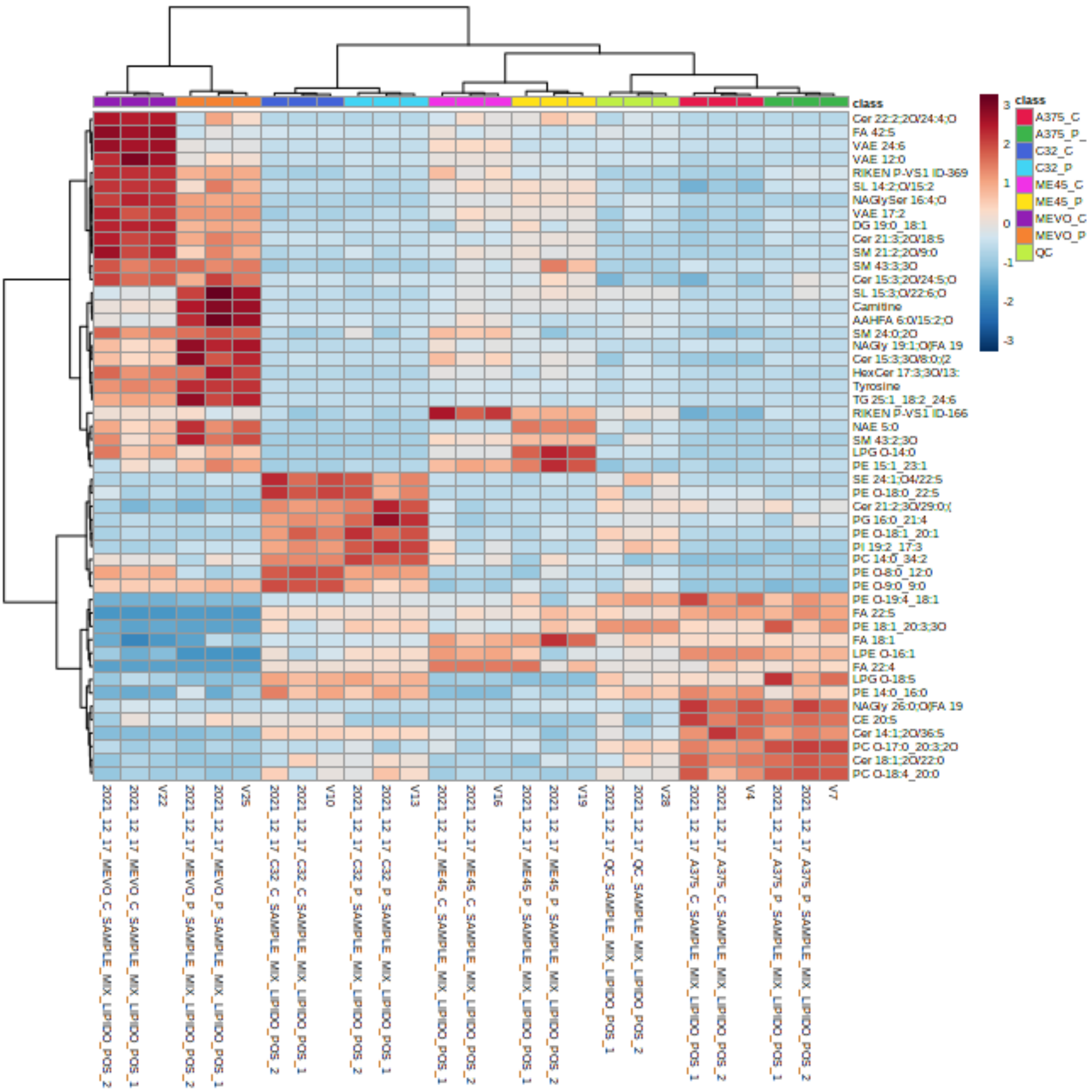
class
Cer 22:2;2O/24:4;O
FA 42:5
VAE 24:6
VAE 12:0
RIKEN P-VS1 ID-369
SL 14:2;O/15:2
NAGlySer 16:4;O
VAE 17:2
DG 19:0_18:1
Cer 21:3;2O/18:5
SM 21:2;2O/9:0
SM 43:3;3O
Cer 15:3;2O/24:5;O
SL 15:3;O/22:6;O
Carnitine
AAHFA 6:0/15:2;O
SM 24:0;2O
NAGly 19:1;O(FA 19
Cer 15:3;3O/8:0;(2
HexCer 17:3;3O/13:
Tyrosine
TG 25:1_18:2_24:6
RIKEN P-VS1 ID-166
NAE 5:0
SM 43:2;3O
LPG O-14:0
PE 15:1_23:1
SE 24:1;O4/22:5
PE O-18:0_22:5
Cer 21:2;3O/29:0;(
PG 16:0_21:4
PE O-18:1_20:1
PI 19:2_17:3
PC 14:0_34:2
PE O-8:0_12:0
PE O-9:0_9:0
PE O-19:4_18:1
FA 22:5
PE 18:1_20:3;3O
FA 18:1
LPE O-16:1
FA 22:4
LPG O-18:5
PE 14:0_16:0
NAGly 26:0;O(FA 19
CE 20:5
Cer 14:1;2O/36:5
PC O-17:0_20:3;2O
Cer 18:1;2O/22:0
PC O-18:4_20:0
3
2
1
0
-1
-2
-3
class
A375_C
A375_P_
C32_C
C32_P
ME45_C
ME45_P
MEVO_C
MEVO_P
QC
2021_12_17_MEVO_C_SAMPLE_MIX_LIPIDO_POS_2
2021_12_17_MEVO_C_SAMPLE_MIX_LIPIDO_POS_1
V22
2021_12_17_MEVO_P_SAMPLE_MIX_LIPIDO_POS_2
2021_12_17_MEVO_P_SAMPLE_MIX_LIPIDO_POS_1
V25
2021_12_17_C32_C_SAMPLE_MIX_LIPIDO_POS_2
2021_12_17_C32_C_SAMPLE_MIX_LIPIDO_POS_1
V10
2021_12_17_C32_P_SAMPLE_MIX_LIPIDO_POS_2
2021_12_17_C32_P_SAMPLE_MIX_LIPIDO_POS_1
V13
2021_12_17_ME45_C_SAMPLE_MIX_LIPIDO_POS_1
2021_12_17_ME45_C_SAMPLE_MIX_LIPIDO_POS_2
V16
2021_12_17_ME45_P_SAMPLE_MIX_LIPIDO_POS_1
2021_12_17_ME45_P_SAMPLE_MIX_LIPIDO_POS_2
V19
2021_12_17_QC_SAMPLE_MIX_LIPIDO_POS_1
2021_12_17_QC_SAMPLE_MIX_LIPIDO_POS_2
V28
2021_12_17_A375_C_SAMPLE_MIX_LIPIDO_POS_1
2021_12_17_A375_C_SAMPLE_MIX_LIPIDO_POS_2
V4
2021_12_17_A375_P_SAMPLE_MIX_LIPIDO_POS_1
2021_12_17_A375_P_SAMPLE_MIX_LIPIDO_POS_2
V7

E

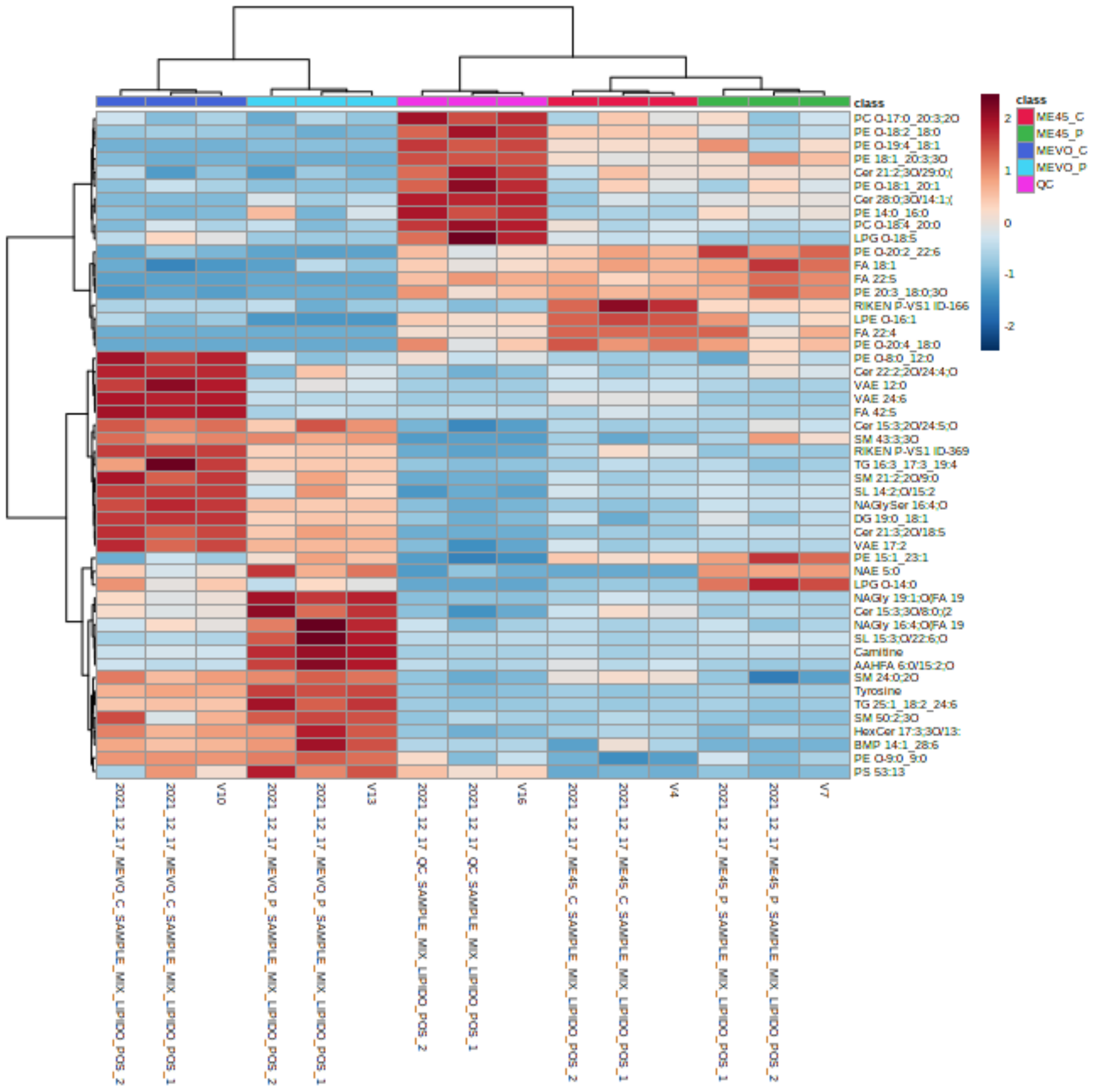

F

Fig. 6. Clustering result shown as heatmap of lipids with positive charge(A) A375, C32, ME45, MeWo and CaCo-2 cell lines (B) A375, C32, ME45, MeWo cell lines, and of lipids with negative charge (C) A375, C32, ME45, MeWo and CaCo-2 cell lines (D) A375, C32, ME45, MeWo cell lines, (E) joined melanoma group (lipids with positive and negative charge), (F) ME45 and MeWo joined group. Distance measure using euclidean, and clustering algorithm using ward.D)

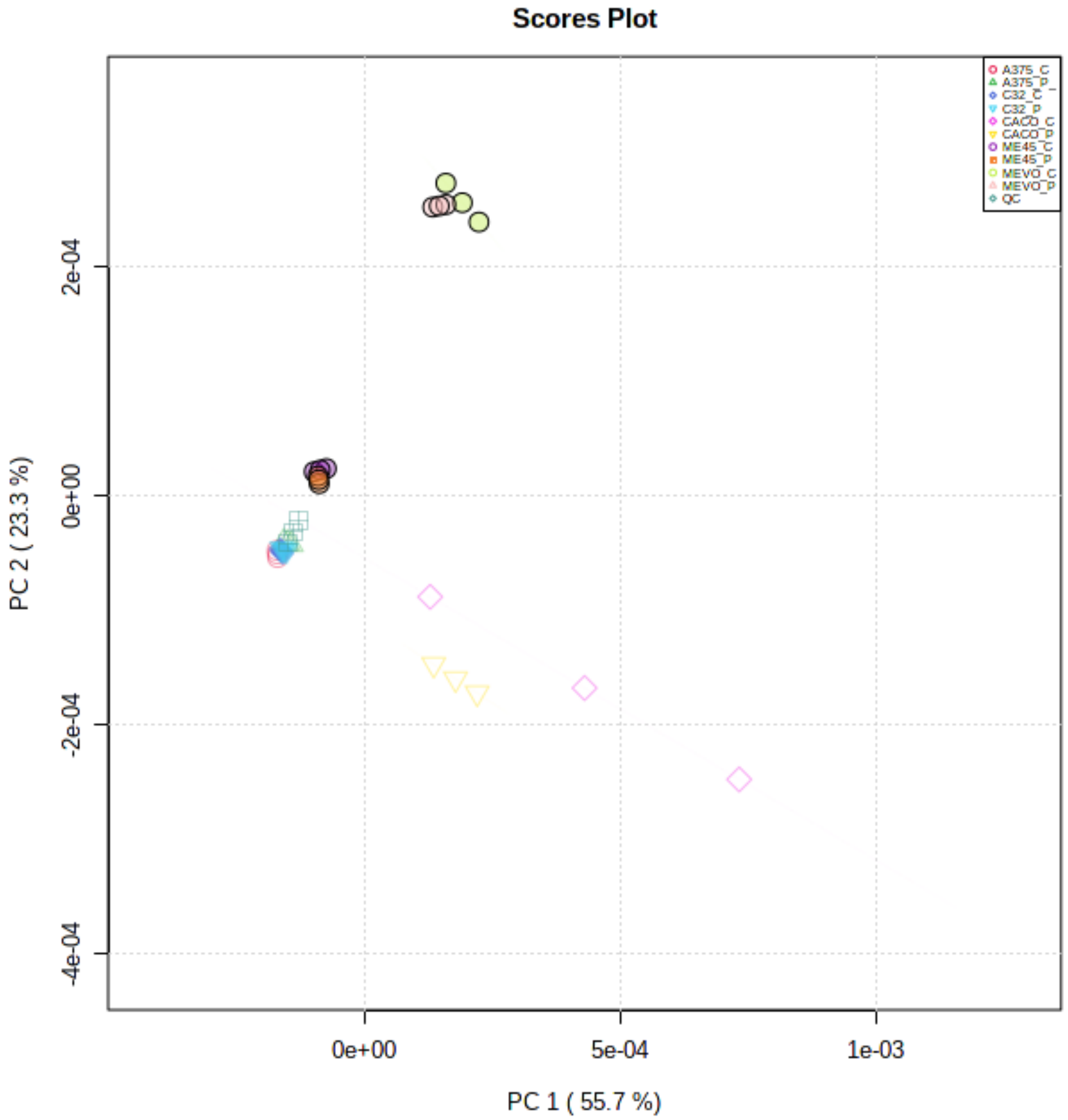
Scores Plot
A375_C
A375_P
C32_C
C32_P
CACO_C
CACO_P
ME45_C
ME45_P
MEVO_C
MEVO_P
QC
2e-04
0e+00
-2e-04
-4e-04
PC 2 (23.3 %)
0e+00
5e-04
1e-03
PC 1 (55.7 %)

A

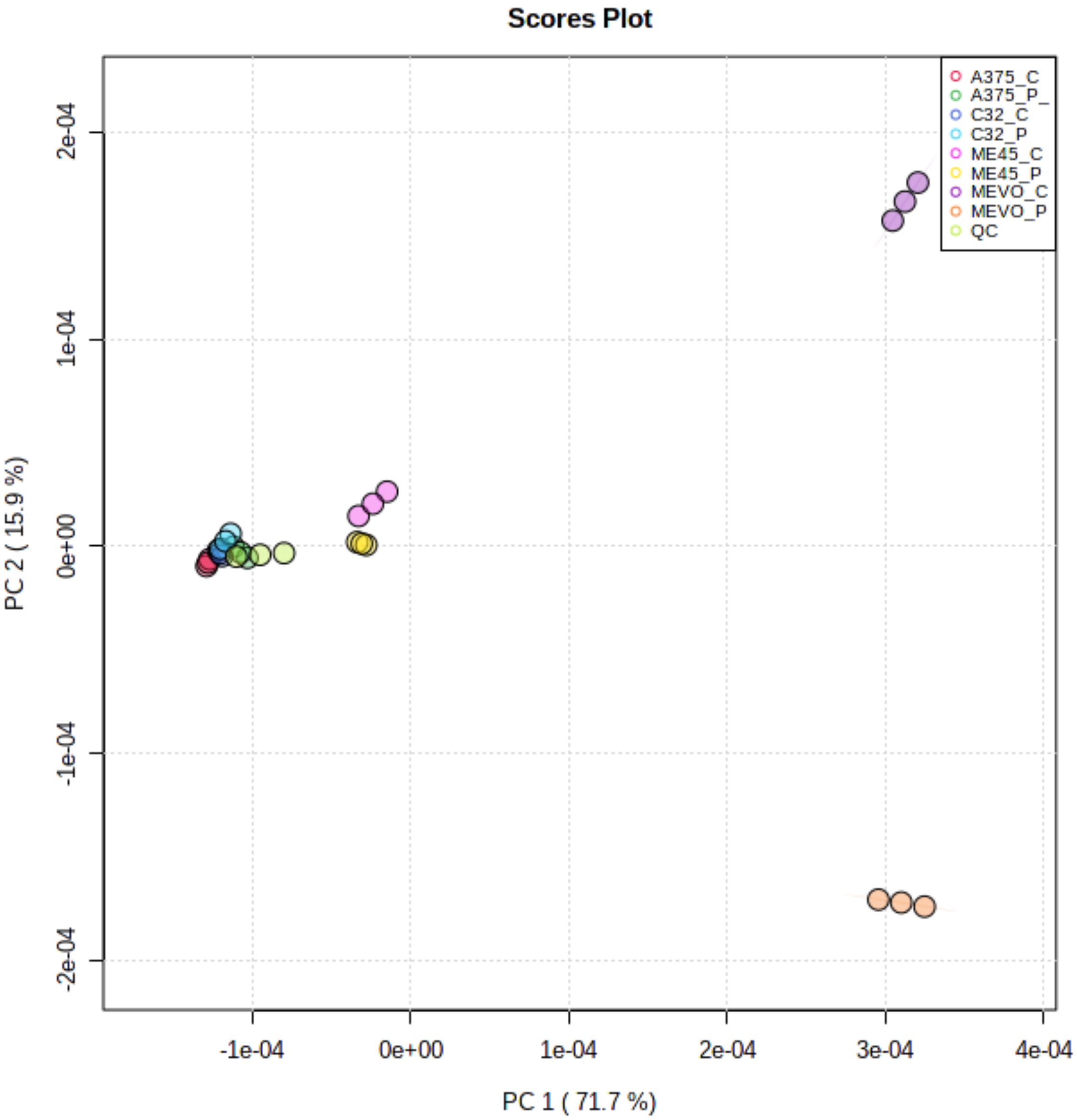

Scores Plot
A375_C
A375_P_
C32_C
C32_P
ME45_C
ME45_P
MEVO_C
MEVO_P
QC
2e-04
1e-04
0e+00
-1e-04
-2e-04
PC 2 (15.9 %)
-1e-04
0e+00
1e-04
2e-04
3e-04
4e-04
PC 1 (71.7 %)

B

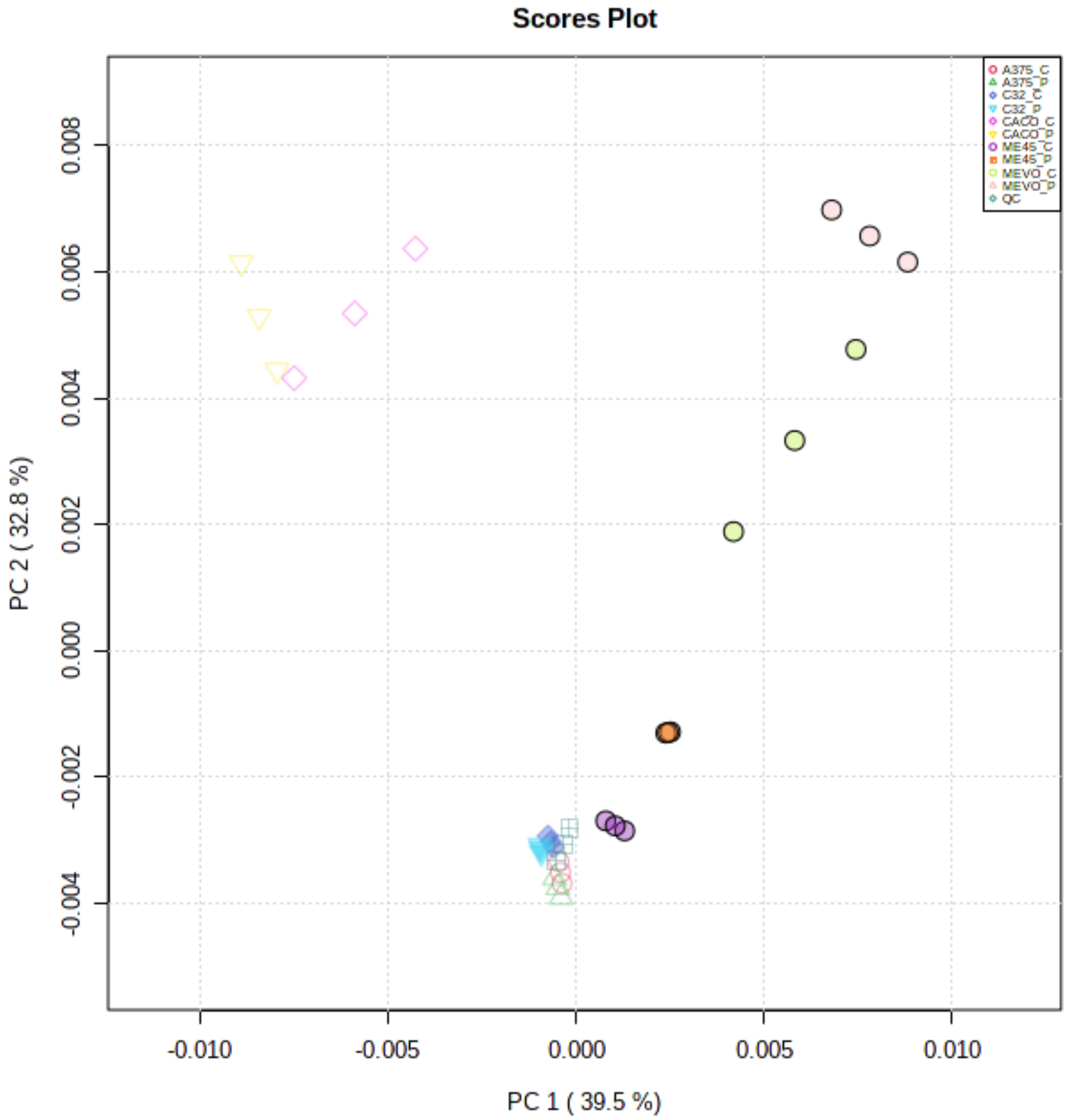
Scores Plot
A375_C
A375_P
C32_C
C32_P
CACO_C
CACO_P
ME45_C
ME45_P
MEVO_C
MEVO_P
QC
PC 2 (32.8 %)
0.008
0.006
0.004
0.002
0.000
-0.002
-0.004
-0.010
-0.005
0.000
0.005
0.010
PC 1 (39.5 %)

C

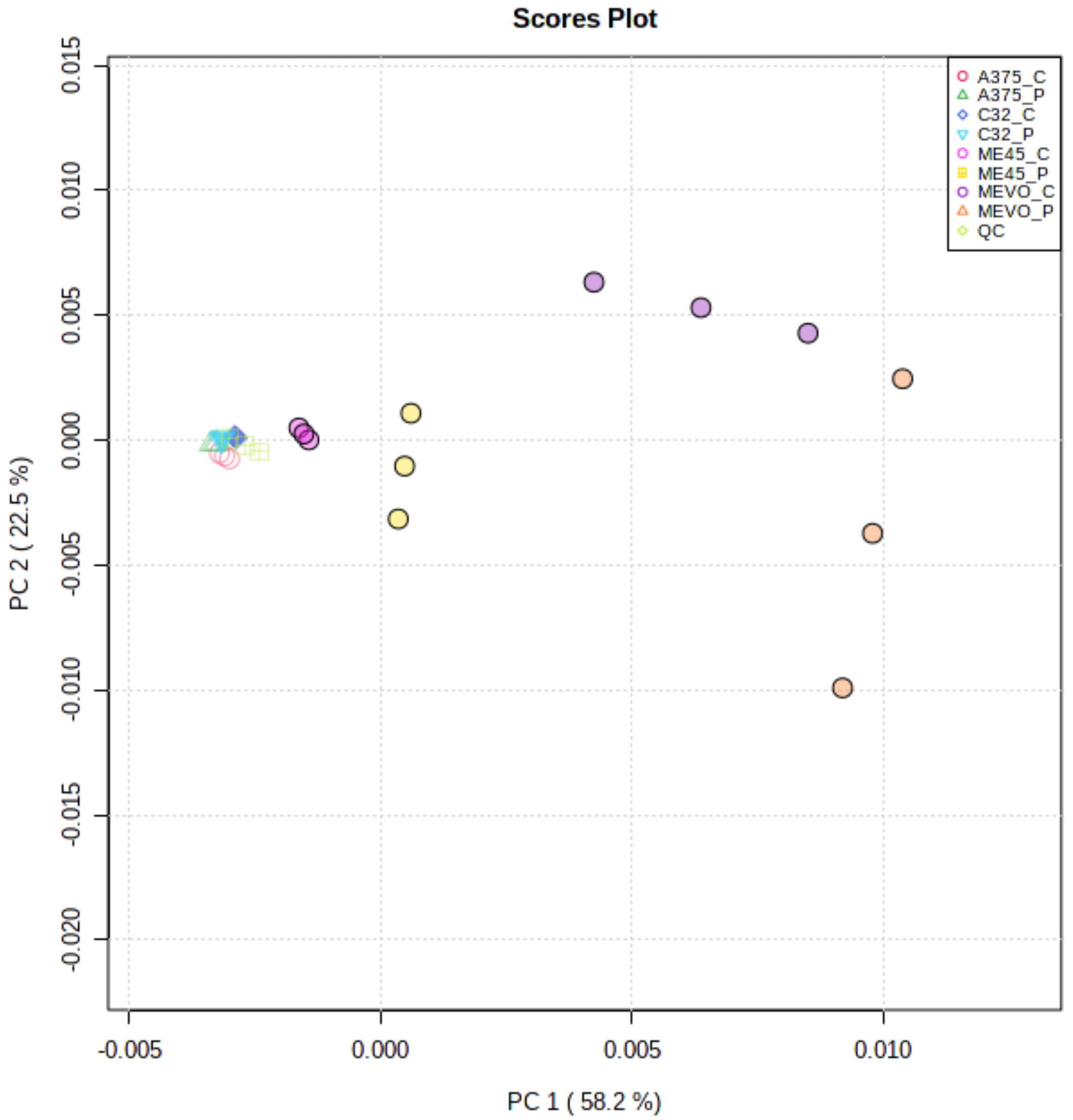
Scores Plot
A375_C
A375_P
C32_C
C32_P
ME45_C
ME45_P
MEVO_C
MEVO_P
QC
PC 1 (58.2 %)
PC 2 (22.5 %)
-0.005
0.000
0.005
0.010
0.015
-0.010
-0.015
-0.020

D

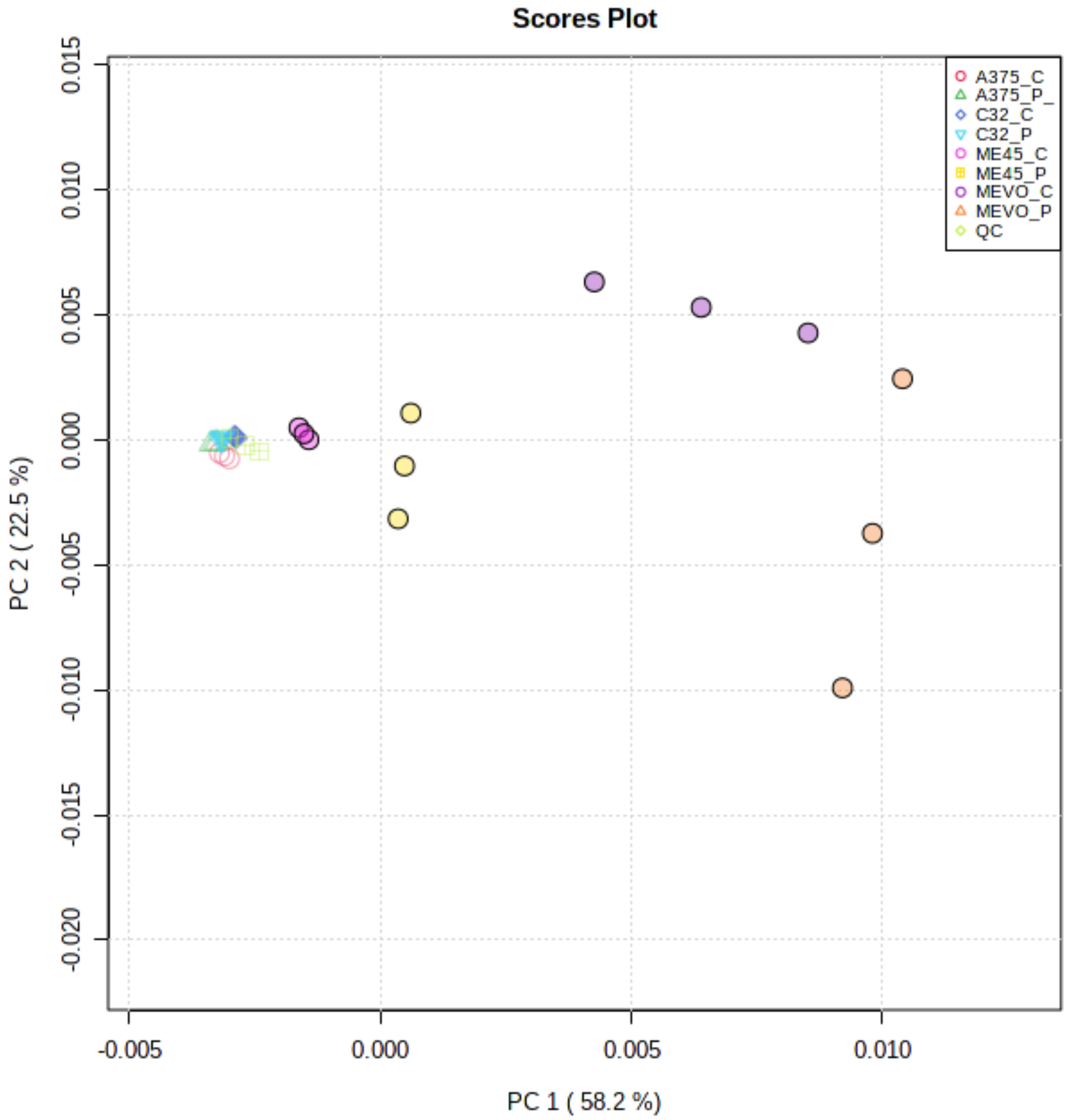

Scores Plot
A375_C
A375_P_
C32_C
C32_P
ME45_C
ME45_P
MEVO_C
MEVO_P
QC
PC 2 (22.5 %)
PC 1 (58.2 %)
0.015
0.010
0.005
0.000
-0.005
-0.010
-0.015
-0.020
-0.005
0.000
0.005
0.010

E

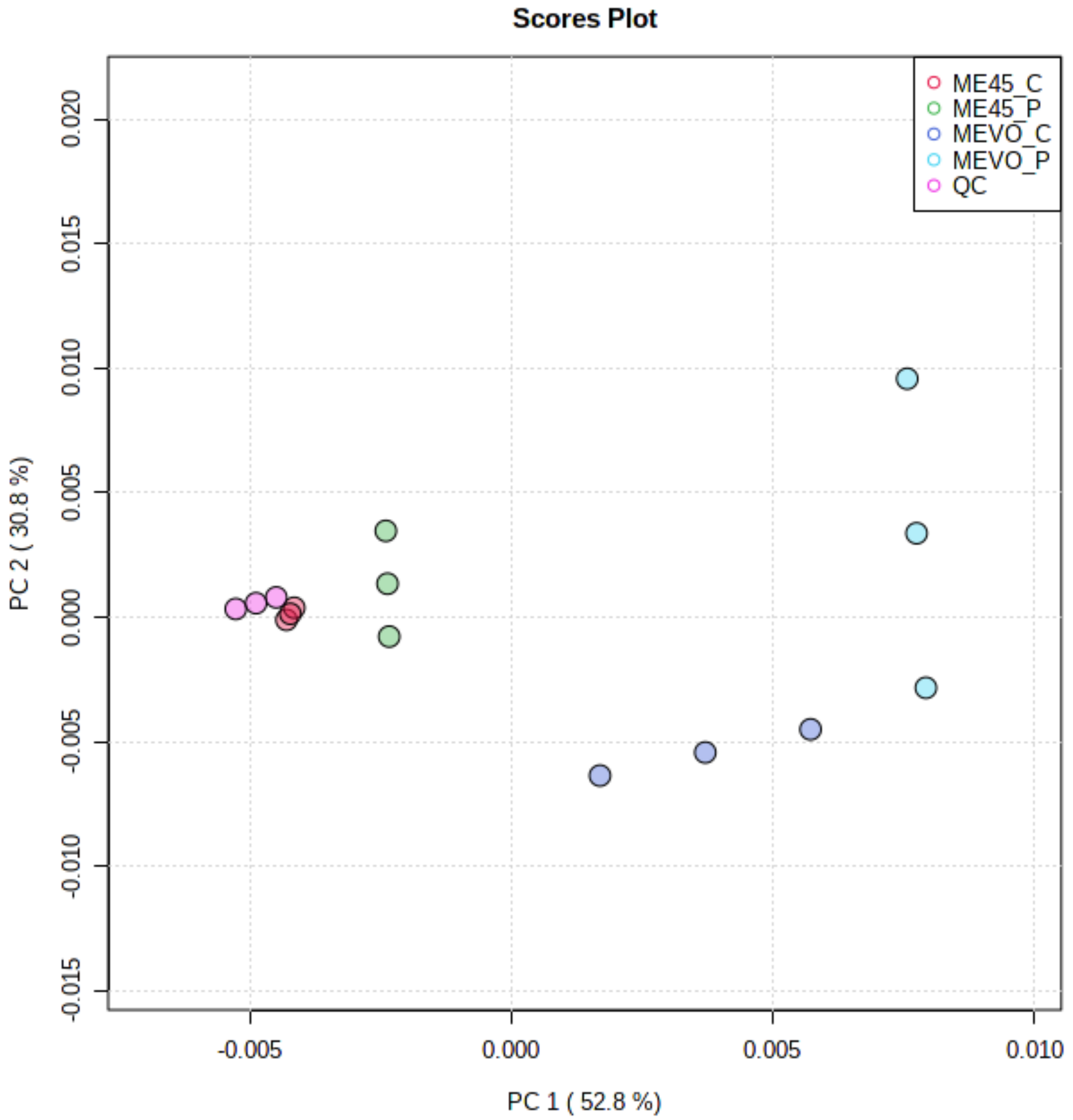

F

Fig. 7. Principal component analysis (PCA) scores of lipids with positive charge (A) A375, C32, ME45, MeWo and CaCo-2 cell lines (B)) A375, C32, ME45, MeWo cell lines, and of lipids with negative charge (C) A375, C32, ME45, MeWo and CaCo-2 cell lines (D) A375, C32, ME45, MeWo cell lines,(E) joined group (lipids with positive and negative charge), (F) ME45 and MeWo joined group. Scores plot between the selected PCs. The explained variances are shown in brackets.

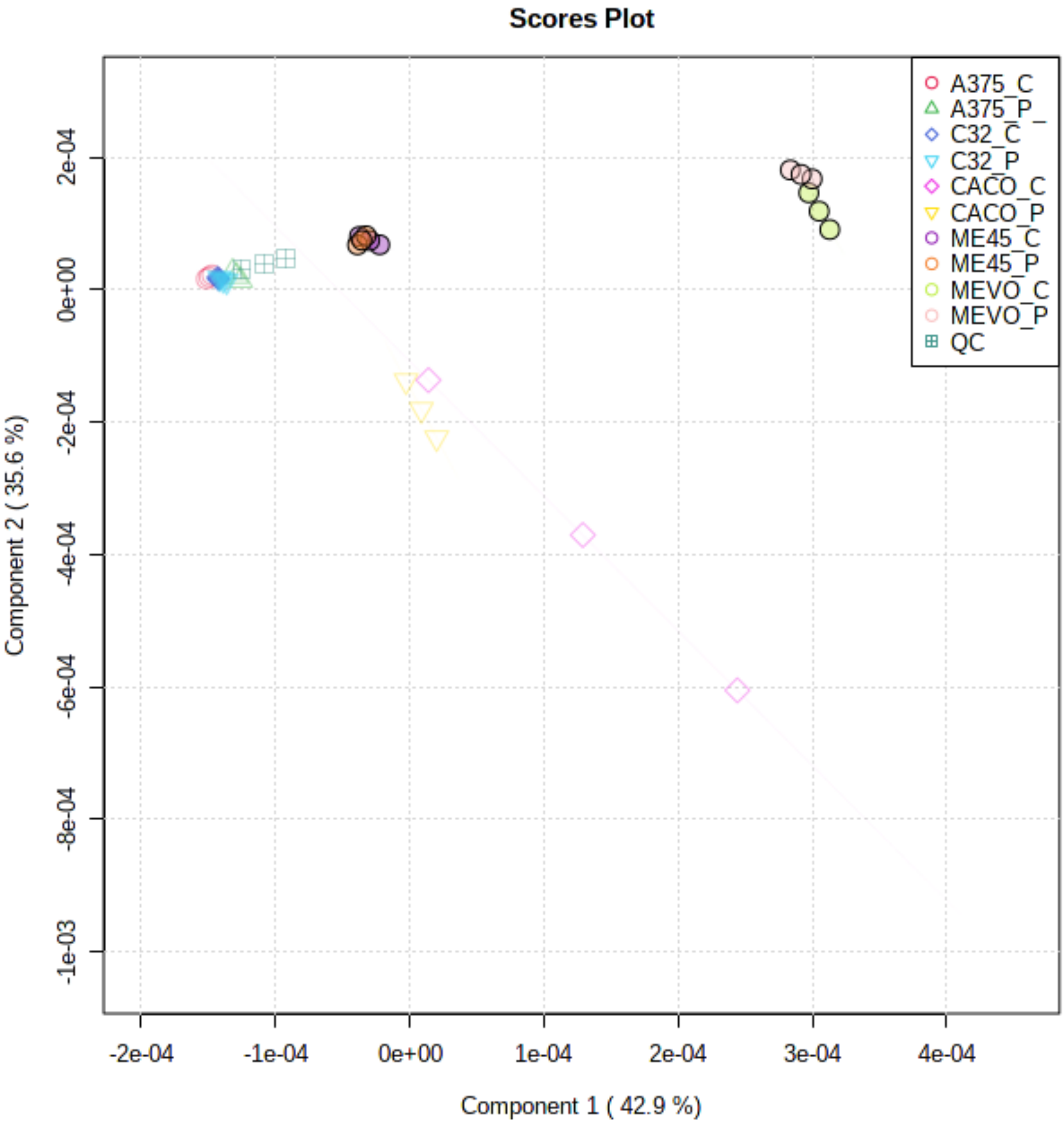
Scores Plot
A375_C
A375_P_
C32_C
C32_P
CACO_C
CACO_P
ME45_C
ME45_P
MEVO_C
MEVO_P
QC
2e-04
0e+00
-2e-04
-4e-04
-6e-04
-8e-04
-1e-03
Component 2 (35.6 %)
-2e-04
-1e-04
0e+00
1e-04
2e-04
3e-04
4e-04
Component 1 (42.9 %)

A

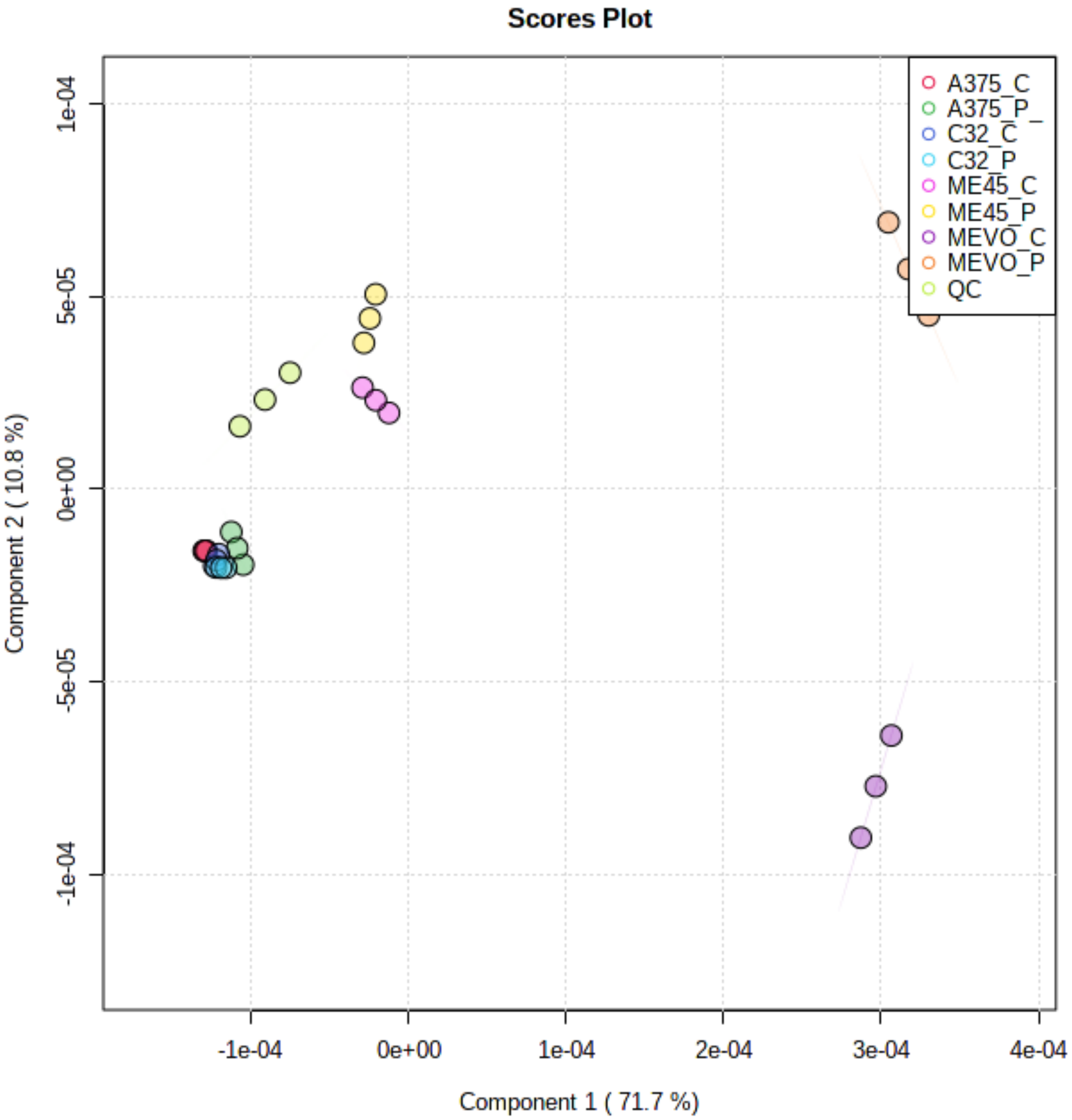
Scores Plot
A375_C
A375_P_
C32_C
C32_P
ME45_C
ME45_P
MEVO_C
MEVO_P
QC
Component 2 (10.8 %)
1e-04
5e-05
0e+00
-5e-05
-1e-04
-1e-04
0e+00
1e-04
2e-04
3e-04
4e-04
Component 1 (71.7 %)

B

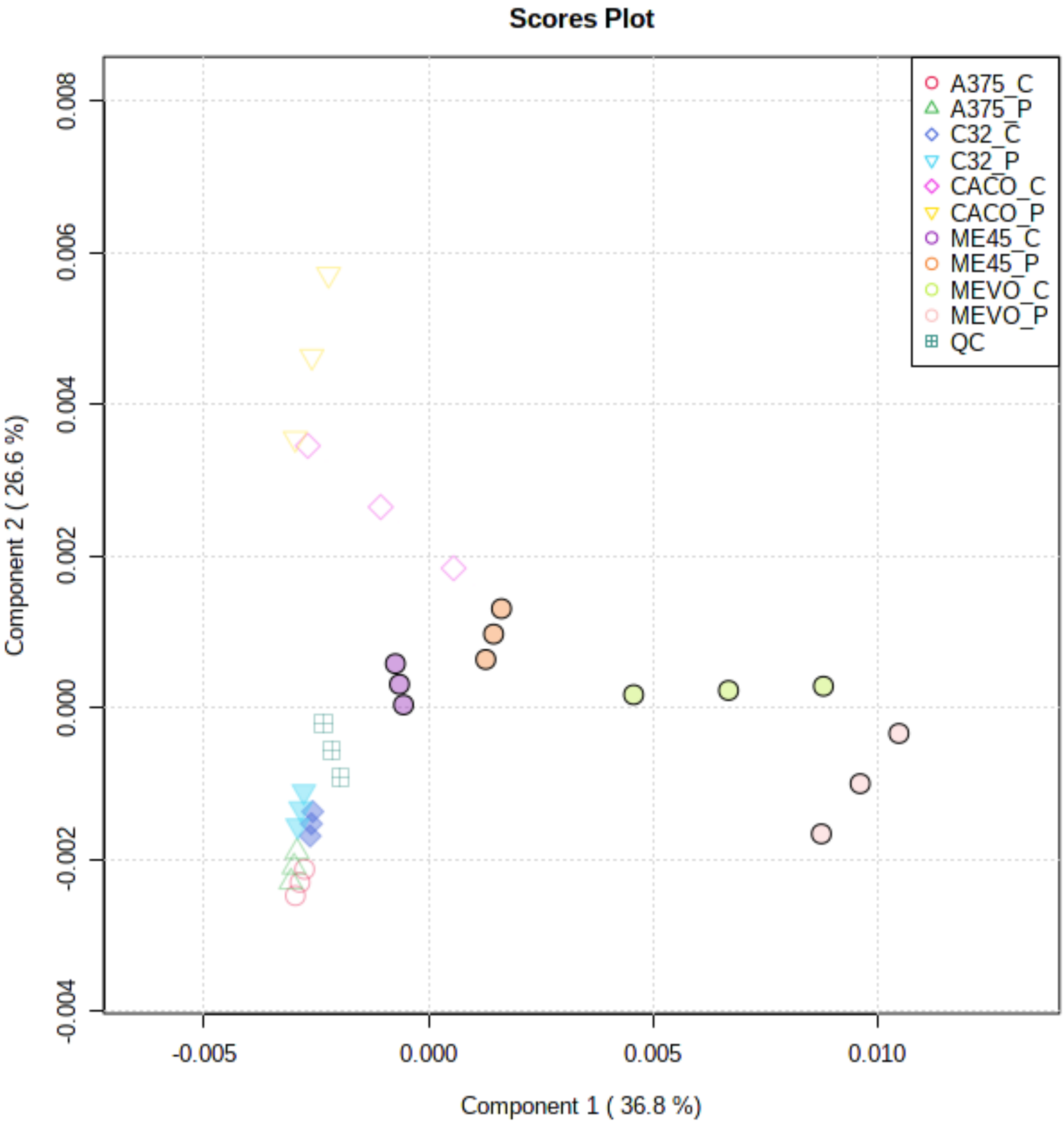

Scores Plot
A375_C
A375_P
C32_C
C32_P
CACO_C
CACO_P
ME45_C
ME45_P
MEVO_C
MEVO_P
QC
Component 2 (26.6 %)
0.008
0.006
0.004
0.002
0.000
-0.002
-0.004
-0.005
0.000
0.005
0.010
Component 1 (36.8 %)

C

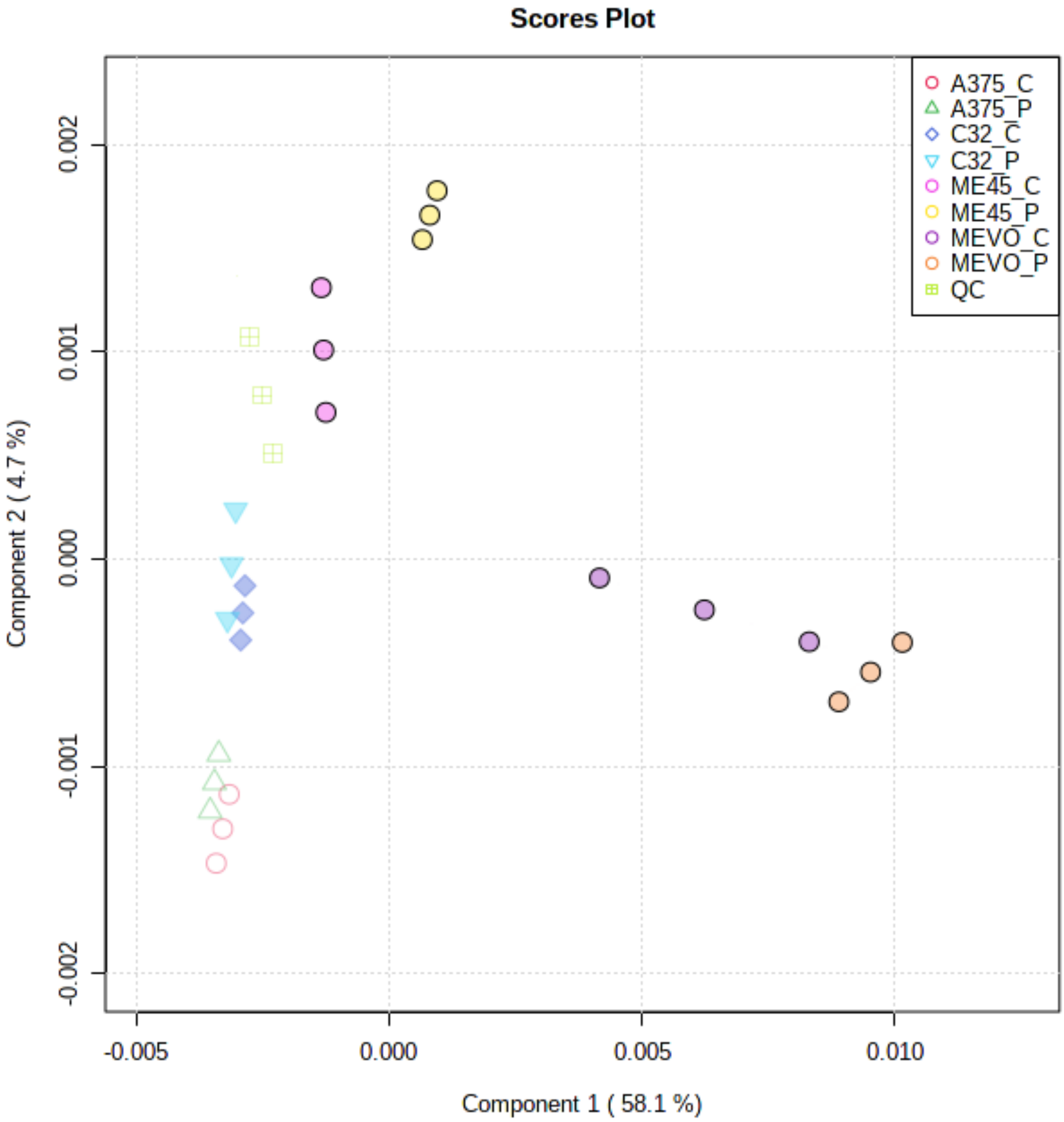

Scores Plot
A375_C
A375_P
C32_C
C32_P
ME45_C
ME45_P
MEVO_C
MEVO_P
QC
0.002
0.001
0.000
-0.001
-0.002
Component 2 (4.7 %)
-0.005
0.000
0.005
0.010
Component 1 (58.1 %)

D

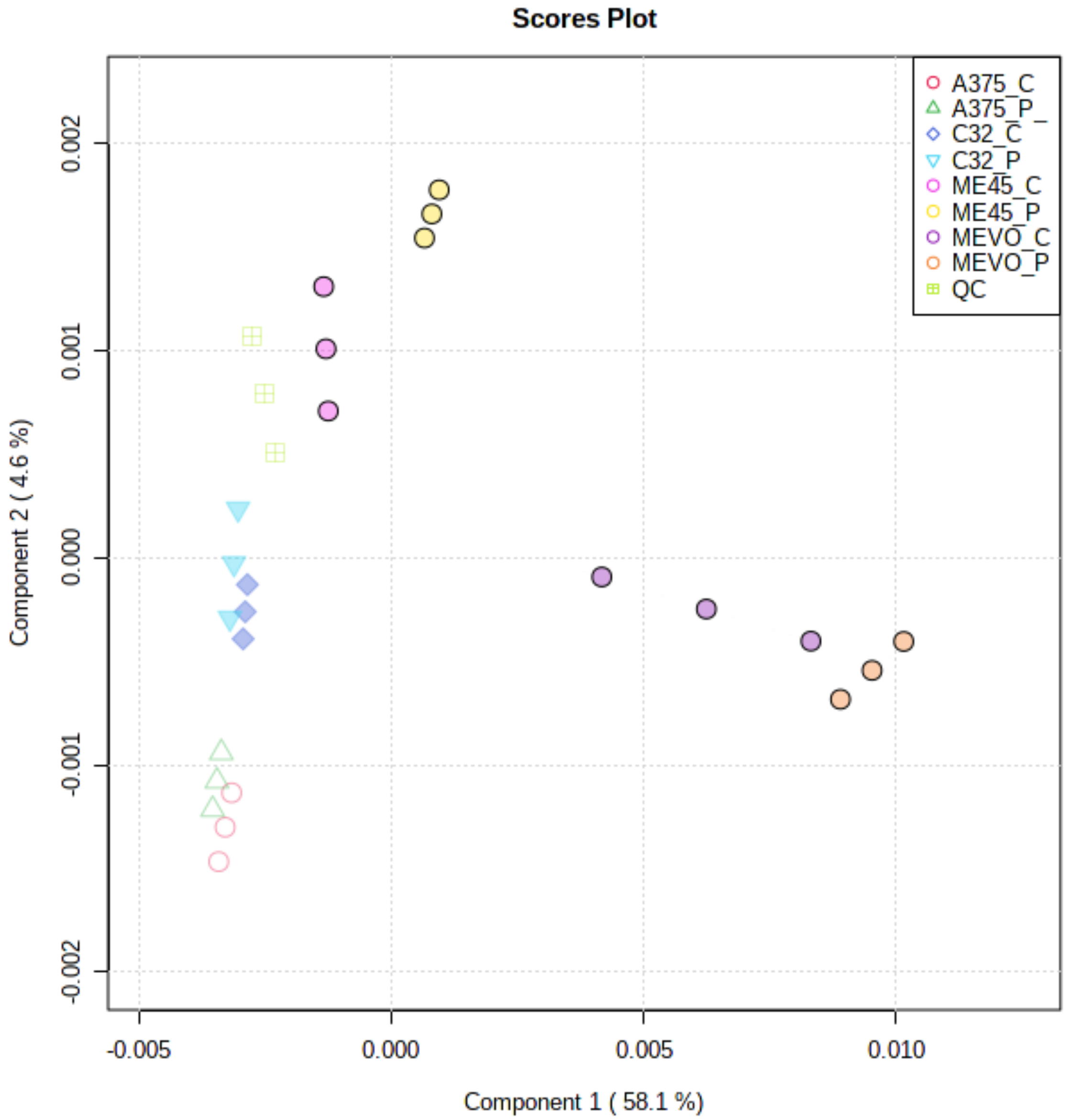

Scores Plot
A375_C
A375_P_
C32_C
C32_P
ME45_C
ME45_P
MEVO_C
MEVO_P
QC
Component 2 (4.6 %)
0.002
0.001
0.000
-0.001
-0.002
-0.005
0.000
0.005
0.010
Component 1 (58.1 %)

E

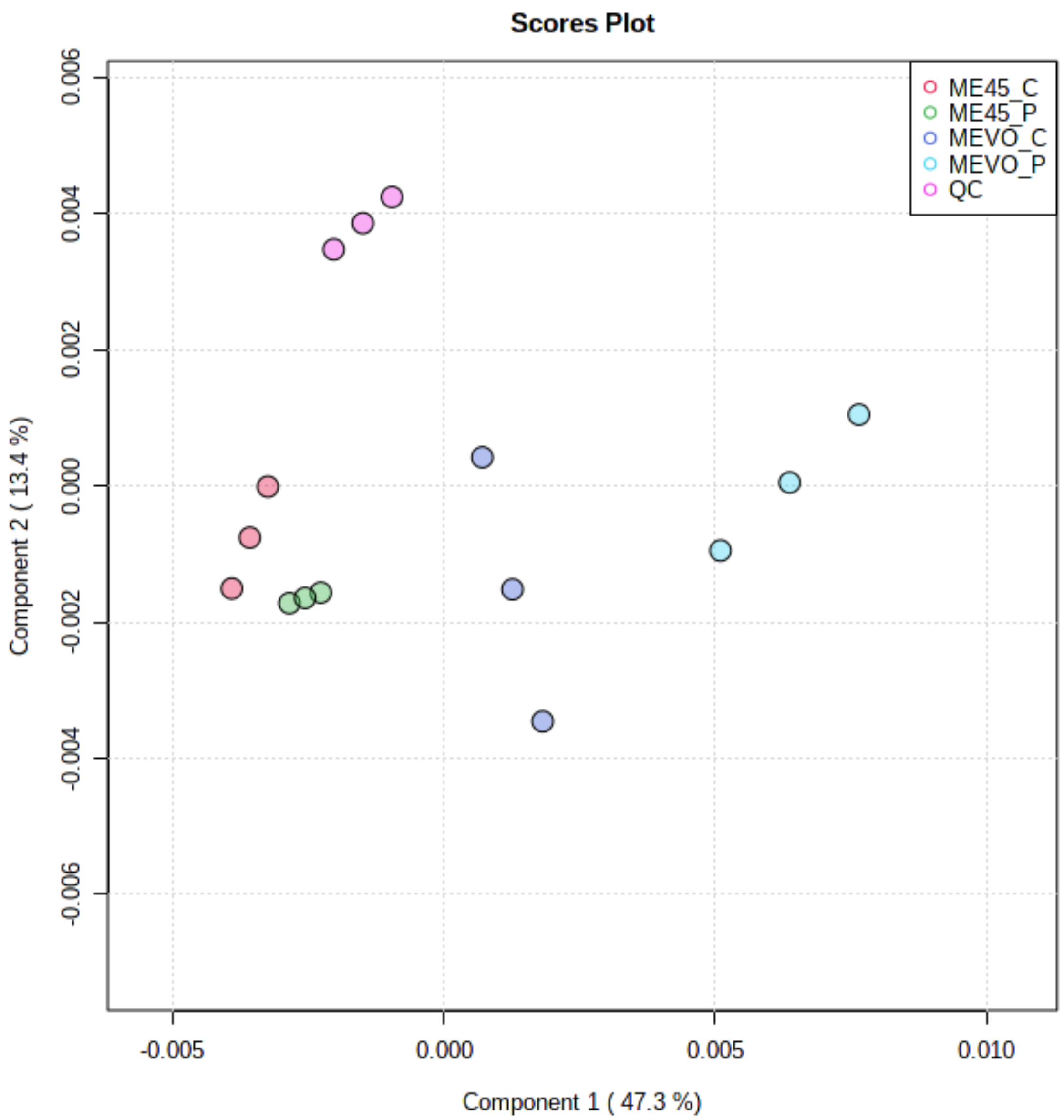

F

Fig. 8. Partial least squares-discriminant analysis (PLS-DA) scores of lipids with positive charge (A) A375, C32, ME45, MeWo and CaCo-2 cell lines (B)) A375, C32, ME45, MeWo cell lines, and of lipids with negative charge (C) A375, C32, ME45, MeWo and CaCo-2 cell lines (D) A375, C32, ME45, MeWo cell lines, (E) joined group (lipids with positive and negative charge), (F) ME45 and MeWo joined group. Scores plot between the selected PCs. The explained variances are shown in brackets.

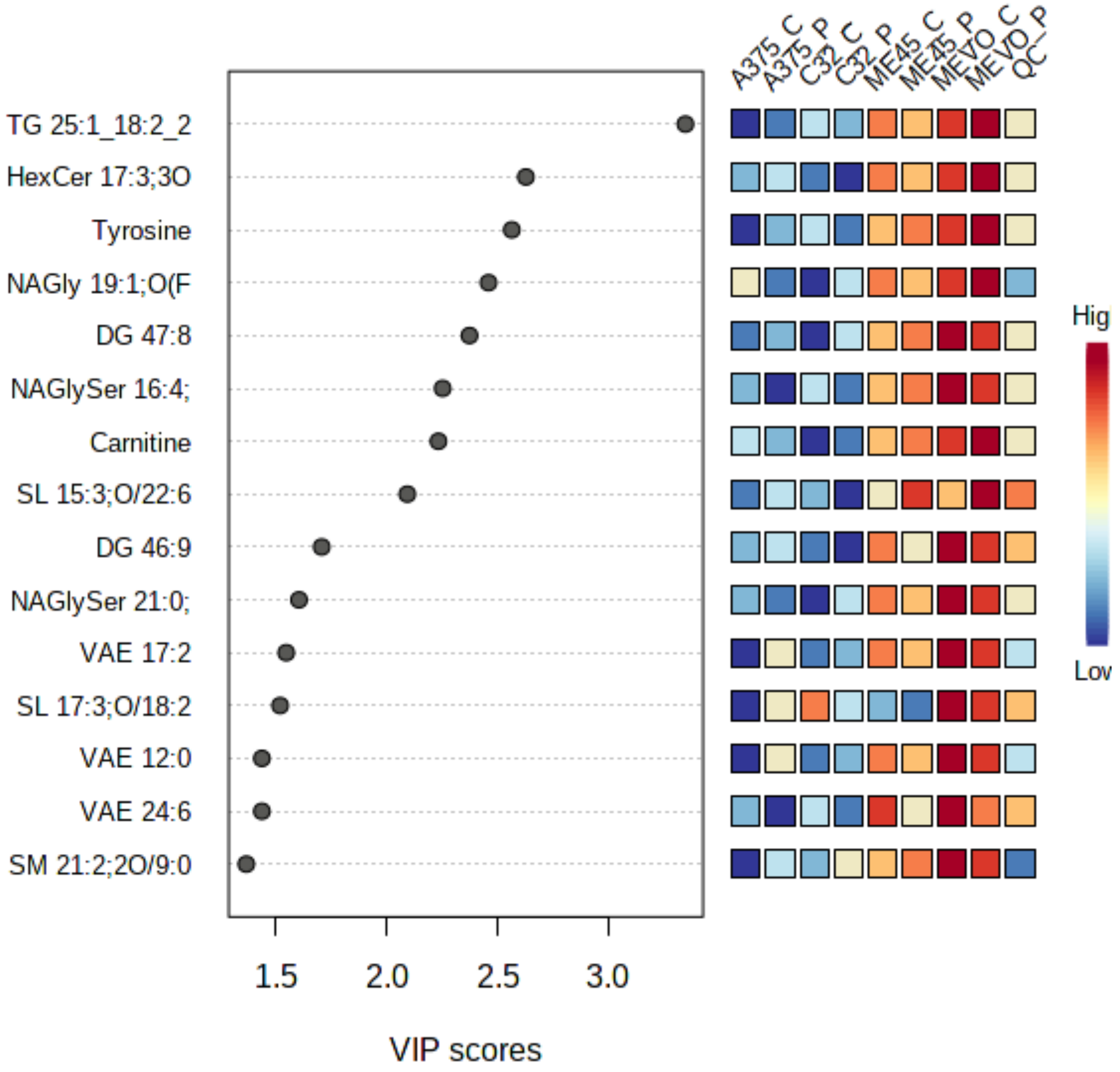

A375_C
A375_P
C32_C
C32_P
ME45_C
ME45_P
MEVO_C
MEVO_P
QC
TG 25:1_18:2_2
HexCer 17:3;3O
Tyrosine
NAGly 19:1;O(F
DG 47:8
NAGlySer 16:4;
Carnitine
SL 15:3;O/22:6
DG 46:9
NAGlySer 21:0;
VAE 17:2
SL 17:3;O/18:2
VAE 12:0
VAE 24:6
SM 21:2;2O/9:0
Hig
Low
1.5
2.0
2.5
3.0
VIP scores

A

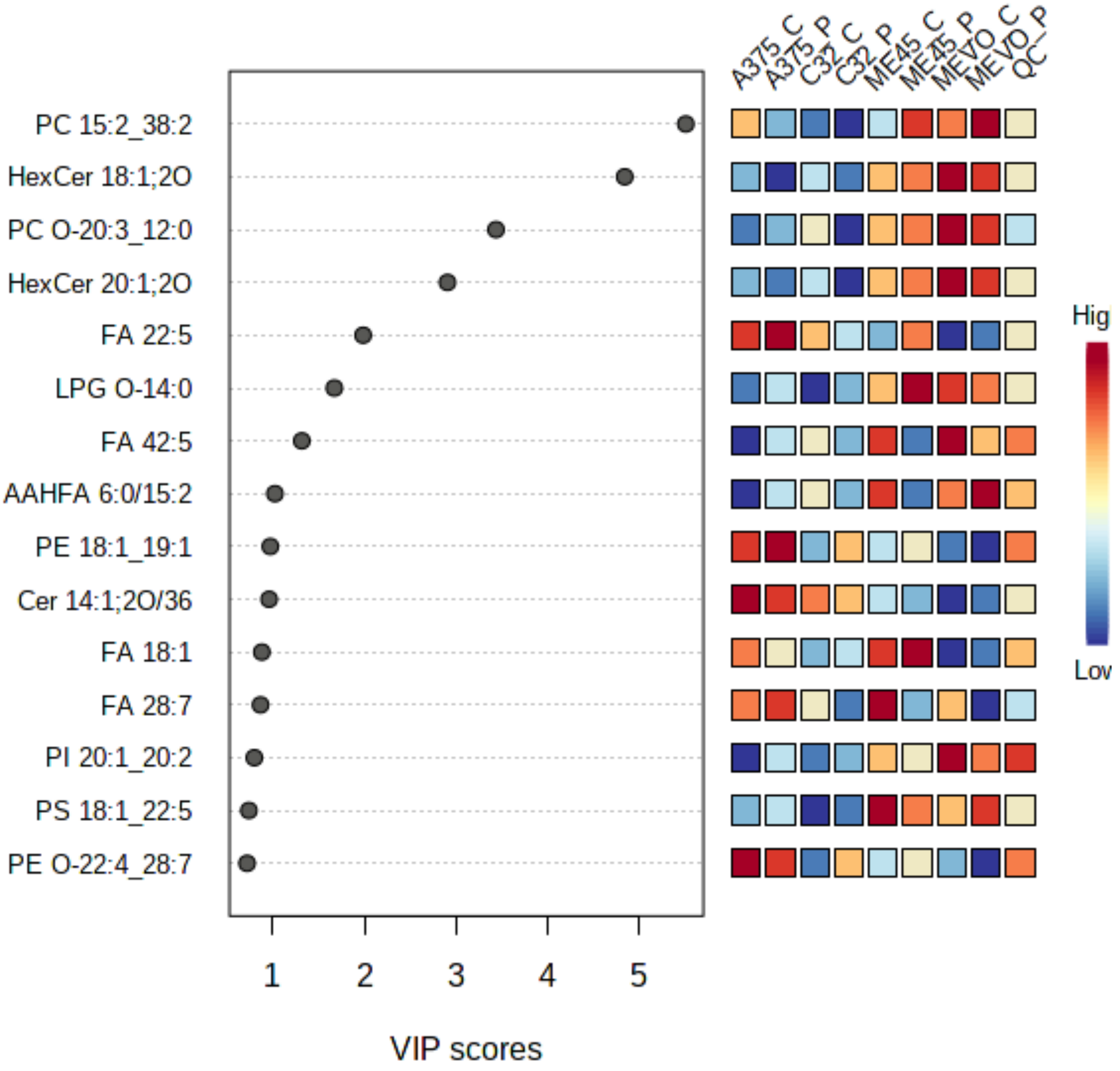
A375_C
A375_P
C32_C
C32_P
ME45_C
ME45_P
MEVO_C
MEVO_P
QC
PC 15:2_38:2
HexCer 18:1;2O
PC O-20:3_12:0
HexCer 20:1;2O
FA 22:5
LPG O-14:0
FA 42:5
AAHFA 6:0/15:2
PE 18:1_19:1
Cer 14:1;2O/36
FA 18:1
FA 28:7
PI 20:1_20:2
PS 18:1_22:5
PE O-22:4_28:7
1
2
3
4
5
VIP scores
Hig
Low

B

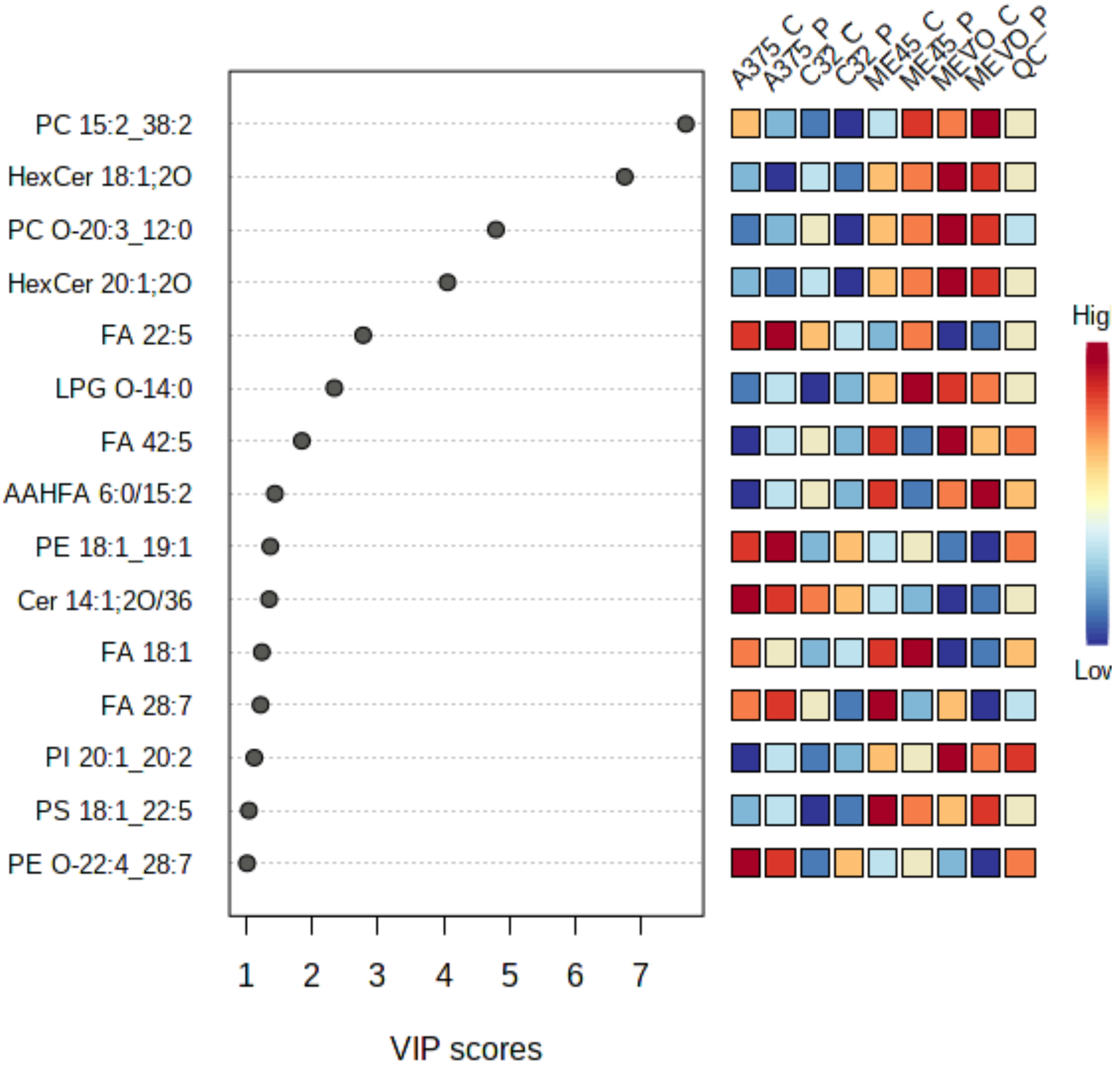

PC 15:2_38:2
HexCer 18:1;2O
PC O-20:3_12:0
HexCer 20:1;2O
FA 22:5
LPG O-14:0
FA 42:5
AAHFA 6:0/15:2
PE 18:1_19:1
Cer 14:1;2O/36
FA 18:1
FA 28:7
PI 20:1_20:2
PS 18:1_22:5
PE O-22:4_28:7
1
2
3
4
5
6
7
VIP scores
A375_C
A375_P
C32_C
C32_P
ME45_C
ME45_P
MEVO_C
MEVO_P
QC
Hig
Low

C

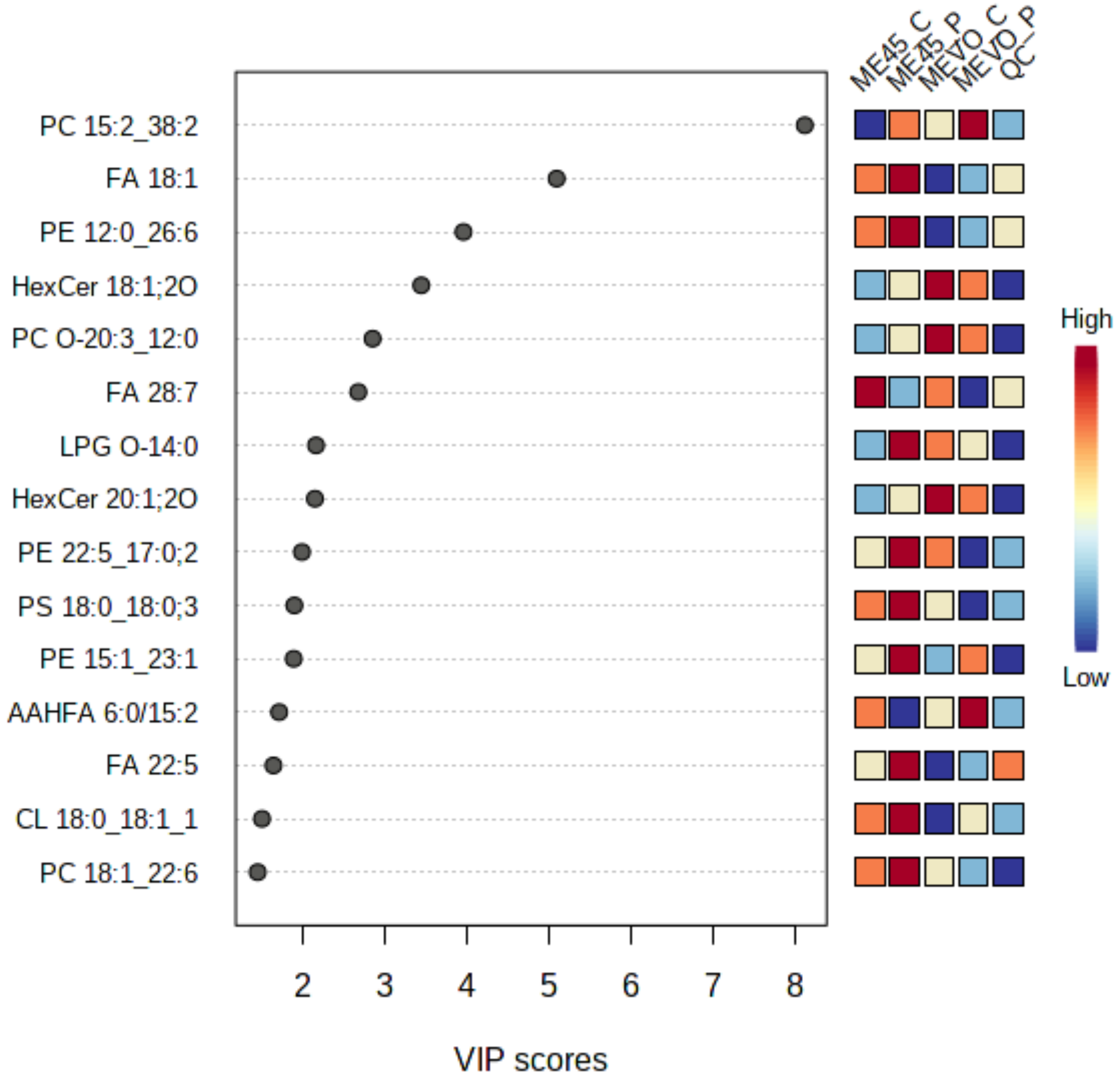

D

Fig. 9. PLS-DA variable importance in projection (VIP) score derived from the lipids profiling data of lipids with positive charge (A) A375, C32, ME45, MeWo of lipids with negative charge (B) A375, C32, ME45, MeWo, (C) joined group (lipids with positive and negative charge), and (D) ME45 and MeWo joined group. The colored boxes on the right indicate the relative concentrations of the corresponding metabolite in each group under study.
